# Deciphering Genomic Regions for High Grain Iron and Zinc Content Using Association Mapping in Pearl Millet

**DOI:** 10.3389/fpls.2017.00412

**Published:** 2017-05-01

**Authors:** N. Anuradha, C. Tara Satyavathi, C. Bharadwaj, T. Nepolean, S. Mukesh Sankar, Sumer P. Singh, Mahesh C. Meena, Tripti Singhal, Rakesh K. Srivastava

**Affiliations:** ^1^Division of Genetics, ICAR-Indian Agricultural Research InstituteNew Delhi, India; ^2^Division of Soil Science and Agricultural Chemistry, ICAR-Indian Agricultural Research InstituteNew Delhi, India; ^3^International Crops Research Institute for the Semi-Arid TropicsPatancheru, India

**Keywords:** pearl millet, iron and zinc, association mapping, SSR, favorable alleles

## Abstract

Micronutrient malnutrition, especially deficiency of two mineral elements, iron [Fe] and zinc [Zn] in the developing world needs urgent attention. Pearl millet is one of the best crops with many nutritional properties and is accessible to the poor. We report findings of the first attempt to mine favorable alleles for grain iron and zinc content through association mapping in pearl millet. An association mapping panel of 130 diverse lines was evaluated at Delhi, Jodhpur and Dharwad, representing all the three pearl millet growing agro-climatic zones of India, during 2014 and 2015. Wide range of variation was observed for grain iron (32.3–111.9 ppm) and zinc (26.6–73.7 ppm) content. Genotyping with 114 representative polymorphic SSRs revealed 0.35 mean gene diversity. STRUCTURE analysis revealed presence of three sub-populations which was further supported by Neighbor-Joining method of clustering and principal coordinate analysis (PCoA). Marker-trait associations (MTAs) were analyzed with 267 markers (250 SSRs and 17 genic markers) in both general linear model (GLM) and mixed linear model (MLM), however, MTAs resulting from MLM were considered for more robustness of the associations. After appropriate Bonferroni correction, *Xpsmp* 2261 (13.34% *R*^2^-value), *Xipes* 0180 (*R*^2^-value of 11.40%) and *Xipes* 0096 (*R*^2^-value of 11.38%) were consistently associated with grain iron and zinc content for all the three locations. Favorable alleles and promising lines were identified for across and specific environments. PPMI 1102 had highest number (7) of favorable alleles, followed by four each for PPMFeZMP 199 and PPMI 708 for across the environment performance for both grain Fe and Zn content, while PPMI 1104 had alleles specific to Dharwad for grain Fe and Zn content. When compared with the reference genome Tift 23D_2_B_1_-P1-P5, *Xpsmp* 2261 amplicon was identified in intergenic region on pseudomolecule 5, while the other marker, *Xipes* 0810 was observed to be overlapping with aspartic proteinase (*Asp*) gene on pseudomolecule 3. Thus, this study can help in breeding new lines with enhanced micronutrient content using marker-assisted selection (MAS) in pearl millet leading to improved well-being especially for women and children.

## Introduction

There is an increasing global attention towards addressing micronutrient malnutrition as the health impairment caused due to poor quality diet is more wide spread than low energy intake (Murgia et al., 2012). Among all mineral micronutrients, iron and zinc deficiency accounts for more than two billion people globally (WHO, 2012). One-third of its share is from India alone (Barthakur, 2010). It is more prominent in inhabitants of low income countries who depend on cereal based low quality staple food (Tiwari et al., 2016). Anemia, caused by iron deficiency is the most common disorder in such countries and people, who consume low quality diet are prone to possible risk of child mortality and other physiological disorders (Tako et al., 2015). Zinc is also an important micronutrient, which is required for proper growth, whose deficiency leads to stunting, increased susceptibility to many infectious diseases, morbidity and low mental ability (Deshpande et al., 2013). One among successful approaches to alleviate hidden hunger is biofortification, so that micronutrients can be delivered effectively to rural poor residents who majorly depend on staple food crops (Bouis and Welch, 2010; Saltzman et al., 2013). This can be easily achieved through molecular breeding. The biofortified products are cost-effective, sustainable, and remain within the purchasing power of rural poor (Pfeiffer and McClafferty, 2007; Bouis and Welch, 2010).

Pearl millet [*Pennisetum glaucum* (L.) R. Br.] is a climate resilient cereal which can be grown in harsh and adverse agricultural environments with scanty rainfall and less access to irrigation and fertilizers (Vadez et al., 2014). It exhibits greater level of tolerance to drought, heat stress and high temperature (Shivhare and Lata, 2016). It is widely cultivated as a source of food and fodder in arid and semi-arid regions of sub-Saharan Africa and India (Rao et al., 2006). It is gifted with many nutritional qualities compared to other cereals. It contains high amount of fiber, α-amylose (Nambiar et al., 2011), metabolizable energy, protein, essential amino acid, macro and micro nutrients like phosphorus, magnesium, iron and zinc and thus ensuring food and nutritional security. Pearl millet flour is used in fortification of food to improve nutritional quality of food products (Sonkar and Singh, 2015). Hence, consumption of these products were suggested by experts/physicians to all age groups, pregnant and nursing women for their proper mental and physical development. Consumption of pearl millet also forestalls cancer and Type-2 diabetes (T2D) and for relieving of celiac and several other non-communicable diseases (Nambiar et al., 2011).

Iron deficiency is prevalent in many parts of Africa and Asia, especially in India. Pearl millet provides 80–85% of total calorie intake per day (Tako et al., 2015) in Sahel region of Africa. The National Family Health Survey (NFHS) studies conducted in India indicate that more than 50% women (adolescent girls, lactating, and pregnant women) are anemic in states of Rajasthan, Haryana, Gujarat, and Maharashtra (Press Information Bureau, 2013) where pearl millet is largely grown (90% of Indian total production) and consumed (Rao et al., 2006; Finkelstein et al., 2015). Pearl millet can contribute 30–40% of these essential micronutrients and forms the cheapest source of staple food for iron and zinc in its growing regions (Rao et al., 2006). Hence development of micronutrient dense varieties or hybrids of pearl millet and their consumption would meet the required recommended dietary allowance (RDA) for these micronutrients and also would pave way to development of biofortified pearl millet.

Screening of germplasm lines for enhanced grain micronutrients and mapping the related QTLs will help in selection of parental lines or donors for developing iron/zinc rich hybrids or varieties. Despite having knowledge on different genes involved for iron and zinc uptake to final sequestration in grain (Grotz and Guerinot, 2006; Krohling et al., 2016), mapping for these traits is limited in most of the cereal crops (Kumar et al., 2010, 2016; Jin et al., 2015; Yu et al., 2015; Crespo-Herrera et al., 2016).

Accumulation of these micronutrients in the grain/edible portion is a complex mechanism involving many genes and moreover influenced by environment. One approach to dissect QTLs is through association mapping. It is a substitute to QTL mapping (Gómez et al., 2011) which utilizes the principle of linkage disequilibrium (LD) to find significant association of a molecular marker/QTL with a trait (Flint-Garcia et al., 2003; Gupta et al., 2005). It offers a great deal of advantage over linkage mapping in terms of higher mapping resolution since it accounts for historical mutations and recombinations in lineages leading to identification of markers nearby causative genes (Liu et al., 2016). More number of alleles can be considered, as broader population is included for investigation and allele mining can be attempted by exploitation of genetic diversity in a reference population (Flint-Garcia et al., 2003). SSR markers often are preferred in association mapping studies compared to other markers because of their co-dominant inheritance, multi-allelic nature, high reproducibility, and genome wide coverage (Varshney et al., 2005). Theoretically, SSRs are more powerful in detecting QTLs compared to SNPs (Ohashi and Tokunaga, 2003). With this background, the present study was undertaken to examine localization of genomic regions linked with enhanced grain iron and zinc content and its association with SSRs and genic markers using association mapping.

## Materials and methods

### Plant material

The association mapping panel comprised of 130 diverse pearl millet lines including two checks, namely, ICTP 8203Fe and ICMB 98222 for grain iron and zinc content (Table S1). The association panel represents B- lines (seed parents), R- lines (restorers or pollen parents) and advanced breeding lines derived from different parts of India and some introduced selections from Africa.

### Field experiments and evaluation

The experiment was conducted at three diverse geographical locations, representing all the three pearl millet growing agro-climatic zones of India, (i) ICAR-Indian Agricultural Research Institute Research farm, New Delhi (28° 382′ N, 77° 802′ E) representing Zone A receiving more than 400 mm annual rainfall (ii) ICAR-National Bureau of Plant Genetic Resources, Regional Station farm, Jodhpur (26° 252′ N, 72° 992′ E) falling in zone A_1_ with annual rainfall less than 400 mm and (iii) ICAR-IARI Regional Centre farm, Dharwad (15° 212′ N, 75° 052′ E) from zone B (covering the southern peninsular India). In all locations, the trial was taken for 2 consecutive years (Rainy Season, 2014 and 2015). The planting of genotypes was taken up in an alpha-lattice design (Patterson and Williams, 1976), with three replications. Each replication comprised of 13 incomplete blocks with 10 entries in each block. Each accession was represented in 2 rows of 4 m length with a spacing of 50 cm between rows and 15 cm from plant to plant. All the experiments were managed as per recommended agronomic practices across the locations and years to raise a normal healthy crop.

### Estimation of iron and zinc contents in grain

Open-pollinated panicles from five representative plants were harvested for each accession at physiological maturity, threshed with a wooden mallet and then cleaned, while taking utmost care to avoid dust or metal contamination of the samples. Further, oven-dried samples were used on duplicate basis to estimate iron and zinc by di-acid digestion (Singh et al., 2005) followed by readings taken on an atomic absorption spectrometer (AAS, ZEE nit 700 tech Analytikjena). Genotypes, which recorded high iron content (>77 ppm, as this is the criterion given by Harvest Plus) were also tested for aluminum content in ICPMS (Nex ION 300X, Perkin Elmer USA) in order to determine, whether higher grain iron content could be due to contamination with soil, dust or is innate (Pfeiffer and McClafferty, 2007).

The data over locations and years were subjected to combined analysis of variance across the six environments using PROC GLM with random statement, considering environments as random and genotypes as fixed effect in SAS v9.2 (http://iasri.res.in/design). The adjusted means thus obtained after analysis were used to generate all the descriptive statistics using IBM SPSS v20. The broad-sense heritability was calculated as per the formula:

$$\begin{array}{lll} {\text{H}}^{2}\; & = & \;{\text{σ}}_{\text{G}}^{2}/\left({\text{σ}}_{\text{G}}^{2}\;+\;{\text{σ}}_{\text{e}}^{2}/\text{r}\right)\ldots \ldots \ldots \ldots \ldots \ldots .\text{for single environment}\\ {\text{H}}^{2}\; & = & \;{\text{σ}}_{\text{G}}^{2}/\left({\text{σ}}_{\text{G}}^{2}\;+\;{\text{σ}}_{\text{GE}}^{2}/\text{n }+\;{\text{σ}}_{\text{e}}^{2}/\text{nr}\right)\ldots \ldots ..\text{for multi environment} \end{array}$$

where, $\sigma_{\text{G}}^{2}$ is genotypic variance, $\sigma_{\text{GE}}^{2}$ is genotype × environment interaction variance, $\sigma_{\text{e}}^{2}$ is error variance, *n* is number of environments and *r* is number of replications.

Datasets for analysis at Delhi during 2014 and 2015 were referred as Del-14 and Del-15. Similarly, data of Dharwad in 2014 and 2015 as DW-14 and DW-15. Likewise, Jodhpur data during 2014 and 2015 as Jod-14 and Jod-15. Mean data over years in a single location as Y14-M and Y15-M. Mean data across locations in a year were referred as Del-M, DW-M, and Jod-M. Mean data over all the six environments as grand mean (GM). Altogether, 12 datasets were included for analysis with TASSEL using both general liner model (GLM) and mixed linear model (MLM), but the results of only MLM are presented here. Further, though STRUCTURE and neighbor-joining (NJ) results were similar, we chose Q matrix files over PCA files due to greater relevance of the former with the pedigree of population for MLM analysis.

### DNA extraction and genotyping

Pooled leaf samples from five plants for each genotype were collected at 2 weeks stage and genomic DNA of all genotypes in the association mapping panel was isolated using modified CTAB method (Murray and Thompson, 1980). The PCR was performed in a 10 μl volume consisting of 1.0 μl DNA (25 ng/μl), 1.0 μl 10 X Buffer (Banglore Genei), 0.60 μl dNTP (10 mM, Banglore Genei), 0.20 μl (25 mM MgCl_2_, Banglore Genei), 1.0 μl each forward and reverse primer (10 mM), 0.13 μl Taq polymerase (3 U/μl, Banglore Genei) and the final volume is made up to 10 μl with autoclaved distilled water for every reaction. Thermo cycling was carried out in 384 well-blocked thermal cycler machine (GenePro, Bioer Technology Co. Ltd.) with a thermal cycling program having initial denaturation for 5 min at 94°C, followed by 40 cycles of 30 s at 94°C for denaturation, 30 s at temperatures as indicated in Table S2, extension at 70°C for 30 s and finally, 7 min at 72°C with final extension.

### Analysis of molecular data

#### Population structure and genetic relatedness

Initially, 114 polymorphic SSRs distributed in the entire genome was utilized for population structure and familial relatedness analysis. To realize the population structure, a Bayesian model-based program implemented in STRUCTURE v2.3.4 as suggested by Pritchard et al. (2000) was used with number of sub-populations (K) set from 1 to 12 with burn-in length and MCMC both set to 2,00,000. By using admixture model with correlated allele frequencies, K is replicated 10 times. The best *K*-value could not be determined easily by considering the log likelihood value [LnP(D)] of STRUCTURE output, hence, an *ad-hoc*, ΔK (Evanno et al., 2005) was determined to reveal number of sub-groups by importing structure output result zip file as input in the structure harvester program (http://taylor0.biology.ucla.edu/structureHarvester/index.php). Genotypes with *Q* ≥ 0.6 were allotted to corresponding sub-groups A, B, or C, and those with values <0.6 were allotted to a mixed sub-population, admixture. Genetic relatedness or K matrix is generated from TASSEL *v*3 (Trait Analysis by Association Evolution and Linkage, Bradbury et al., 2007).

Population grouping pattern obtained from STRUCTURE was further supported with the neighbor-joining (NJ) tree based on Nei's genetic distance (Nei, 1972) among the genotypes and also supplemented by principal coordinate analysis (PCoA) using DARwin v6 (Perrier et al., 2003). AMOVA was attempted by Arlequin (Excoffier and Lischer, 2010) by considering A, B, C, and admixture sub populations obtained in STRUCTURE.

### Linkage disequilibrium (LD) and marker-trait association (MTA)

For association mapping analysis, a total of 267 polymorphic markers were used of which 250 were SSR markers and 17 were genic markers. To target genes involved in the pathway of iron and zinc uptake from soil to final sequestration into grains by plants, 55 genic primers were screened for polymorphism. Twenty primer pairs were selected from previous studies on maize (Mondal et al., 2014) and rice (Sperotto et al., 2010). Further, orthologous genes were searched in phytozome database of other related crops for cross amplification in pearl millet, since the genome sequencing in this crop (http://ceg.icrisat.org/ipmgsc/) is still underway and not available in public domain. After identification of orthologous genes in foxtail millet and sorghum genome, a total of 35 gene specific primers (foxtail millet: 22 and sorghum: 13) were designed with the help of Primer3Plus web application (http://primer3plus.com/cgi-bin/dev/primer3plus.cgi). Out of 55 genic markers used, 17 were observed to be polymorphic and they were further utilized in the study. TASSEL *v*3 (Bradbury et al., 2007) with 1,000 permutations was used to generate an LD plot with *r*^2^ and *p*-values among all the 267 polymorphic markers. MTA was studied in TASSEL v3 (http://www.maizegenetics.net) with both general linear model (GLM) and mixed linear model (MLM) (Yu et al., 2006) as given below (Tadesse et al., 2015).

$$\begin{array}{lll} \text{y} & = & \text{Xa }+\text{ Qb }+\text{e }\ldots \ldots \ldots \ldots \ldots \ldots \ldots \ldots \text{GLM and}\\ \text{y} & = & \text{Xa }+\text{ Qb }+\text{ Zu }+\text{ e }\ldots \ldots \ldots \ldots \ldots \ldots ..\text{ MLM} \end{array}$$

where,

y is phenotype vector, a is marker vector with fixed effects, b is a vector with fixed effects, u is a vector with random effects (kinship matrix), e is a residuals vector, X denotes the accessions/genotypes at the marker, Q is the Q-matrix, result of STRUCTURE software, Z is an identity matrix.

A widely adopted threshold for the significance, Bonferroni correction (Bland and Altman, 1995; Li C. Q. et al., 2016) was used uniformly for all traits to observe association between trait and markers. In this context, *p* < 3.74 × 10^−3^ (where, *p* = 1/*n* and *n* = number of total markers used (267); also −log10 (1/267) = 2.43). Quantile-quantile (QQ) plots, plotted against expected and observed *p*-values to assess the adequacy of Type I error were generated with TASSEL (Figures S1, S2).

The position of the marker trait associations were determined following the published consensus linkage map of pearl millet by Rajaram et al. (2013).

Further, the significantly associated markers were tested for associations at individual sub-population level using one way ANOVA (http://vassarstats.net/anova1u.html), assuming null hypothesis of no phenotypic difference between different alleles of a marker.

### Favorable allele mining

Initially, Breseghello and Sorrells (2006) utilized null alleles (missing and rare alleles were also included) to calculate the phenotypic effect of an allele for identified MTAs. But all SSR markers may not have null alleles, sometimes rare alleles themselves are superior alleles. Hence, population mean was introduced in place of null alleles (Cai et al., 2014). Favorable allele for a marker loci associated with specific trait was identified using the formula (Cai et al., 2014; Li X. et al., 2016).

$$\begin{array}{l} a_{i}=\frac{\Sigma x_{ij}}{n_{i}}-\frac{\Sigma N_{k}}{n_{k}} \end{array}$$

where,

*a*_*i*_ is ith allele phenotypic affect, *x*_*ij*_ is phenotypic value of ith allele in jth genotype, *n*_*i*_ is number of accessions with ith allele, *N*_*k*_ is phenotypic value over all genotypes and *n*_*k*_ is total number of genotypes. When, *a*_*i*_ > 0, then this allele is said to have positive effect on the trait. When *a*_*i*_ < 0, the allele gives a negative effect (Zhang et al., 2013). Since, positive alleles give an increment, we considered superior positive alleles for planning crosses to pyramid maximum possible number of favorable alleles together for enhancement of grain iron and zinc content.

### Validation of MTAs obtained in association mapping

Four lines having higher mean iron and zinc content (PPMI 1102, PPMI 708, PPMI 683, PPMI 1107) and check (ICTP 8203Fe) along with two lines (5540B and PPMI 1155) with low mean iron and zinc content were selected for validation. Three consistently associated SSR marker primers, IPES 0096, IPES 0180, and PSMP 2261 were used to amplify DNA of all the seven selected genotypes. The amplified products were sequenced by standard Sanger's sequencing method by M/S Chromous Biotech, Bangalore. The sequenced reads were compared using BLAST against recently sequenced pearl millet reference genome,Tift23D_2_${\text{B}}_{1}^{-}$P1-P5 (unpublished) using MEGA6 (Tamura et al., 2013).

## Results

### Molecular genetic diversity analysis in association mapping panel

Initially, 114 polymorphic SSR markers, covering the entire genome were used to characterize pearl millet association panel to assess the genetic relationship. A total of 294 alleles were detected with an average number of 2.65 alleles per locus (Table S3). The observed number of alleles (n_a_) ranged from 2 to 5. Maximum number of five alleles were detected by genomic SSR loci, *Xpsmp* 2081. Four alleles per loci were observed in *Xipes* 200, *Xipes* 126 and *Xpgird* 50, and *Xpsmp* 2086. The mean effective allele number per loci (n_e_) for 114 polymorphic markers studied was 1.64. This shows that there exists a difference of 37.89% in observed and expected number of alleles. The discrepancy observed between n_a_ and n_e_ indicates that alleles detected were having frequencies <5% (Sehgal et al., 2015). The average major allele frequency was 0.75 with a range of 0.32 (*Xpsmp* 2066) to 0.98 (*Xipes* 0079. Measure of gene diversity, Nei's gene diversity (Nei) was calculated across the population. *Xipes* 0079 had the least value of 0.03 while highest value (0.73) was recorded by *Xpsmp* 2066 with a mean of 0.35. A frequently used measure to know the polymorphism information of a marker, PIC ranged from 0.03 (*Xipes* 0079) to 0.68 (*Xpsmp* 2066). In this study, seven SSRs (*Xpsmp* 2066, *Xpsmp* 2081, *Xpsmp* 2263, *Xpsmp* 2086, *Xipes* 0200, *Xipes* 0163, and *Xicmp* 3066) had a PIC value more than 0.5 and in total 67 markers were showing moderate PIC values.

### Population structure and cluster analysis

**Figure 1:**
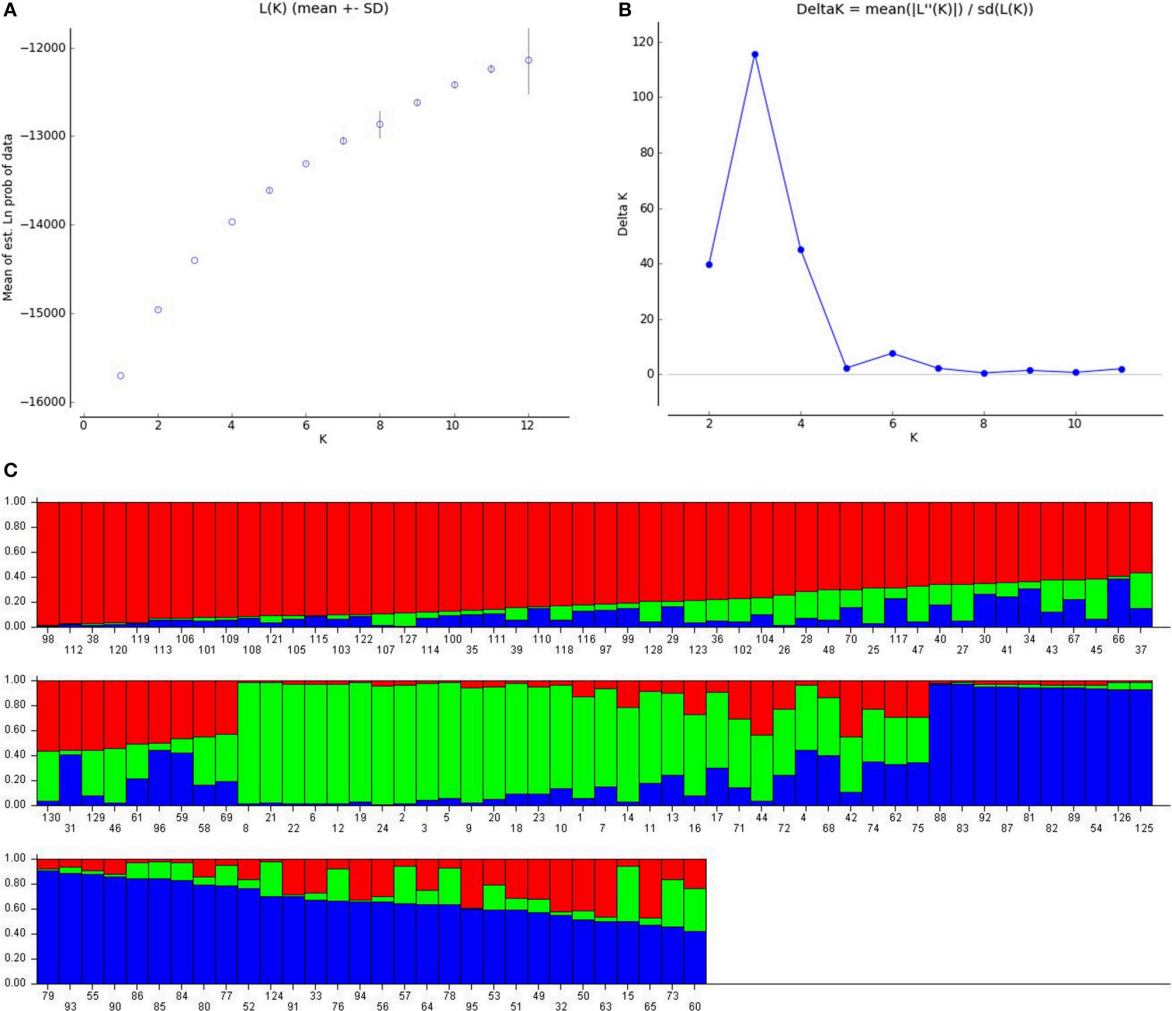
**Population stratification of pearl millet association mapping panel (A)** LnP(D), the log likelihood values from *k* = 1 to 12. **(B)** Delta K, rate of change from 2 to 11. **(C)** Inferred population structure (*K* = 3, each color represents one sub-population).

The true association is known only after considering population structure (Yu et al., 2006). A Bayesian model based approach, STRUCTURE, assigns individuals to sub-population based on genotyping. Pritchard et al. (2000) was of the opinion that, actual number of sub-populations in a panel may not be easy to identify, but one should consider the smallest *K*-value which captures the major composition in the population. Evanno et al. (2005) suggested the use of Δ*K*-value based on standard deviation to find the true value of K. The LnP(D) graph (Figure 1A) depicted with number of sub-populations(K) on x-axis and logarithmic of probability distribution on y-axis did not give a clear picture of the true value of *K*, so Δ*K* value was plotted against number of sub-population (Figure 1B) showed the highest peak at *K* = 3, which indicated that there are three sub-populations in the panel. The genotypes were assigned to individual sub-population keeping in view of highest membership likelihood criterion based on *Q*-values obtained from STRUCTURE software. The decision point taken here is, a genotype indicating a *Q*-value more than or equal to 0.6 is assigned to that particular sub group A or B or C and remaining genotypes which did not reach *Q* = 0.6 were allotted to admixture group (Table S1). In this manner, the first sub-population A is the biggest group containing 50 lines, B had 22, and C had 31, while remaining genotypes (30) were kept in admixture. These three sub populations were represented by three colors in the bar plot showing three sub-populations (Figure 1C). The sub population A is depicted by red, B by green, and C by blue color. Here, sub-population A is said to have fewer admixtures (0.19%) compared to B (0.29%) or C (0.21%) and in overall 23.08% of total population is under admixture. Sub-population A consisted of genotypes from different sources of pedigree, which included B lines while sub-population B comprised of few promising lines derived from a cross between, PPMI 683 and PPMI 627, few lines from 843B × 841B lines and some low iron Jamnagar lines and few other breeding lines. Sub-population C, majorly included genotypes selected for good agronomic score in a cross between PPMI 683 and PPMI 627, few lines derived from a cross between WGI 148 and WGI 52, and few others. Admixture consisted of thick panicle restorer lines, early maturing lines and dual purpose lines.

**Figure 2:**
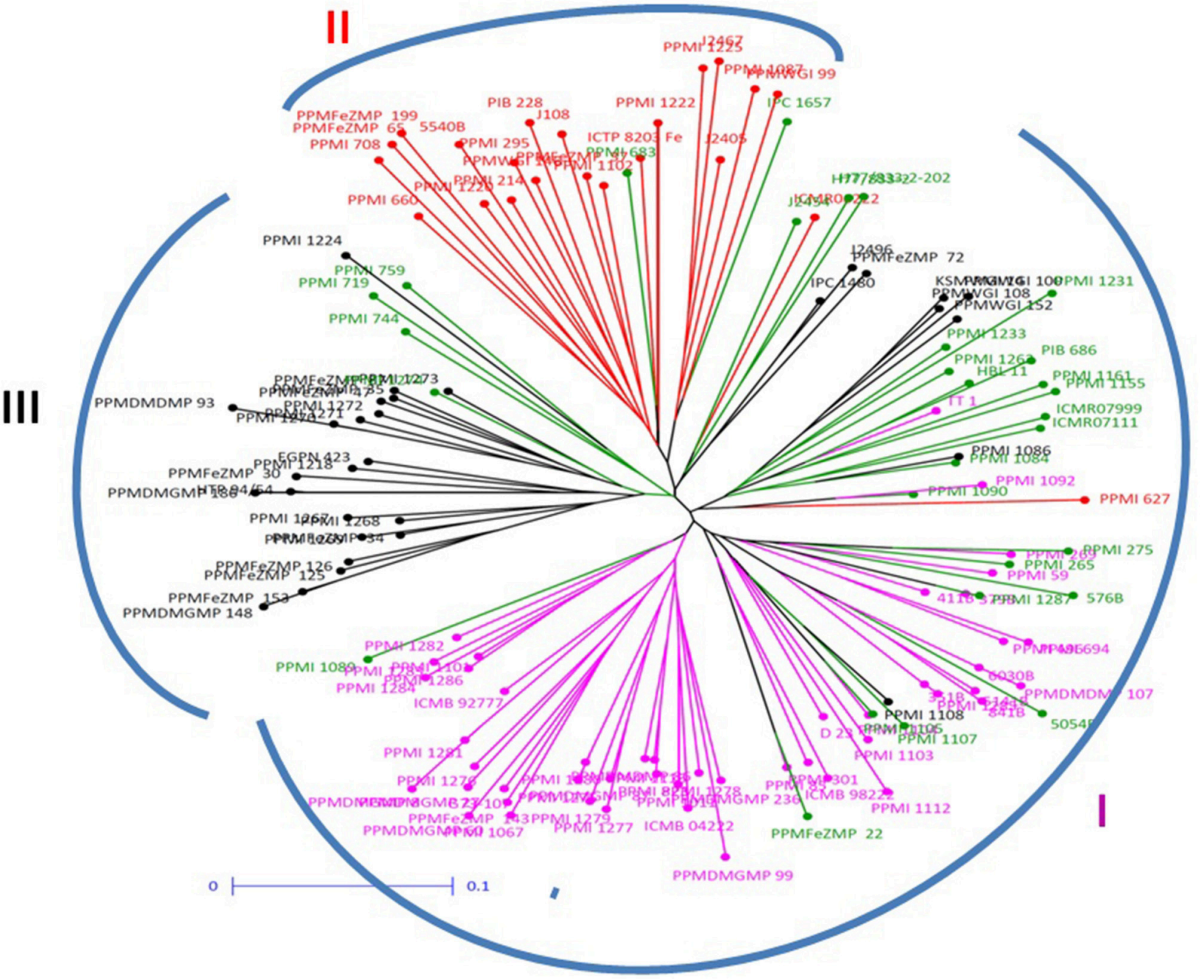
**Neighbor joining tree constructed for pearl millet association mapping panel (genotypes represented in different colors corresponding to the three sub-populations and admixture observed in STRUCTURE)**.

The dendrogram generated by DARwin using neighbor joining, sub-classified population into three groups. The maximum number of individuals was clustered in group 1 consisting 75 genotypes (Figure 2), whereas group 2 consisted of 29 and group 3 comprised of 26 individuals. Cluster 1 was only 64.0% similar to sub-population A, whereas Cluster 2 had 72.4% similarity with sub-population B while cluster 3 shared 88.5% similarity with sub-population C.

**Figure 3:**
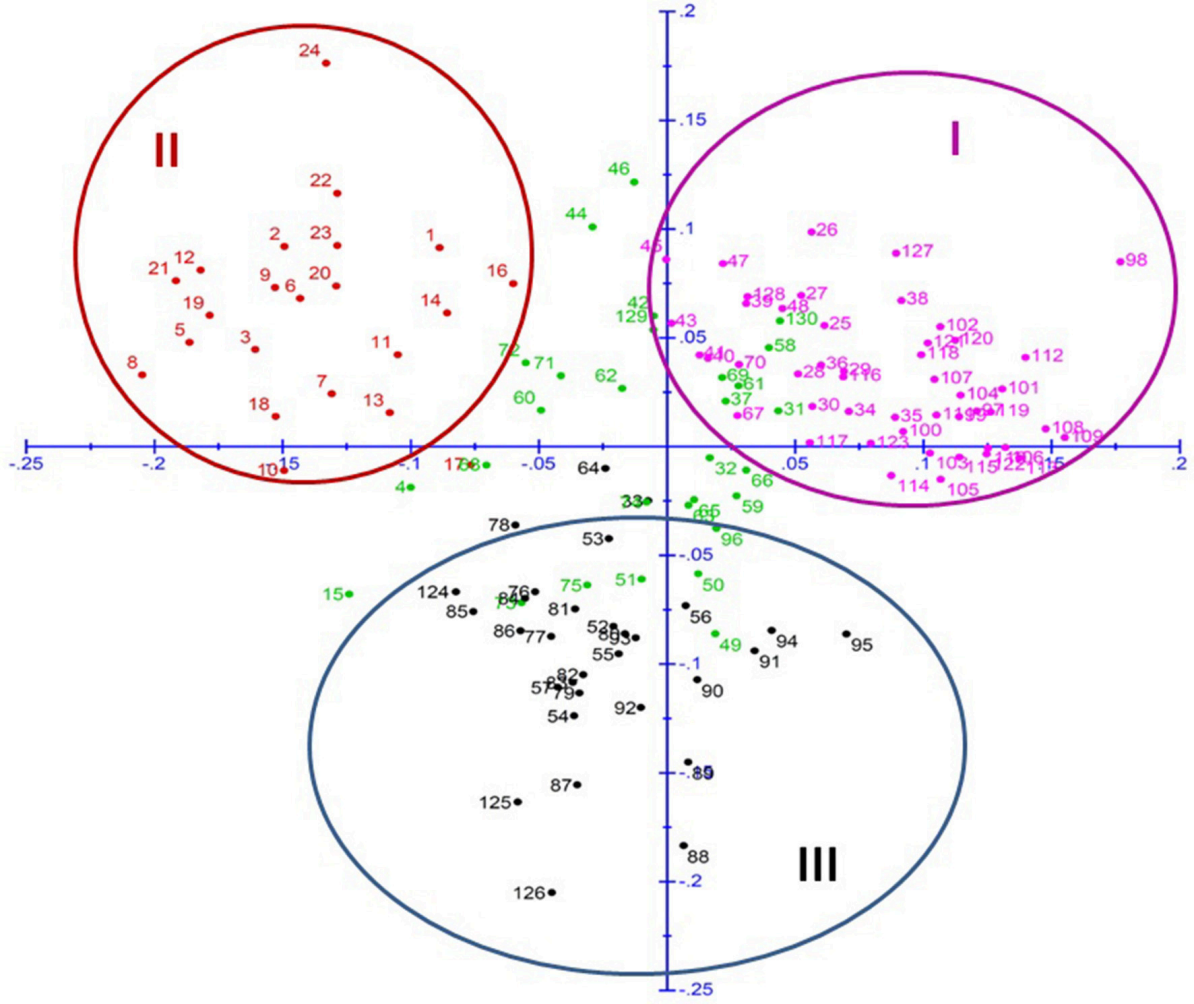
**Principal Coordinate Analysis (PCoA) of pearl millet association mapping panel (genotypes represented in different colors corresponding to the three sub-populations plus admixture observed in STRUCTURE)**.

The principal coordinate analysis further supported this clustering pattern, where all the breeding lines were distributed in all four quadrants (Figure 3). Cluster I, similar to sub-population A of STRUCTURE analysis was observed in first quadrant. In same way, cluster II, similar to sub-population B fell in second quadrant and cluster III, similar to sub-population C, was mostly in third and a little of fourth quadrant. Remaining genotypes which belong to admixture in STRUCTURE were dispersed throughout all the quadrants.

Results from AMOVA showed that significant differences exist between sub populations which accounted for 11.8% of total variation. Within sub-population variation was much larger, contributing 82.2% to total variation and within individual variation was 6.0% (Table 1).

**Table 1:** **AMOVA between sub-populations and genotypes**.

**Source of variation**	**Degrees of freedom**	**Sum of squares**	**Variance components**	**Percentage of variation**
Among sub-populations	3	523.86	2.24 Va	11.77
Among individuals within sub-populations	126	4,095.14	15.68 Vb	82.20
Within individuals	130	149.50	1.15 Vc	6.03
Total	259	4,768.49	19.07	

*Va, Vb, and Vc all are significant at p < 0.01*.

### Variation for grain iron and zinc content in the association mapping panel

Pearl millet grain iron and zinc content were having a wide range of variation at all six environments studied showing significant differences between genotypes in the analysis of variance (Tables S4A–E). Grain iron content (grand mean) ranged from 32.3 to 111.9 ppm whereas grain zinc content ranged from 26.6 to 73.7 ppm (Tables 2A,B, Tables S5A,B). The broad sense heritability estimates were high (>0.8) at all the individual environments (Tables 2A,B). It ranged from 0.84 (Jod-15) to 0.89 (DW-15) for grain zinc content while for grain iron content it was 0.85 (Del-14) to 0.92 (Jod-14). In pooled environments, the highest heritability value was recorded at Dharwad location (0.86) while the lowest during the year 2015 (0.60) across all the three locations for grain zinc content with 0.79 combined over all six environments. Grain iron content exhibited the highest heritability at Delhi location (0.8) while the lowest at Dharwad location (0.65). Hence, these results indicate that heritability was high for both grain iron and zinc content.

**Table 2A:** **Descriptive statistics and heritability of grain iron content at six individual environments and their six pooled environments (ppm)**.

	**Del-14**	**Del-15**	**Jod-14**	**Jod-15**	**DW-14**	**DW-15**	**Y14-M**	**Y15-M**	**Del-M**	**Jod-M**	**DW-M**	**GM**
Minimum	27.29	29.99	23.27	24.64	31.22	34.36	29.00	34.73	29.27	29.65	36.02	32.30
Maximum	125.01	117.27	121.63	111.40	139.12	123.78	114.60	112.10	116.40	109.62	122.34	111.90
Mean	57.52	60.44	55.73	57.81	60.71	61.49	57.99	59.92	58.98	56.77	61.10	58.95
SEm	1.54	1.43	1.80	1.43	1.99	1.51	1.38	1.13	1.34	1.38	1.48	1.13
H^2^	0.85	0.87	0.92	0.91	0.94	0.88	0.67	0.68	0.80	0.72	0.65	0.79

*Where, Del-14, Del-15, Jod-14, Jod-15, DW-14, DW-15, Y14-M, Y15-M, Del-M, Jod-M, DW-M, and GM are Delhi during 2014, Delhi during 2015, Jodhpur during 2014, Jodhpur during 2015, Dharwad during 2014, Dharwad during 2015, Year 2014 mean, Year 2015 mean, Delhi mean, Jodhpur mean, Dharwad mean, and grand mean, respectively. SEm and H^2^ are standard error mean and heritability, respectively*.

**Table 2B:** **Descriptive statistics and heritability of grain zinc content at six individual environments and their six pooled environments (ppm)**.

	**Del-14**	**Del-15**	**Jod-14**	**Jod-15**	**DW-14**	**DW-15**	**Y14-M**	**Y15-M**	**Del-M**	**Jod-M**	**DW-M**	**GM**
Minimum	27.90	23.40	20.30	22.29	21.70	19.12	26.40	26.64	28.50	24.85	21.01	26.62
Maximum	86.20	78.38	86.00	84.36	73.27	74.22	77.20	74.19	81.08	81.71	70.65	73.68
Mean	46.61	49.06	42.75	45.16	36.40	35.74	41.92	43.32	47.83	43.96	36.07	42.62
SEm	1.04	1.11	1.14	1.05	0.95	1.16	0.78	0.83	1.00	1.01	0.99	0.76
H^2^	0.85	0.86	0.87	0.84	0.87	0.89	0.68	0.60	0.83	0.80	0.86	0.79

*Where, Del-14, Del-15, Jod-14, Jod-15, DW-14, DW-15, Y14-M, Y15-M, Del-M, Jod-M, DW-M, and GM are Delhi during 2014, Delhi during 2015, Jodhpur during 2014, Jodhpur during 2015, Dharwad during 2014, Dharwad during 2015, Year 2014 mean, Year 2015 mean, Delhi mean, Jodhpur mean, Dharwad mean, and grand mean, respectively. SEm and H^2^ are standard error mean and heritability, respectively*.

### LD and association of markers with grain iron and zinc content in different environments

**Figure 4:**
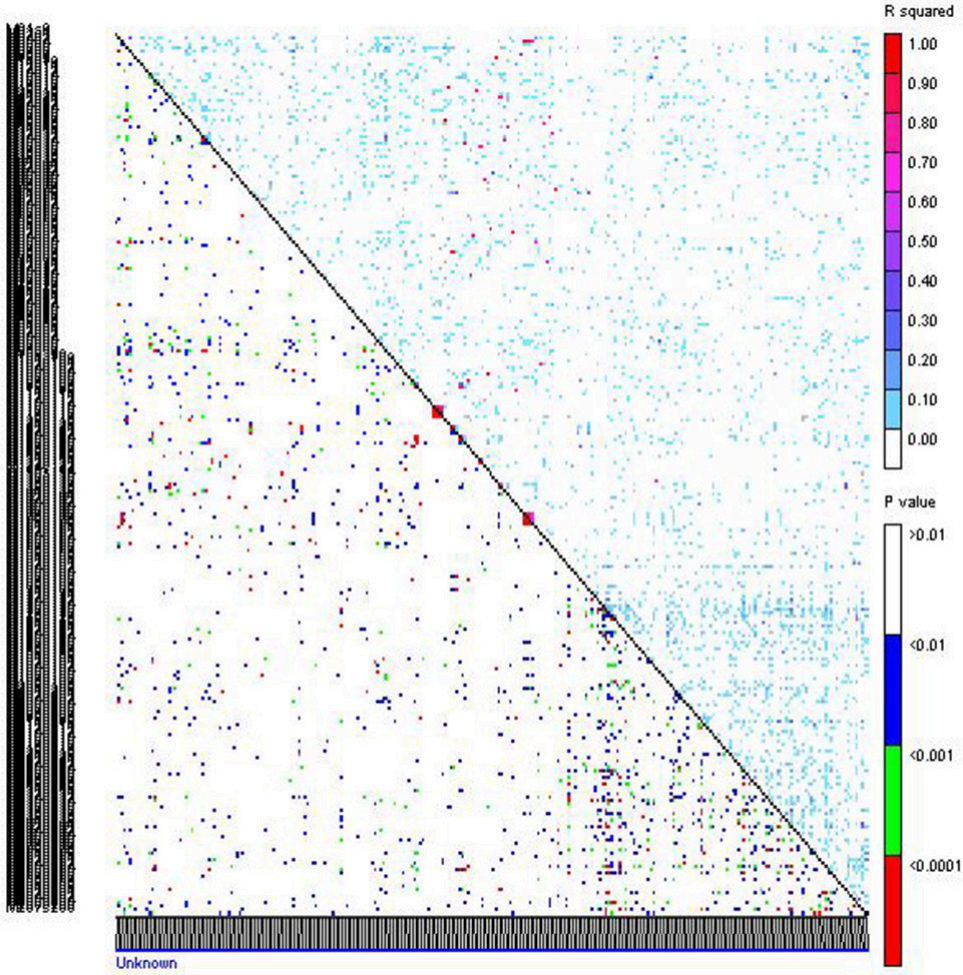
**Linkage disequilibrium pattern of association mapping panel genotyped with 267 markers**. Upper triangle represents *R*^2^ of markers and in lower triangle corresponding *p*-values are given.

The LD measured as squared value of Pearson correlation, *R*^2^ ranged from 0 to 1 with a mean of 0.017. Highest *R*^2^-value was observed between *Xpsmp* 2063 and *Xipes* 0145 on LG7, *Xipes* 0103 and *Xipes* 0146 of LG1 (Figure 4). Out of 35,511 pair-wise combinations obtained from 267 marker loci, 69 pairs had *R*^2^ more than 40%. Only 9.53% of the 35,511 pairs were in extent of LD (*p* < 0.05) across all the genotypes accessed. Before performing the association mapping, correlations between grain iron and zinc content and population structure was computed (Table S6). Grain iron was significantly associated with structure at DW-15, while grain zinc content recorded its significance at DW-14, DW-15, and DW-M. In remaining datasets, there is no correlation of the trait with the structure. The Q+K model holds good in all the cases, but when there is correlation of trait with the structure, Q+K model is a better model which avoids spurious associations (Yu et al., 2006). The threshold for identifying significant associations with a trait kept here was *p* < 0.0037, which was a Bonferroni corrected value considered by many workers in association mapping studies. Significant associations with grain iron and zinc content were tested across six different environments and their six means in different combinations.

### Environment-wise marker-trait associations—iron

The MLM analysis (Table 3) identified *Xipes* 0180 and *Xsinramp* 6 to be significantly associated with the trait explaining 12.4 and 10.1% variation, respectively, in Delhi, 2014. During the year 2015 at Delhi, *Xpsmp* 2261 recorded significant association with grain iron content. Mean of Delhi over 2 years, indicated *Xpsmp* 2261, *Xipes* 0096 and *Xsinramp* 6 to be highly associated with grain iron with an *R*^2^ more than 9.0%. Dharwad 2014 dataset analysis indicated significance of only one SSR marker (*Xicmp* 3092) with grain iron content, while during the year 2015 at Dharwad, *Xpsmp* 2209 was observed to be associated. No association was observed for grain iron content at Dharwad over mean of 2 years.

**Table 3:** **Markers associated with grain iron and zinc content along with phenotypic variance explained at *p* < 0.0037 (Bonferroni corrected *p*-value) in MLM**.

**Data set**	**Marker**	**Fe**		**Zn**	
		**marker_p**	**Marker *R*^2^**	**marker_p**	**Marker *R*^2^**
Del-14	*Xipes* 0180	0.0017	0.124	ns	ns
	*Xpsmp* 2261	ns	ns	0.0021	0.106
	*Xsinramp* 6	0.0036	0.101	ns	ns
Del-15	*Xicmp* 3016	ns	ns	0.0032	0.082
	*Xpsmp* 2261	6.00 × 10^−4^	0.122	ns	ns
Del-M	*Xipes* 0096	0.0012	0.11	ns	ns
	*Xipes* 0180	0.0036	0.102	ns	ns
	*Xpsmp* 2261	0.0034	0.92	0.0031	0.099
	*Xsinramp* 6	0.0033	0.098	ns	ns
DW-14	*Xicmp* 3092	0.0036	0.091	ns	ns
	*Xipes* 0096	ns	ns	4.56 × 10^−4^	0.127
	*Xpsmp* 2086	ns	ns	0.0015	0.108
	*Xsinramp* 6	ns	ns	0.002	0.107
DW-15	*Xicmp* 4006	ns	ns	0.0017	0.104
	*Xpsmp* 2209	0.0034	0.07	ns	ns
	*Xsinramp* 6	ns	ns	0.0012	0.119
DW-M	*Xipes* 0096	ns	ns	0.003	0.094
	*Xsinramp* 6	ns	ns	6.17 × 10^−4^	0.13
Jod-14	*Xicmp* 3004	ns	ns	0.0023	0.103
	*Xipes* 0096	0.0024	0.098	ns	ns
	*Xipes* 0224	ns	ns	0.0017	0.105
	*Xpsmp* 2261	8.1 × 10^−5^	0.157	3.61 × 10^−4^	0.131
Jod-15	*Xipes* 0180	ns	ns	0.0032	0.095
	*Xpsmp* 2261	6.44 × 10^−4^	0.121	6.14 × 10^−5^	0.162
Jod_M	*Xicmp* 3004	ns	ns	0.0047	0.091
	*Xpsmp* 2213	ns	ns	2.09 × 10^−4^	0.141
	*Xpsmp* 2261	2.22 × 10^−5^	0.181	5.35 × 10^−5^	0.165
Y14-M	*Xipes* 0096	7.42 × 10^−4^	0.118	6.43 × 10^−4^	0.121
	*Xipes* 0180	0.0024	0.118	ns	ns
	*Xpsmp* 2261	3.28 × 10^−4^	0.133	2.11 × 10^−4^	0.14
Y15-M	*Xipes* 0096	0.0017	0.104	0.0017	0.104
	*Xpsmp* 2261	2.17 × 10^−4^	0.14	0.0014	0.107
GM	*Xipes* 0096	1.36 × 10^−4^	0.148	9.41 × 10^−4^	0.114
	*Xipes* 0180	9.89 × 10^−4^	0.131	ns	ns
	*Xpsmp* 2261	1.7 × 10^−4^	0.144	3.00 × 10^−4^	0.134

*Where Del-14, Del-15, Jod-14, Jod-15, DW-14, DW-15, Y14-M, Y15-M, Del-M, Jod-M, DW-M, and GM are Delhi during 2014, Delhi during 2015, Jodhpur during 2014, Jodhpur during 2015, Dharwad during 2014, Dharwad during 2015, Year 2014 mean, Year 2015 mean, Delhi mean, Jodhpur mean, Dharwad mean, and grand mean, respectively. ns, non-significant*.

At Jodhpur during the year 2014, *Xpsmp* 2261 and *Xipes* 0096 were observed to be associated with the trait, where *Xpsmp* 2261 explained phenotypic variance of 15.7%, while another marker, *Xipes* 0096 recorded more than 8.0% variation. *Xpsmp* 2261 showed statistical significance by explaining more than 10% variation in the year 2015 at Jodhpur. Mean iron content of Jodhpur over 2 years revealed only one marker locus, *Xpsmp* 2261 to associate with the trait by explaining 18.1% variation.

During 2014, the data pooled over locations identified *Xpsmp* 2261, *Xipes* 0096, and *Xipes* 0180 to associate with grain iron content. Similarly, during year 2015, *Xpsmp* 2261 and *Xipes* 0096 were observed to be significantly associated with the trait. Likewise, three SSR loci, *Xpsmp* 2261, *Xipes* 0096, and *Xipes* 0180 were recorded to be associated with grain iron content in grand mean data set.

### Association with grain zinc content

During 2014 at Delhi, *Xpsmp* 2261 recorded significant association with grain zinc content explaining variance of more than 10% (Table 3). MLM analysis of the data during the year 2015 at Delhi, showed only one marker, *Xipes* 3016 to be significantly associated with grain zinc with more than 8.0% variation explained in the model, while *Xpsmp* 2261 was observed to be significantly associated with the mean over 2 years at Delhi. At Dharwad, during the year 2014, *Xipes* 0096 was observed to be associated with 12.7% variation, whereas, during the year 2015 at Dharwad, *Xicmp* 4006 revealed its significance with an *R*^2^ of 10.4%, while *Xipes* 0096 and *Xsinramp* 6 were recorded as significant for Dharwad mean over 2 years.

Jodhpur, in the year 2014 recorded three SSRs (*Xicmp* 3004, *Xipes* 0224, and *Xpsmp* 2261) to be significantly associated with grain zinc content explaining more than 10% variation. In the year 2015 at Jodhpur, *Xpsmp* 2261 and *Xipes* 0180 were associated significantly, whereas the mean of Jodhpur over 2 years revealed three SSR markers (*Xicmp* 3004, *Xpsmp* 2213, and *Xpsmp* 2261) to be associated with grain zinc content. Mean zinc content of the years, 2014 and 2015 revealed *Xipes* 0096 and *Xpsmp* 2261 to be associated significantly with the trait explaining more than 10% variation each. When association were observed with the grand mean, over all locations and years, *Xipes* 0096 and *Xpsmp*2261 were found to be significantly associated with grain zinc content with *R*^2^ more than 11.0% individually.

### Frequency of significant markers in MTAs with grain iron and zinc content

Table S7A shows the number of times a marker was observed as significantly associated with grain iron or zinc content *via* MLM analysis. Out of total 6 markers identified to be associated with grain iron content, *Xpsmp* 2261 recorded significance in highest number of times (Del-15, Jod-14, Jod-15, Del-M, Jod-M, Y14-M, Y15-M, and GM). Further, higher number of associations were observed with *Xipes* 0096 which recorded as significant in five datasets (Del-M, Jod-14, Y14-M, Y15-M, and GM) followed by *Xipes* 0180 which recorded significant associations in four datasets (Del-14, Del-M, Y14-M, and GM). Genic marker, *Xsinramp* 6 identified to be associated with iron content in two datasets (Del-14 and Del-M) while *Xicmp* 3092 and *Xpsmp* 2209 were significant at Dharwad location during the year 2014 and 2015, respectively.

For grain zinc content (Table S7B), among 10 markers identified as significantly associated with the trait, *Xpsmp* 2261 showed association in eight datasets (Del-14, Jod-14, Jod-15, Del-M, Jod-M, Y14-M, Y15-M, and GM). Next, *Xipes* 0096 was observed as significant for five times (DW-14, DW-M, Y14-M, Y15-M, and GM). Genic marker, *Xsinramp* 6 was associated with the trait in three datasets (DW-14, DW-15 and DW-M) while, seven SSR markers (*Xipes* 0180, *Xpsmp* 2086, *Xipes* 0224, *Xpsmp* 2213, *Xicmp* 3004, *Xicmp* 3016, and *Xicmp* 4006) showed significance only at one location.

**Figure 5:**
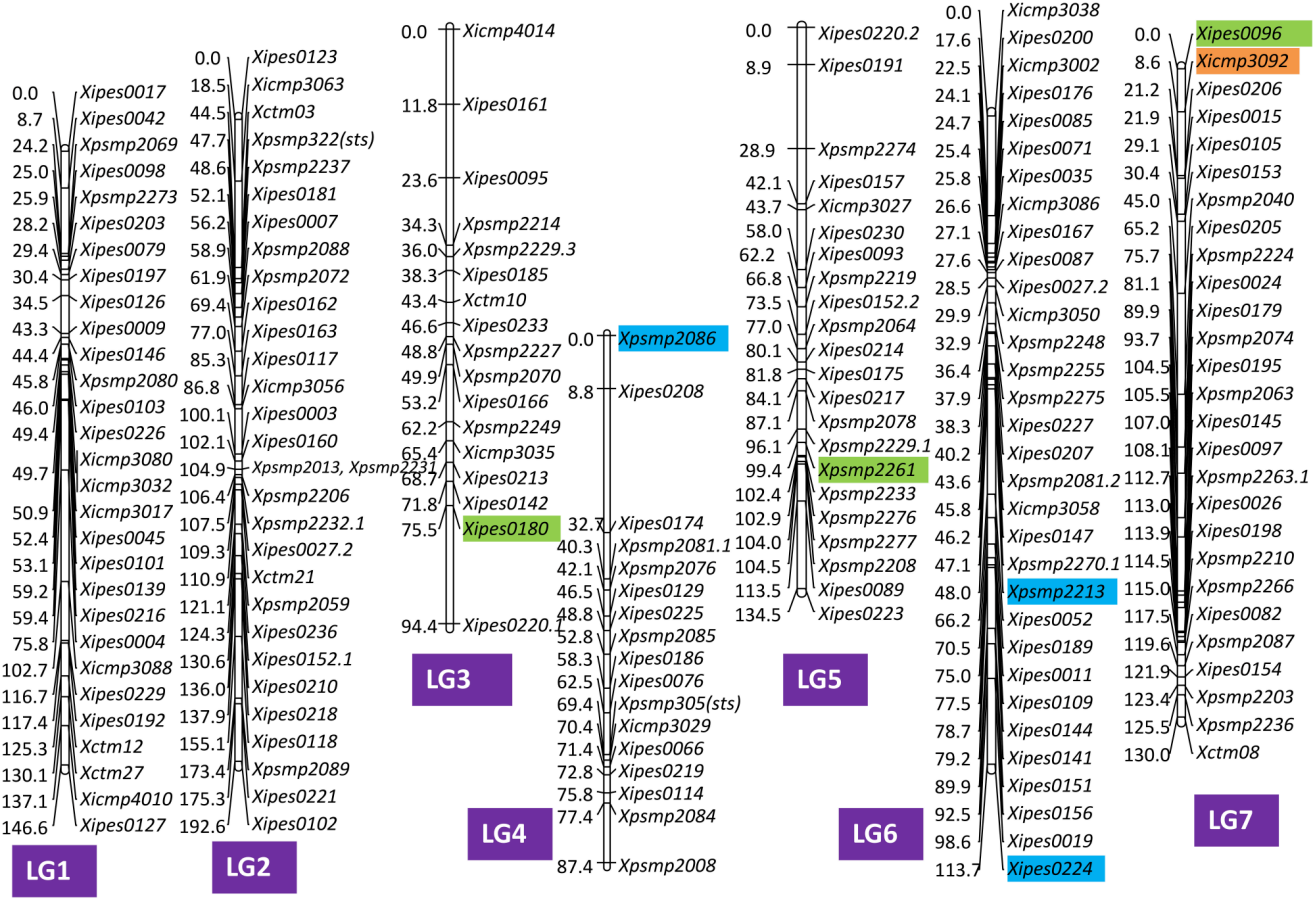
**Genomic positions of significantly associated SSR markers with grain iron and zinc content in the consensus map of Rajaram et al. (2013)**. Color code: Orange for iron; Blue for zinc; and Green for both iron and zinc.

For grain iron content alone, six markers were identified to be statistically significant at Bonferroni correction (*p* < 0.0037) by MLM analysis in all the combinations of environments studied (Table S8, Figure 5). Two SSR markers were from LG7 and one each marker belonging to LG3 and LG5 in the published pearl millet consensus map of Rajaram et al. (2013) were associated with iron content. Remaining two markers (*Xpsmp* 2209 and *Xsinramp* 6) were unmapped to any linkage group of the published consensus map of pearl millet.

For grain zinc content (Table S8, Figure 5), total 10 markers were observed to be associated in all the environments taken together. The maximum number of significant markers (4) were from unmapped markers (from Rajaram et al., 2013) followed by LG6, where *Xipes* 0224 and *Xpsmp* 2213 were observed to be significant. One each marker from LG3, LG4, LG5, and LG7 were observed to associate with grain zinc content in MLM.

In total, 12 markers (4 genomic SSRs, 7 EST-SSRs, and 1 genic marker) accounting for16 MTAs were observed to be significantly associated with either grain iron or zinc content at significant threshold of Bonferroni correction (*p* < 0.0037) using MLM analysis of data from different environments and combination of their means (Table S8, Figure 5). The maximum contribution was from LG6 and LG7, which included two SSRs each. Both of the markers from LG6 were associated with zinc content only, whereas one SSR, *Xipes* 0096 of LG7 was highly associated with both iron and zinc content, while another SSR, *Xicmp* 3092 was associated with iron content only. One SSR each from LG3, and LG5 were associated with both the traits. One genic marker, *Xsinramp* 6 also showed significance of association with both iron and zinc content. Remaining four unmapped markers in the published consensus map (Rajaram et al., 2013) were either associated with grain iron or zinc content.

### Marker-trait associations in sub-populations

Twelve significant markers resulted from MLM analysis were used to study their associations at sub-population level. For grain iron content, 13, 26, and 23 MTAs were recorded in sub-population A, B and C respectively (Table S9A). Similarly, 19, 24, and 18 MTAs were recorded for grain zinc content in sub-population A, B and C respectively (Table S9B). Maximum number of associations were observed in sub-population B for both traits, where most of promising lines like PPMI 1102, PPMFeZMP 199, PPMI 708 etc. were included.

*Xpsmp* 2261 at Del-15 and *Xsinramp* 6 at Del-M showed significant association with grain iron content in all the three sub-populations (comparing Table S9A with Table 3). Significance in at least two sub-populations were observed by *Xicmp* 3092 at DW-14; *Xipes* 0180 in Del-14 and GM; *Xsinramp* 6 in Del-14 and *Xpsmp* 2261 in Del-M, Jod-M, Y14-M, Y15-M, and GM datasets. Few associations *viz., Xipes* 0096 at Del-M, Jod-14, Y14-M, Y15-M, and GM; *Xipes* 0180 at Del-M and Y14; *Xpsmp* 2209 at DW-15; *Xpsmp* 2261 at Jod-14 and Jod-15 were restricted only to one sub-population, but resulted in significant associations using MLM analysis of the whole population. Few MTAs were not observed while analyzing the whole population but were recorded in two out of three sub-populations for some datasets.

Similarly for grain zinc content (comparing Table S9B with Table 3), *Xpsmp* 2261 in Y14-M, Y15-M, and GM; *Xsinramp* 6 in DW-M; *Xsinramp* 6 in DW-14 recorded associations in two sub-populations out of three, whereas *Xpsmp* 2261 in Del-14, Del-15, Jod-14, and Jod-M; *Xipes* 0096 in DW-14, Y14-M, Y15-M, and GM; *Xicmp* 3016 in Del-15; *Xipes* 0096 in DW-M; *Xipes* 0180 in Jod-15; *Xicmp* 4006 in DW-15; *Xicmp* 3004 and *Xipes* 0224 in Jod-14 datasets recorded significance only in one sub-population. MTAs, *Xpsmp* 2086 at DW-14; *Xsinramp* 6 in DW-15; *Xpsmp* 2213 in Jod-M did not show significance at sub-population level, though they are associated in the whole population. *Xpsmp* 2261 in DW-14 and DW-M; *Xipes* 0180 in Del-14 and *Xsinramp* 6 in Del-15 appeared in significant association with grain zinc content in two sub-populations, though they are not associated at whole population level.

### Identification of favorable alleles

Alleles having positive effect were considered as favorable for both grain iron and zinc content. Details of these alleles along with top three genotypes carrying them are given in Tables S10A,B. The favorable allele is expressed in terms of amplicon size of the SSR marker. A total of six alleles were detected for grain iron content, and five out of 10 alleles detected for grain zinc content had phenotypic effect of more than four. Highest phenotypic effect of alleles for grain iron and zinc content was observed by *Xpsmp* 2261-180. Among the top three performing genotypes, PPMI 1102 had highest number (7) of favorable alleles (Tables S11A,B), followed by PPMI 1104 (5), PPMFeZMP 199 (4), and PPMI 708 (4). PPMI 1102, PPMFeZMP 199, PPMI 708 and PPMI 683 had alleles which expressed across environments, whereas PPMI 1104 had *Xicmp* 3092-220 allele specific for Fe content, and *Xicmp* 4006-280 allele specific for Zn content at Dharwad.

### Preferred crosses to improve target traits

Considering the average phenotypic effect of an allele approximately equal to four or greater, nine better crosses (Table 4) were suggested to pyramid maximum number of alleles in a single genotype. To account for maximum pyramiding of alleles even having minor effect, one single cross (PPMI 1102 × PPMI 1104) can capture as many as 10 alleles favorable for both grain iron and zinc content.

**Table 4:** **Crosses proposed to accumulate favorable alleles for enhancing both grain iron and zinc content**.

**Crosses**	**Alleles predicted**		
	**Fe**	**Zn**	**Total**
PPMI 1102 × PPMI 1104	6	4	7
PPMI 1102 × PPMI 1116	5	5	6
PPMI 1102 × PPMI 1105	5	5	6
PPMI 1102 × PPMI 1231	6	3	6
PPMI 1102 × PPMDMGPM 186	6	3	6
PPMI 1102 × PPMI 265	6	3	6
PPMI 1102 × PPMI 1285	6	3	6
PPMFeZMP 199 × PPMI 1104	5	4	6
PPMI 1104 × PPMI 683	4	3	6

### Validation of marker-trait associations using bioinformatics tools

**Figure 6:**
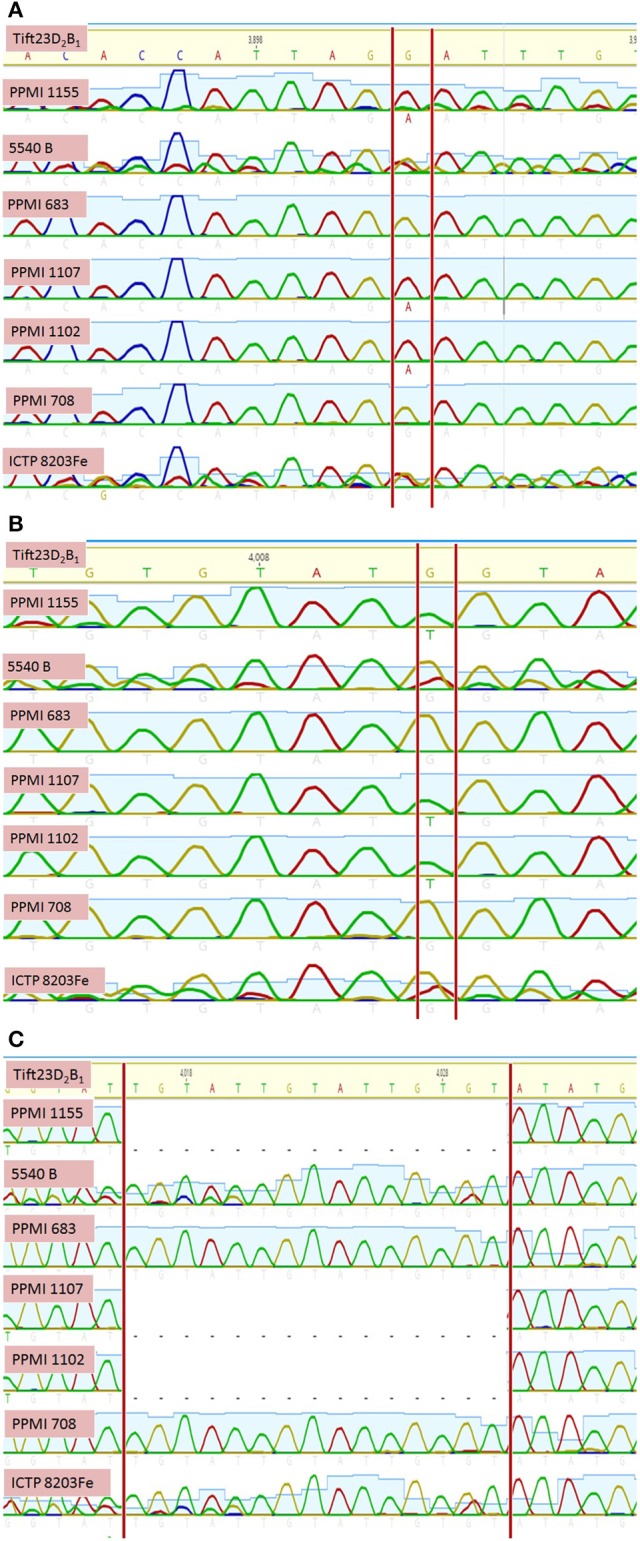
**Sequence homology of high and low grain iron and zinc genotypes with pearl millet reference sequence identifies putative (A)** SNP1, **(B)** SNP2, and **(C)** InDel.

Out of three SSR markers selected for amplifying seven genotypes, *Xipes* 0096 was dropped for further analysis, because of its poor read quality. The amplicons of *Xpsmp* 2261 and *Xipes* 0810 for seven genotypes were compared with reference genome Tift23D_2_${\text{B}}_{1}^{-}$P1-P5. Among two markers studied, *Xpsmp* 2261 amplicon landed in intergenic region on pseudomolecule 5 and was having >50% GC content, while the other marker, *Xipes* 0810 was observed to be overlapping with gene on pseudomolecule 3. This flanking gene was annotated to be aspartic proteinase (*Asp1*) gene. On comparison with the reference sequence, two putative SNPs (Figure 6) and a 15 bp InDel were observed.

## Discussion

Lack of adequate quantities of vitamin A, iron and zinc in diet are main factors for micronutrient malnutrition. Biofortification of crop plants paved an economic and sustainable way to alleviate hidden hunger. Micronutrient deposition in grains are controlled by many genes and influenced by environmental conditions. In the process of biofortifying pearl millet with higher grain iron content, one open pollinated variety ICTP 8203Fe (Dhanshakti) and one hybrid ICMH 1201 (Shakti 1201) were released for cultivation by ICRISAT in collaboration with Harvest Plus, which had iron and zinc content of 71, 41 ppm and 75, 39 ppm, respectively (Rai et al., 2013) through conventional breeding. Further process is underway in this crop by many institutes to reach a target level of >77 ppm kept by Harvest Plus (http://www.harvestplus.org). This process will hasten up when QTLs related to grain iron and zinc content are identified and incorporated through marker-assisted breeding programs. Hence, the present study was attempted to decipher genomic regions for high grain iron and zinc content in pearl millet using association mapping.

Constitution of a panel with diverse lines is of prime requirement for association mapping (Flint-Garcia et al., 2005). The present association mapping panel comprised of 130 diverse lines of pearl millet with diverse pedigrees originating from different parts of India and Africa. The panel was phenotypically diverse with a wide range grain micronutrient content observed in different environments. Also, it was earlier reported that population consisting of <100 genotypes was treated as sub-optimal for association studies, in particularly with markers having low minor allele frequency (Zhu et al., 2008). However, some studies (Zhao et al., 2011) showed significant associations with a small panel of 57–97 genotypes. Hence, present size of the panel (130 genotypes) is considered substantial for association studies.

The average number of alleles per locus (2.65) detected in the present study is less than previous studies on pearl millet (Nepolean et al., 2012; Sehgal et al., 2015). According to Pasam et al. (2014), number of accessions taken, number and type of markers used for characterization play a role in detection of total number of alleles per locus. Same panel can result in varied detection of alleles depending upon the detection techniques (Gupta et al., 2010), for example, PAGE or capillary electrophoresis with DNA fragment analyzer are capable enough to resolve even two base pairs differences in the amplicons whereas 3.5% agarose gels used here do not have the same resolution. Hence, even though diverse breeding lines from different pedigrees were included in the present investigation and previously studied SSR markers were utilized, the difference in allele detection methodology used in this study from earlier studies led to this distinction. However, the number of alleles per loci detected here is more than detected by Tara et al. (2009) (2.0) in pearl millet, Gupta et al. (2012) (2.2), Pandey et al. (2013) (2.1) in foxtail millet. The present panel was moderately polymorphic according to Vaiman et al. (1994) since the average PIC value falls in the range of 0.25–0.5 values. The value obtained for average gene diversity (0.35) also indicates moderate diversity in the present lines chosen for study. This value is less than that of reported in earlier studies (Nepolean et al., 2012; Tara et al., 2013; Sehgal et al., 2015). Though the highest value of gene diversity (0.73) obtained here is similar to that reported by Mariac et al. (2006) (0.75) who characterized wild pearl millet genotypes for diversity studies, the average value in present study is lesser than that recorded by them (0.49 in cultivated and 0.67 in wild lines). This may be because of the differences in the genotypes and the allele detection methods used.

Before performing association analysis, genetic differentiation of the population was observed by STRUCTURE and compared with other clustering approaches. STRUCTURE, clustering analysis and PCoA showed the division of population into three sub-populations. All the three models exhibited similarity in the grouping pattern which is akin to earlier studies (Sehgal et al., 2015). But, there was no 100% match between clustering and STRUCTURE grouping pattern. Resemblance between two was more than 60%. In STRUCTURE grouping was based on highest percentage of membership of an individual to a sub-population and in cluster analysis each genotype is assigned to a fixed branch position (Gupta et al., 2014). Also, admixture was separated from groups and placed in separate group depending on their *Q-*value, but, in clustering those genotypes were also involved in sub groups leading to reduced similarity in the grouping pattern obtained by two different analysis. Totally, there was no structural pattern observed based on high and low grain iron and zinc content either in STRUCTURE or in clustering. Grouping pattern, was in accordance with their pedigree. These results were similar to those obtained earlier (Sehgal et al., 2015).

Genetic differentiation between sub populations was tested here using AMOVA, which presents general skeleton for the study of population structure (Michalakis and Excoffier, 1996). In this study, all pair wise F_ST_s were significantly different from one another, thus all sub-populations may be considered as significantly different from each other (Nachimuthu et al., 2015). Variation within sub-population was more compared to between sub-populations. Only 11% out of total variation was between sub-populations indicating lesser genetic structure of the population having free gene flow between sub-groups. This may be because of constant exchange of genetic material among breeding programs (Würschum et al., 2013). According to Wright (1978), when F_ST_ is between 0.05 and 0.15 values, it implies population differentiation is moderate. However, he defined it for biallelic markers or allozyme markers. Multi-allelic markers have low F_ST_ values compared to biallelic markers, because of inherent properties in calculation (Jakobsson et al., 2013). Since, the markers used here were multi-allelic, the value obtained (0.11) may indicate that genetic differentiation obtained here was moderate to high. Many diversity studies in pearl millet (Lewis, 2010; Nepolean et al., 2012), the maximum variation was contributed by within sub-population indicating that genotypes considered in this study were different from each other. In cross-pollinated species gene flow is likely to be greater within population than between population (Hamrick et al., 1990). We also observed some amount of within individual variance, which is indicative of presence of heterozygosity in the population. One reason is peri-centric regions have high degree of heterozygosity because of reduced recombination (McMullen et al., 2009 in Maize). In cross-pollinated crops like pearl millet, some amount of heterozygosity is expected. Nachimuthu et al. (2015) also observed some amount of individual variance in rice. The same reason i.e. cross-pollinated nature of pearl millet holds good for the low value of significant linkage disequilibrium (LD) obtained here. High values cannot be sustained because of frequent recombination as observed in maize (Zhu et al., 2008). LD is influenced by different factors like mode of reproduction, selection, rate of recombination, rate of mutation, genetic drift, and population structure. The low value of LD may ensure high resolution mapping, but more number of markers are needed to take this advantage (Gupta et al., 2005). Similarly, Zhang et al. (2013) observed only 2.95% locus pairs to be in significant LD in cotton crop. Broad-based population was reported to have low LD compared to narrow-based population. Hence the present panel may be broad-based, which supports the usefulness of panel for association mapping studies. A low LD demands more number of markers compared to high LD of same size population (Gupta et al., 2005), but there are some instances where less number of markers were quite enough to get MTAs (Li C. Q. et al., 2016). To capture the resolution in low LD crops, thousands of polymorphic markers are needed but it is possible only when those many markers are available, or when genome sequence is already known and all types of markers are easily predicted (Gupta et al., 2014).

Most widely used statistical models for marker-trait association studies are GLM (Pritchard et al., 2000) and MLM (Yu et al., 2006; Price et al., 2010). While GLM accounts for the population structure, MLM considers both population structure and familial relatedness. In GLM, there are chances of spurious associations (Type I error) because it only considers population structure and not kinship (Zhao et al., 2007). In MLM, sometimes, over compensation with both Q and K may lead to false negatives (Type II error; Zhao et al., 2007, 2011). This leads to detection of some MTAs only with a particular model (Liu et al., 2016). Hence, both models were tested for grain micronutrient association, but only the MTAs revealed by MLM are presented here, as MLM results were found to be more reliable.

The associations of the trait with markers were tested at three locations in the years 2014 and 2015. The data of individual environments (Del-14, Del-15, DW-14, DW-15, Jod-14, Jod-15) and their means pooled over environments in different combinations (Del-M, DW-M, Jod-M, Y14-M, Y15-M, and GM) were used to find significant associations of molecular markers with the individual trait. In this study, Bonferroni corrected *p*-value (*p* = 0.0037) was considered as a threshold for significance for declaring MTAs (Liu et al., 2016). Except for two markers (*Xipes* 0096 and *Xpsmp* 2261), there was inconsistency in marker-trait association by GLM and MLM which is expected and it may be because of the above mentioned Type I and Type II errors in one or the other models. These two loci which were detected by both approaches can be considered as the best MTAs. MLM analysis detected one SSR marker, *Xipes* 0180 to be associated consistently with grain iron content explaining more than 10% variation. It was also observed to be associated with grain zinc content at Jodhpur during 2015. A genic marker, *Xsinramp* 6, was also observed to be associated with both grain iron and zinc content. It recorded more than 9.5% *R*^2^-value for grain iron content at Delhi location, while for grain zinc content *R*^2^-value was more than 10.0% at Dharwad location.

A total of 16 MTAs for both grain iron and zinc content were identified in this study by using MLM analysis (Figure 5), out of which six MTAs were for grain iron and 10 for grain zinc content. *Xipes* 0180, belonging to LG3 was detected consistently for grain iron content. This marker was associated at Jod-15 (Jodhpur, 2015) for grain zinc content which is in accordance with the previous findings of bi-parental mapping, where LG2, LG3, LG5, and LG7 were reported to house QTLs for both iron and zinc content (Kumar et al., 2010, 2016). In present study, *Xpsmp* 2261 and *Xipes* 0096 belonging to LG5 and LG7, respectively, identified strong and consistent associations (not only with grain iron content, but also with grain zinc content in accordance with Kumar et al., 2010, 2016). However, in this study we did not find any MTA on LG2. Linkag group of five more markers (*Xicmp* 3004, *Xicmp* 3016, *Xicmp* 4006, *Xpsmp* 2209, and *Xsinramp* 6) associated with traits could not be ascertained. Hence, there is possibility that any of those five markers may map to LG2. Consistently identified markers namely, *Xpsmp* 2261 (LG5), *Xipes* 0096 (LG7), *Xipes* 0180 (LG3) and *Xsinramp* 6 (unmapped) were identified for both high grain iron and zinc content in the current study (Figure 5). Co-localization of high grain iron and zinc content alleles/QTLs in pearl millet has also been reported earlier by Kumar et al. (2016). This may be because, starting from uptake to final deposition into grain of iron and zinc may share some common pathways (Grotz and Guerinot, 2006).

This study identified 16 MTAs for grain iron and zinc content. Some were consistent across locations and years, while some were specific to certain locations or years. This may be because, these traits are governed by many genes and exhibit considerable genotype-by-environment (G × E) interactions. Some associations were specific to certain sub-populations and some though not observed at whole population level, were significant in more than one sub-population. Few associations were not detected at sub-population level but they were recorded when the whole population was analyzed. This variation is because of the structure present in the population. Even though few differences existed at sub-population levels, *Xpsmp* 2261, *Xipes* 0096, *Xipes* 0180, and *Xsinramp* 6 were consistently associated in all sub-populations. Different populations of varying sizes and structure also affect the detection of associations. Earlier workers (Zhao et al., 2007; Liu et al., 2016) reported different associations with different set of populations. Hence, true associations obtained here must be validated by testing in another panel having different genotypes.

Similarly, several genomic regions were detected for grain iron and zinc content in other crops (Upadhyaya et al., 2016 in chickpea, Nawaz et al., 2015 in rice) through association mapping. Likewise, many QTLs have been identified for other agronomically important traits in pearl millet through association mapping (Saïdou et al., 2014; Sehgal et al., 2015). Tadesse et al. (2015) also observed consistent and specifically adapted QTLs in wheat for grain quality traits. Hence, different genomic regions in this study can be introgressed for trait improvement in pearl millet based on the targeted environment depending upon common and location specific MTAs. *Xipes* 0180 amplicon sequence was found matching with a segment of pearl millet reference genome, and was annotated as aspartic proteinase (*Asp1*) gene. While *Asp1* may not have a direct role in grain iron and zinc metabolism, it might be indirectly involved through other gene networks and pathways in different metabolic pathways. In addition, the two putative SNPs and 15bp InDel (Figures 6A–C) identified in the present study do not seem to be associated with a particular trait. These may call for further investigations.

Mining down to the effect of alleles of significant MTAs will make us understand favorable alleles and the genotypes carrying them. In this regard, we calculated effect of an allele using population mean instead of null alleles (Cai et al., 2014; Liu et al., 2016) while, on the other hand, computation of effect of an allele with a null allele is still followed by many workers (Breseghello and Sorrells, 2006; Dang et al., 2016). We are of the opinion that mean is a representative value of the whole data, while missing alleles which are also included as null alleles are error prone due to differential handling of experiment will lead to different missing data which in turn changes the resultant effect of an allele. In many instances, it was noticed that average effect of an allele will be higher when the allele is rare (Cai et al., 2014). Stably expressing alleles linked to markers, *Xpsmp* 2261, *Xipes* 0180 *Xipes* 0096, and *Xsinramp* 6 recorded greater phenotypic effect which offer advantage for Fe and Zn enhancement and higher trait expression in the recipient lines. These linked alleles may be promising targets for marker-assisted selection (MAS). On the basis of occurrence of favorable alleles for high grain iron and zinc content in the panel, we have suggested a set of lines (Table 4) which can be used in the crossing programs to accumulate favorable alleles together in the segregating generations, resulting in higher Fe and Zn content. This is in line with other studies (Dang et al., 2016).

## Conclusion

The results of the current association mapping demonstrates usefulness of association mapping using SSRs and genic markers, and the current association mapping panel for identification of superior alleles for grain nutritional traits like iron and zinc content. The favorable alleles and the associated markers identified in the present study need to be validated in more diverse genetic backgrounds. Such validated markers might be useful in marker-assisted back-crossing (MABC), marker-assisted recurrent selection (MARS), and forward breeding programs. The promising lines with favorable alleles identified in this study may be used for generating new cultivars which accumulate all or most of the favorable alleles for high grain iron and zinc content.

## Author contributions

CS and RS planned and designed the research; NA, SMS, and SPS performed the field experiment; MM helped in Fe and Zn estimation; NA, CB, and TS conducted genotyping of the association mapping panel; NA and TN conducted STRUCTURE and TASSEL analysis; RS organized bioinformatics analysis; NA, CS, and RS prepared the manuscript. CS and RS edited the manuscript for publication. All authors have read and approved the final manuscript.

### Conflict of interest statement

The authors declare that the research was conducted in the absence of any commercial or financial relationships that could be construed as a potential conflict of interest.

## Supplementary material

Table S1 **List of pearl millet genotypes, their pedigree and inferred sub-population**.

Table S2 **List of 267 (250 SSRs and 17 genic) primer pairs used to characterize the association mapping panel**.

Table S3 **Summary statistics of 114 SSR markers used in the study**.

Table S4A **One way ANOVA for grain iron content (ppm) for six environments**.

Table S4B **One way ANOVA for grain zinc content (ppm) for six environments**.

Table S4C **Pooled ANOVA of years 2014 and 2015 for grain iron and zinc content for different locations**.

Table S4D **Pooled ANOVA across three locations for grain iron and zinc content for the years 2014 and 2015**.

Table S4E **Pooled ANOVA across 6 environments (2 years and three locations) for grain iron and zinc content**.

Table S5A **Mean values of grain iron content across six environments and their six pooled environments (ppm)**.

Table S5B **Mean values of grain zinc content across six environments and their six pooled environments (ppm)**.

Table S6 **Correlation between grain iron and zinc content and population structure using multiple regression analysis**.

Table S7A **Marker wise associations observed across all datasets for grain iron content in MLM at *p* < 0.0037**.

Table S7B **Marker wise associations observed across all datasets for grain zinc content in MLM at *p* < 0.0037**.

Table S8 **Linkage group wise number of significant markers associated with grain iron and zinc content**.

Table S9A **Markers associated significantly (*p* = 0.05) with grain iron content at sub-population level**.

Table S9B **Markers associated significantly (*p* = 0.05) with grain zinc content at sub-population level**.

Table S10A **Phenotypic effect of favorable alleles for grain iron content and top three genotypes carrying them**.

Table S10B **Phenotypic effect of favorable alleles for grain zinc content and top three genotypes carrying them**.

Table S11A **Top most genotypes carrying favorable alleles for grain iron content**.

Table S11B **Top most genotypes carrying favorable alleles for grain zinc content**.

Figure S1 **Quantile-Quantile (QQ) plots for grain iron content in MLM showing distribution of marker-trait association**.

Figure S2 **Quantile-Quantile (QQ) plots for grain zinc content in MLM showing distribution of marker-trait association**.
