# Supplementary material for: Deciphering Genomic Regions for High Grain Iron and Zinc Content Using Association Mapping in Pearl Millet
Source: Front Plant Sci. 2017 May 1;8:412. doi: 10.3389/fpls.2017.00412 (PMC5410614; doi:10.3389/fpls.2017.00412)
Supplement: Table S1 — List of pearl millet genotypes, their pedigree and inferred sub-population. [file Table1.DOCX]

**TABLE S1 │ List of pearl millet genotypes, their pedigree and inferred subpopulation**

| **Genotype code** | **Genotype** | **Pedigree** | **Sub Population** |
| --- | --- | --- | --- |
| 1 | PPMI 1102 | High Iron line selected from PPMI 683 x PPMI 627 | B |
| 2 | 5540B | Maintainer of 5540A developed through selection from Tift 23A at IARI | B |
| 3 | PPMI 214 | Restorer line with good combining ability developed at IARI from African germplasm | B |
| 4 | IPC 1657 | Germplasm collection belongs to ICRISAT pollinator collection collected at UP, India | Admix |
| 5 | PPMI 708 | Selection from downy mildew resistant West African lines | B |
| 6 | PPMI 1087 | Selection from ICRISAT dual purpose material, JBV 3 S1-95-3-3-2-B-B | B |
| 7 | PPMWGI 99 | White grain inbred developed at IARI, N. Delhi | B |
| 8 | J2467 | Restorer collection, male parent of GHB 757, belongs to ARS, Jamnagar | B |
| 9 | PPMI 1225 | Promising early maturing restorer selected from mapping population, 843B X 841B | B |
| 10 | J2405 | Restorer collection, male parent of GHB 577, belongs to ARS, Jamnagar | B |
| 11 | PPMFeZMP 199 | Inbred line selected with good agronomic score from Fe and Zn mapping population, PPMI 683 X PPMI 627 | B |
| 12 | PPMFeZMP 65 | Inbred line selected with good agronomic score from Fe and Zn mapping population, PPMI 683 X PPMI 627 | B |
| 13 | ICTP 8203 Fe | High Fe selection from ICTP 8203 which was produced through random mating of five S_2_ Iniari landrace originating from N.Togo | B |
| 14 | PPMI 1222 | Promising early maturing restorer selected from mapping population, 843B X 841B | B |
| 15 | PPMI 683 | High Iron restorer line developed at IARI, New Delhi | Admix |
| 16 | PPMI 627 | Promising medium maturity restorer developed at IARI, New Delhi | B |
| 17 | ICMR06222 | SDMV 90031-S1-3-3-2-1-3-2-2-1-B | B |
| 18 | PPMI 660 | Promising medium maturity restorer developed at IARI, New Delhi with good combining ability | B |
| 19 | PIB 228 | Restorer, male parent of PHB 14(HB 7) developed by PAU | B |
| 20 | PPMWGI 146 | White grain inbred developed at IARI, N. Delhi | B |
| 21 | PPMI 295 | Inbred developed from mutant progeny of Bil 3B | B |
| 22 | PPMI 1220 | Promising early maturing restorer selected from mapping population, 843B X 841B | B |
| 23 | PPMFeZMP 37 | Inbred line selected with good agronomic score from Fe and Zn mapping population, PPMI 683 X PPMI 627 | B |
| 24 | J108 | Restorer collection, male parent of GHB15, belongs to ARS, Jamnagar | B |
| 25 | PPMI 85 | inbred developed from mutant progeny of Bil 3B | A |
| 26 | PPMI 301 | Elite restorer, male parent of Pusa 322, Pusa 444, developed by IARI, N.Delhi | A |
| 27 | ICMB 98222 | ARD-288-1-10-1-2 (RM)-5 | A |
| 28 | D 23 | Downy Mildew resistant selection from K 560-230 | A |
| 29 | PPMI 1103 | High Iron line selected from PPMI 683 x PPMI 627 | A |
| 30 | PPMI 1104 | High Iron line selected from PPMI 683 x PPMI 627 | A |
| 31 | PPMI 1105 | High Iron line selected from PPMI 683 x PPMI 627 | Admix |
| 32 | PPMI 1107 | High Iron line selected from PPMI 683 x PPMI 627 | Admix |
| 33 | PPMI 1108 | High Iron line selected from PPMI 683 x PPMI 627 | C |
| 34 | PPMI 1112 | High Iron line selected from PPMI 683 x PPMI 627 | A |
| 35 | 841B | Downy Mildew resistant selection from residual variability available in seed lot number 8015 of 5141B | A |
| 36 | 5141B | Cross derivative of Tift 23 B X IP 1587 (Baroda-4) | A |
| 37 | 5054B | Cross derivative of Tift 23 B X Kano-2457 (Nigeria), good for drought prone area | Admix |
| 38 | 6030B | Male sterile line developed at IARI | A |
| 39 | 351B | Male sterile line developed at IARI | A |
| 40 | 379B | Male sterile line developed at IARI | A |
| 41 | 411B | Early flowering line developed as selection from 263 B | A |
| 42 | 576B | Maintainer of 576A (Cross derivative of 5141B X P 7) | Admix |
| 43 | PPMI 59 | Promising good combiner inbred restorer developed at IARI from African germplasm | A |
| 44 | PPMI 265 | Promising good combiner inbred restorer developed at IARI from African germplasm | Admix |
| 45 | PPMI 269 | Promising good combiner inbred restorer developed at IARI from African germplasm | A |
| 46 | PPMI 275 | Inbred developed from mutant progeny of Bil 3B | Admix |
| 47 | PPMI 496 | Restorer with good combining ability selected from PPMI 69 | A |
| 48 | PPMI 694 | Restorer with good combining ability selected from PPMI 85 | A |
| 49 | PPMI 719 | P 309-85-3-2-2-6-2-2 | Admix |
| 50 | PPMI 744 | Restorer with good combining ability selected from PPMI 85 | Admix |
| 51 | PPMI 759 | P 671 S2-54-4-3-2-1-1 | Admix |
| 52 | EGPN 423 | White grain inbred selected from Etiopian material | C |
| 53 | PPMWGI 152 | White grain inbred developed at IARI, N. Delhi | C |
| 54 | PPMWGI 100 | White grain inbred developed at IARI, N. Delhi | C |
| 55 | PPMWGI 108 | White grain inbred developed at IARI, N. Delhi | C |
| 56 | KSMWGI 14 | White grain inbred developed at IARI, N. Delhi by KS Mangath | C |
| 57 | PPMI 1224 | Promising early maturing restorer selected from mapping population, 843B X 841B | C |
| 58 | PPMI 1231 | Promising early maturing restorer selected from mapping population, 843B X 841B | Admix |
| 59 | PPMI 1233 | Promising early maturing restorer selected from mapping population, 843B X 841B | Admix |
| 60 | PPMI 1263 | Promising early maturing restorer selected from mapping population, 843B X 841B | Admix |
| 61 | ICMR07111 | MRC HS-41-2-2-3-B-B-P1-B-B-B | Admix |
| 62 | ICMR07999 | (ICMV-IS 94206-7 × (SRC II C3 S1-1-1-2 x HHVBC)-1-3-3))-B-10-1-2-2 | Admix |
| 63 | PPMI 1084 | Dual purpose material selection from JBV 3 S1-6-1-1-4-1-3 | Admix |
| 64 | PPMI 1086 | Dual purpose material selection from ICMV 91059 S1-58-4-3-3-1-B-B-B-B-B-B | C |
| 65 | PPMI 1089 | Dual purpose material selection from (ICMS 7704-S1-127-5-1 X RCB-2 Tall)-B-19-3-2-1-1 | Admix |
| 66 | PPMI 1090 | Dual purpose material selection from [(((ICMV-IS 94206-15)X B-Lines)-B-6) X (MRC S1-156-2-1-B]-B-22-1-3-1 | Admix |
| 67 | PPMI 1092 | Dual purpose material selection from ICRISAT material | A |
| 68 | PPMI 1155 | Thick panicle restorer selected from AIMP 92901 S1-272-2-3-3-1-B-B-B | Admix |
| 69 | PPMI 1161 | Thick panicle restorer selected from MC 94 C2-S1-3-1-1-2-4-B-B-3 | Admix |
| 70 | PPMI 1165 | Thermo tolerant line selected from lot number 868, CAZRI, Jodhpur | A |
| 71 | PIB 686 | Restorer, male parent of PHB 2168 developed by PAU | Admix |
| 72 | HBL 11 | Male parent of hybrid HHB 197 developed by CCSHAU | Admix |
| 73 | H77/833-2-202 | Male parent of HHB 67 (Improved) developed by backcrossing ICMP 451 with H77/833-2 as recurrent parent through MAS | Admix |
| 74 | H77/833-2 | Male parent of HHB 67 | Admix |
| 75 | J2454 | Restorer collection belongs to ARS, Jamnagar | Admix |
| 76 | J2496 | Restorer collection belongs to ARS, Jamnagar | C |
| 77 | IPC 1480 | ICRISAT pollinator collection | C |
| 78 | PPMFeZMP 72 | Inbred line selected with good agronomic score from Fe and Zn mapping population, PPMI 683 X PPMI 627 | C |
| 79 | PPMI 1218 | Promising early maturing restorer selected from mapping population, 843B X 841B | C |
| 80 | PPMI 1267 | Inbred line selected with good agronomic score from Fe and Zn mapping population, PPMI 683 X PPMI 627 | C |
| 81 | PPMI 1268 | Inbred line selected with good agronomic score from Fe and Zn mapping population, PPMI 683 X PPMI 627 | C |
| 82 | PPMI 1269 | Inbred line selected with good agronomic score from Fe and Zn mapping population, PPMI 683 X PPMI 627 | C |
| 83 | PPMFeZMP 34 | Inbred line selected with good agronomic score from Fe and Zn mapping population, PPMI 683 X PPMI 627 | C |
| 84 | PPMFeZMP 125 | Inbred line selected with good agronomic score from Fe and Zn mapping population, PPMI 683 X PPMI 627 | C |
| 85 | PPMFeZMP 126 | Inbred line selected with good agronomic score from Fe and Zn mapping population, PPMI 683 X PPMI 627 | C |
| 86 | PPMFeZMP 153 | Inbred line selected with good agronomic score from Fe and Zn mapping population, PPMI 683 X PPMI 627 | C |
| 87 | HTP 94/54 | Developed by selecting selfed progenies of high tillering Togo population | C |
| 88 | PPMI 1270 | Inbred line selected with good agronomic score from Fe and Zn mapping population, PPMI 683 X PPMI 627 | C |
| 89 | PPMI 1271 | Inbred line selected with good agronomic score from Fe and Zn mapping population, PPMI 683 X PPMI 627 | C |
| 90 | PPMI 1272 | Inbred line selected with good agronomic score from Fe and Zn mapping population, PPMI 683 X PPMI 627 | C |
| 91 | PPMFeZMP 30 | Inbred line selected with good agronomic score from Fe and Zn mapping population, PPMI 683 X PPMI 627 | C |
| 92 | PPMFeZMP 35 | Inbred line selected with good agronomic score from Fe and Zn mapping population, PPMI 683 X PPMI 627 | C |
| 93 | PPMFeZMP 47 | Inbred line selected with good agronomic score from Fe and Zn mapping population, PPMI 683 X PPMI 627 | C |
| 94 | PPMFeZMP 87 | Inbred line selected with good agronomic score from Fe and Zn mapping population, PPMI 683 X PPMI 627 | C |
| 95 | PPMI 1273 | Inbred line selected with good agronomic score from Fe and Zn mapping population, PPMI 683 X PPMI 627 | C |
| 96 | PPMI 1274 | Inbred line selected with good agronomic score from Fe and Zn mapping population, PPMI 683 X PPMI 627 | Admix |
| 97 | PPMFeZMP 143 | Inbred line selected with good agronomic score from Fe and Zn mapping population, PPMI 683 X PPMI 627 | A |
| 98 | G73-107 | Restorer line, male parent of HHB 94 developed by CCSHAU | A |
| 99 | PPMI 1067 | Selection from Downy mildew mapping population | A |
| 100 | PPMI 1276 | White grain inbred line selected from WGI 148 X WGI 52 | A |
| 101 | PPMDMGMP 27 | White grain inbred line selected from WGI 148 X WGI 52 | A |
| 102 | PPMDMGMP 60 | White grain inbred line selected from WGI 148 X WGI 52 | A |
| 103 | PPMDMGMP 87 | White grain inbred line selected from WGI 148 X WGI 52 | A |
| 104 | PPMDMGMP 99 | White grain inbred line selected from WGI 148 X WGI 52 | A |
| 105 | PPMI 1277 | White grain inbred line selected from WGI 148 X WGI 52 | A |
| 106 | PPMI 1278 | White grain inbred line selected from WGI 148 X WGI 52 | A |
| 107 | PPMI 1011 | Selection from A 5 restorer | A |
| 108 | ICMB 04222 | 843B x EEBC S1-407)-12-3-B | A |
| 109 | PPMI 1279 | White grain inbred line selected from WGI 148 X WGI 52 | A |
| 110 | PPMDMGMP 236 | White grain inbred line selected from WGI 148 X WGI 52 | A |
| 111 | PPMDMDMP 86 | White grain inbred line selected from WGI 52 X WGI 148 | A |
| 112 | PPMI 1116 | High Iron line selected from PPMI 683 x PPMI 627 | A |
| 113 | PPMI 1280 | White grain inbred line selected from WGI 52 X WGI 148 | A |
| 114 | PPMI 1281 | White grain inbred line selected from WGI 52 X WGI 148 | A |
| 115 | ICMB 92777 | [843B × (ICMPS 500-4-4-3 × ICMPS 1800-3-1-2-C3-4)]-7-1-3 | A |
| 116 | PPMI 1282 | Selection from PI 279663, collected from India | A |
| 117 | PPMI 1283 | Selection from PI 295158, collected from Zimbabwe | A |
| 118 | PPMI 1284 | Selection from PI 338000, collected from Ethiopia | A |
| 119 | PPMI 1101 | Selection from High Head Volume B Composite Tall-17 | A |
| 120 | PPMI 1286 | Selection from PI 526283, collected from Zimbabwe | A |
| 121 | PPMI 823 | Derivative of cross involving a West African line and an Indian line | A |
| 122 | PPMI 1275 | White grain inbred line selected from WGI 148 X WGI 52 | A |
| 123 | PPMDMGMP 8 | White grain inbred line selected from WGI 148 X WGI 52 | A |
| 124 | PPMDMGMP 148 | White grain inbred line selected from WGI 148 X WGI 52 | C |
| 125 | PPMDMGMP 186 | White grain inbred line selected from WGI 148 X WGI 52 | C |
| 126 | PPMDMDMP 93 | White grain inbred line selected from WGI 52 X WGI 148 | C |
| 127 | PPMDMDMP 107 | White grain inbred line selected from WGI 52 X WGI 148 | A |
| 128 | PPMI 1285 | Selection from PI 521638, collected from Kenya | A |
| 129 | PPMI 1287 | Selection from PI 526308, collected from Zimbabwe | Admix |
| 130 | PPMFeZMP 22 | Inbred line selected with good agronomic score from Fe and Zn mapping population, PPMI 683 X PPMI 627 | Admix |
