# Supplementary material for: Deciphering Genomic Regions for High Grain Iron and Zinc Content Using Association Mapping in Pearl Millet
Source: Front Plant Sci. 2017 May 1;8:412. doi: 10.3389/fpls.2017.00412 (PMC5410614; doi:10.3389/fpls.2017.00412)
Supplement: Table S2 — List of 267 (250 SSRs and 17 genic) primer pairs used to characterize the association mapping panel. [file Table2.docx]

**TABLE S2 │List of 267(250 SSRs and 17 genic) primer pairs used to characterize the association mapping panel.**

| **S.No.** | **Primer name** | **Forward primer_sequence** | **Reverse primer_sequence** | **Annealing temperature (°C)** | **Source** |
| --- | --- | --- | --- | --- | --- |
| 1 | CTM1 | TCTGGGGATTGGCTGGAATTACA | AAGTTGGGTAACGCCAGGGTTTTC | 61.6 | Budak et al. 2003 |
| 2 | CTM10 | GAGGCAAAAGTGGAAGACAG | TTGATTCCCGGTTCTATCGA | 52.7 | Budak et al. 2003 |
| 3 | CTM11 | GACCGATCTTCTTTGCTGTTG | TCTATCGTACGTTAACCTCA | 49.1 | Budak et al. 2003 |
| 4 | CTM12 | GTTGCAAGCAGGAGTAGATCGA | CGCTCTGTAGGTTGAACTCCTT | 55.0 | Budak et al. 2003 |
| 5 | CTM2 | GGTGATTAAAATCGAGGGTT | AGCAACTTGAGCAGCGG | 50.7 | Budak et al. 2003 |
| 6 | CTM21 | ATGCCTCCCACCCCACGTCG | CGTCGCACTAGCCACAGTCA | 61.3 | Budak et al. 2003 |
| 7 | CTM25 | GCGAAGTAGAACACCGCGCT | GCACTTCCTCCTCGCCGTCA | 60.5 | Budak et al. 2003 |
| 8 | CTM26 | GCAAGTGATCCATGACATTACGA | GCGAAGTAGAACACCGCGCT | 57.6 | Budak et al. 2003 |
| 9 | CTM27 | GTTGCAAGCAGGAGTAGATCGA | CGCTCTGTAGGTTGAACTCCTT | 55.0 | Budak et al. 2003 |
| 10 | CTM3 | AGCAACTTGAGCAGCGG | GTCCATCGTCGCCGACGAA | 56.5 | Budak et al. 2003 |
| 11 | CTM55 | CGTCTTCTACCACGTCCT | CATAATCCCACTCAACAATCC | 54.0 | Budak et al. 2003 |
| 12 | CTM56 | GCGTTGTTTCGGTGACCAC | GCGTATCTTTAAATTGCCTTTGTT | 55.4 | Budak et al. 2003 |
| 13 | CTM57 | TGGTGGCAATGCAAGCTACAG | AGCGAGACGATCGACAGGG | 57.8 | Budak et al. 2003 |
| 14 | CTM58 | TACGTGCTACAAGAATGG | GCTGGCTAGGACACAA | 43.2 | Budak et al. 2003 |
| 15 | CTM59 | TCCTCGACATCCTCCA | GACACCTCGTAGCACTCC | 54.0 | Budaket al., 2003 |
| 16 | CTM60 | AAGCCCCGATCACATCAA | AGCCGAGCCTCATCCC | 52.9 | Budak et al. 2003 |
| 17 | CTM8 | GCTGCATCGGAGATAGGGAA | CTCAGCAAGCACGCTGCTCT | 57.2 | Budak et al. 2003 |
| 18 | CTM9 | GCCTCCTCTTGATACCATATT | TAGCCTTGGCTGCTATATTC | 49.0 | Budak et al. 2003 |
| 19 | CUMP007 | GAGGGATTCCAGGCGGTTC | GCGAGGAGCACATTCGATGAA | 58.7 | Yadav et al*.* 2007 |
| 20 | CUMP008 | GTTGACTACCACTATTATGCTCC | GACCAAGAACTTCATACAATTCAG | 50.2 | Yadav et al*.* 2007 |
| 21 | CUMP009 | ATCTGATCGTGAGGCCTCAAC | GCCGACCAAGAACTTCATACAAT | 55.6 | Yadav et al*.* 2007 |
| 22 | CUMP010 | GCTGAACTATTCTGTAAACTTAAC | TATCGAAACGGTACTAAAATCATG | 49.2 | Yadav et al*.* 2007 |
| 23 | ICMP3002 | AAGATGGATGATGGATTGATGA | TACACACACATTGCCACACG | 53.1 | Senthilvel et al., 2004 |
| 24 | ICMP3004 | TGTTACGCAGTGCTCGGTAG | ATATAGGGGCGCGCAATAGT | 54.4 | Senthilvel et al., 2004 |
| 25 | ICMP3005 | CGCGGTGTTCTCACACAC | TGTGAATTCCGCGGGTATAG | 53.6 | Senthilvel et al., 2004 |
| 26 | ICMP3006 | AAATCGGTCGTGGTGAAGTT | GAGAATGTGGGAGACACACG | 52.7 | Senthilvel et al., 2004 |
| **S.No.** | **Primer name** | **Forward primer_sequence** | **Reverse primer_sequence** | **Annealing temperature (°C)** | **Source** |
| 27 | ICMP3008 | GCACGAGGGTTGATTAGGC | CTCAATAAGAGGGGCGAGAA | 53.8 | Senthilvel et al., 2004 |
| 28 | ICMP3010 | TGTCTCGAGAGCAGGTGATG | AGAATGTGGGGGAGACACAC | 53.2 | Senthilvel et al., 2004 |
| 29 | ICMP3013 | TGTGGGAGAGAGGAGAGTCC | GCGCATATATGTGGGTGTGT | 52.7 | Senthilvel et al., 2004 |
| 30 | ICMP3014 | TGCTTCACAGCCTCTCCATA | CCACCATGCAACAGCAATAA | 53.5 | Senthilvel et al., 2004 |
| 31 | ICMP3016 | GTCAACCATTTGGGCTCACT | GGGAGAAATGTGGGGAGAGA | 54.3 | Senthilvel et al., 2004 |
| 32 | ICMP3017 | CACCAAACAGCATCAAGCAG | AGGTAGCCGAGGAAGGTGAG | 54.2 | Senthilvel et al., 2004 |
| 33 | ICMP3018 | ACGAGGACAAGCTCTTGGAA | ACGGCGCATACTCGATCATA | 54.5 | Senthilvel et al., 2004 |
| 34 | ICMP3024 | ATCGAGGCCAAGTACGTGAT | ATCGAACTGCACGTTAGCAA | 53.2 | Senthilvel et al., 2004 |
| 35 | ICMP3025 | GTTGCAGATGAGCGATCGTA | CGCCGACCAAGAACTTCATA | 54.2 | Senthilvel et al., 2004 |
| 36 | ICMP3027 | ACACCATCACCGACAACAAA | AGTGACCTGGGGTACAGACG | 53.3 | Senthilvel et al., 2004 |
| 37 | ICMP3028 | ACGATTCTTCGTCGTTCCAG | GATACGCGCGAGCTACATTT | 54.2 | Senthilvel et al., 2004 |
| 38 | ICMP3029 | ATCGATCTGTTCCACCCAGT | GGACTGGTACTGCTGCTGCT | 53.5 | Senthilvel et al., 2004 |
| 39 | ICMP3032 | AGGTAGCCGAGGAAGGTGAG | CAACAGCATCAAGCAGGAGA | 54.0 | Senthilvel et al., 2004 |
| 40 | ICMP3037 | CGTCGCTGCTCTTTCTTCTT | ATTTCAGAAACGGCAACCAA | 54.3 | Senthilvel et al., 2004 |
| 41 | ICMP3038 | CTCTCGGTTTGACGGTTTGT | GGGGAAAACAAAGTTGCTCA | 53.9 | Senthilvel et al., 2008 |
| 42 | ICMP3039 | GGCACGAGGGGCTAAGTAA | GGAACGCCGAGTACACAGAT | 53.9 | Senthilvel et al., 2008 |
| 43 | ICMP3042 | TAGTTAATGGGGGTGCGTGT | AAGCACCATCAGCATACCC | 53.0 | Senthilvel et al., 2008 |
| 44 | ICMP3043 | TCCTGTACAAGGACGTGCAG | TATCGACGCCAACGATACTG | 53.3 | Senthilvel et al., 2008 |
| 45 | ICMP3045 | ACAAGGACGACAAGGACCAC | CCTCTCCAAGCACATGTTTC | 52.6 | Senthilvel et al., 2008 |
| 46 | ICMP3047 | CGGAGACGCACTAGACTTGG | ACCACCATTCCATCACTCCT | 53.6 | Senthilvel et al., 2008 |
| 47 | ICMP3048 | CGGAACTGCTGGAGTGAAAT | GCGACTTCGACCGACTTTT | 54.0 | Senthilvel et al., 2008 |
| 48 | ICMP3049 | GAGCTGAACACGCTCAAGG | CAGATGACATCCATCCGTTG | 53.0 | Senthilvel et al., 2008 |
| 49 | ICMP3050 | ATGTCCAGTGTTGACGGTGA | CGGGGAAGAGACAGGCTACT | 53.3 | Senthilvel et al., 2008 |
| 50 | ICMP3051 | TCTTCTTCCGCATCCTCTGT | GTACCGCCCTTTGTGTTGAT | 53.6 | Senthilvel et al., 2008 |
| 51 | ICMP3055 | CCCAAACGCAAGTAGGGTTA | CCTTCTCCTGCCCCAGAC | 54.0 | Senthilvel et al., 2008 |
| 52 | ICMP3056 | ACGGAGCTACGGTTGGAATA | CACAAGGGACCCCACGATA | 54.4 | Senthilvel et al., 2008 |
| **S.No.** | **Primer name** | **Forward primer_sequence** | **Reverse primer_sequence** | **Annealing temperature (°C)** | **Source** |
| 53 | ICMP3057 | ATGTGGAATAACCGCAGAGG | AGCAAAAGCTGAGCGACTTC | 53.7 | Senthilvel et al., 2008 |
| 54 | ICMP3058 | CGGAGCTCCTATCATTCCAA | GCAAGCCACAAGCCTATCTC | 53.9 | Senthilvel et al., 2008 |
| 55 | ICMP3063 | TCCGGTAGAGACCGTAATGG | GGCACTCCCTAGCAAAATGA | 53.9 | Senthilvel et al., 2008 |
| 56 | ICMP3066 | GGCCCCAAGTAACTTCCCTA | TGTCAGACACAGATGCCACA | 53.5 | Senthilvel et al., 2008 |
| 57 | ICMP3068 | CTGGCAAAGTTGTAGCGTGA | ATGTCGCTCTCTGCCAAGAT | 53.6 | Senthilvel et al., 2008 |
| 58 | ICMP3069 | TAGGAGGGGACTGCTCCTTT | AGGAAGAGGATGGTGGTGTG | 53.7 | Senthilvel et al., 2008 |
| 59 | ICMP3072 | CGCAGCTCTACTTCCAGACC | CAGTAACCAAGAGCACCTCGAT | 54.3 | Senthilvel et al., 2008 |
| 60 | ICMP3076 | CACGAGGCAGAAGCACATT | CTCTTCTCGGCGATGAGC | 53.1 | Senthilvel et al., 2008 |
| 61 | ICMP3077 | AGCATCCCCTACACCATCAG | CTCTTCTCTCGCACACATGC | 53.3 | Senthilvel et al., 2008 |
| 62 | ICMP3078 | TCCAGACAGTTCAGCAGGTG | CCACACGAGACAGAGCACAC | 53.3 | Senthilvel et al., 2008 |
| 63 | ICMP3079 | ATGGTAGAGCGGTGAGGTTG | GCAAGGCAATGTAGGTGGAT | 53.7 | Senthilvel et al., 2008 |
| 64 | ICMP3080 | CAAACAGCATCAAGCAGGAG | GCGTAGACGGCGTAGATGAT | 53.6 | Senthilvel et al., 2008 |
| 65 | ICMP3081 | ACGCCGTTTTCGTGTAGTCT | TCCACAAGGTGACCTCACTG | 53.2 | Senthilvel et al., 2008 |
| 66 | ICMP3085 | CTGAAGCTGAAGAGGCCTTG | GGCGGAGATCAGAGTTCG | 53.2 | Senthilvel et al., 2008 |
| 67 | ICMP3086 | ACCAAACGTCCAAACCAGAG | ATATCTCTTCGCTGCGGTGT | 53.7 | Senthilvel et al., 2008 |
| 68 | ICMP3088 | TCAGGTGGAGATCGATGTTG | TTACGGGAGGATGAGGATG | 52.3 | Senthilvel et al., 2008 |
| 69 | ICMP3091 | AACAAGGACCTGCGATTCAC | CATGACAGCAACGACGAATC | 53.6 | Senthilvel et al., 2008 |
| 70 | ICMP3092 | GTTGCTGTCATGTCGTCTGG | CATCATGCCTGTGAGCAATG | 54.0 | Senthilvel et al., 2008 |
| 71 | ICMP3093 | AGTTTCCAATCCCACCCTCT | GTTGGAGATGAGGTCGAGGT | 53.1 | Senthilvel et al., 2008 |
| 72 | ICMP3094 | GACCTCGACCTCATCTCCAA | CGACAGCGAACTGGGATTC | 54.3 | Senthilvel et al., 2008 |
| 73 | ICMP3095 | GGGAGGCCACGATTTAAAGA | ACAATGTGCACGCAAGGA | 54.0 | Senthilvel et al., 2008 |
| 74 | ICMP3096 | CTGCATTGCAACATCCTCAC | AACCTGCAGTGGAAGCAATC | 53.7 | Senthilvel et al., 2008 |
| 75 | ICMP4006 | TGAGGACCGAGAAGAAGCAT | CAACACCCAACAGAAACTGAA | 52.9 | Senthilvel et al., 2008 |
| 76 | ICMP4007 | ATGTCCATTGCATCTCCGTA | TTGGCGATATCCTAAAAATGG | 52.9 | Senthilvel et al., 2008 |
| 77 | ICMP4014 | TTCCTTCAATACACAGTTGTTGG | ACCATGAGGACCTTGACCAG | 53.3 | Senthilvel et al., 2008 |
| 78 | IPES0003 | GTTCAGATGAACAGCGGGAT | AGTCCTCGGCAAGCCTTTAT | 60.2 | Rajaram et al., 2013 |
| **S.No.** | **Primer name** | **Forward primer_sequence** | **Reverse primer_sequence** | **Annealing temperature (°C)** | **Source** |
| 79 | IPES0004 | GTGCGTTCTTCCTTGCCTAC | TCATCACACAGGGCTAGCTG | 60.0 | Rajaram et al., 2013 |
| 80 | IPES0007 | ACACCTCGCTGCACCTCTA | GCAACACAGATGAGACTGGC | 59.4 | Rajaram et al., 2013 |
| 81 | IPES0009 | TTGATCGATCGTCTACGGTT | TATACTCACTCACGGCAGCG | 60.0 | Rajaram et al., 2013 |
| 82 | IPES0011 | TGGAGAAAGGGAAGCTCAGA | TGCTGCATCATCAACCCTTA | 60.2 | Rajaram et al., 2013 |
| 83 | IPES0015 | ATAACATGGCAACGCCTACC | CGAGGACGCAATAACACAGA | 59.9 | Rajaram et al., 2013 |
| 84 | IPES0017 | CCTATGGCGGCAGAGTAGTG | TTCCGGCACAATTACTTTCA | 59.2 | Rajaram et al., 2013 |
| 85 | IPES0019 | ATTGCTCTTCCAACGAGGTG | TGCTATAGGCAGACTTTGAGAAA | 58.4 | Rajaram et al., 2013 |
| 86 | IPES0023 | CACGACGTTGTAAAACGACGAGCCTTTCCAGACTTCACG | CTTTAATCGGGCACCACCTA | 65.6 | Rajaram et al., 2013 |
| 87 | IPES0027 | TGCTTGGGACAAAAGGCT | TAACTCAAGTGAGCGCAAGG | 59.2 | Rajaram et al., 2013 |
| 88 | IPES0035 | TGTTGGAAACAAAACCCGAT | ATTACCACGTCTACCTGCCG | 60.0 | Rajaram et al., 2013 |
| 89 | IPES0042 | GATAGAAGCAGATGGGCCTG | CTCGTCATCATTCTCGCCAC | 61.8 | Rajaram et al., 2013 |
| 90 | IPES0045 | CAGCACCATTAGTGGCAAAA | CGTAACTTTGGTCAGGCATACA | 60.1 | Rajaram et al., 2013 |
| 91 | IPES0052 | GGATCATCGATCAGCCGAC | CATGGTGATCTGAATCGCAG | 60.2 | Rajaram et al., 2013 |
| 92 | IPES0066 | CAACATGTCAAGGAAGTAAAATTGA | GCCTCTTGATACCCAAGATCA | 59.1 | Rajaram et al., 2013 |
| 93 | IPES0071 | CGATGCATGTATGTATGAATGA | GAAAAGTTCTTTCCTCCCCC | 59.0 | Rajaram et al., 2013 |
| 94 | IPES0076 | ATCCTTAGCAGGACAAGCGA | CCAAGCTTTTGGTTCAGGAG | 59.8 | Rajaram et al., 2013 |
| 95 | IPES0079 | GTTGGACAGGCGAACGATAC | AGCTCTCCTGCATTTTCGTG | 60.5 | Rajaram et al., 2013 |
| 96 | IPES0082 | CGACCCCTGAAGGAAATCTT | TTCTTCATGTGGGTGTCGAA | 60.1 | Rajaram et al., 2013 |
| 97 | IPES0085 | CATCGCGAAGAAGCTCAAG | AGACACCAGAGGCAGAGCAT | 60.0 | Rajaram et al., 2013 |
| 98 | IPES0087 | AAGGATGAGGACAACGATGC | AGCCAGCCCAATAAGCTACA | 59.9 | Rajaram et al., 2013 |
| 99 | IPES0089 | GACCTCAAGAAAATCGAGCG | ACAGTAGGTTTTGCGATGGG | 60.0 | Rajaram et al., 2013 |
| 100 | IPES0093 | GGATCTGCAGGTTTGGACAT | CCAAGCACTGAAACATGCAC | 60.3 | Rajaram et al., 2013 |
| 101 | IPES0095 | GTCTTTGCCGTGATAACCGT | CAAGAGGACTGGCTGATTGC | 60.9 | Rajaram et al., 2013 |
| 102 | IPES0096 | GCACTTTGTCACCGCTTCTT | GCAGCATGCCCTTTTCTATT | 59.3 | Rajaram et al., 2013 |
| 103 | IPES0097 | CGGGGACTGCTTGTAATTGT | ATGACGATGGCCTACGAAAC | 60.0 | Rajaram et al., 2013 |
| 104 | IPES0098 | ATCAAGCTTCATACCCCTGC | CATCTTCTTCATCATCTTTCGC | 58.9 | Rajaram et al., 2013 |
| **S.No.** | **Primer name** | **Forward primer_sequence** | **Reverse primer_sequence** | **Annealing temperature (°C)** | **Source** |
| 105 | IPES0101 | CCTGGAAGGAGGGAGAACAG | GATAGCCCAAAGGCAACAAA | 60.1 | Rajaram et al., 2013 |
| 106 | IPES0102 | ACATGTGTTGGCTTGCTGTG | CATCCTGTCCTGTCGTGCTA | 59.9 | Rajaram et al., 2013 |
| 107 | IPES0103 | CATGCCAAATCATCTCGATCT | CTGAACCCGGAATTGCATAC | 60.3 | Rajaram et al., 2013 |
| 108 | IPES0105 | GGGGGCTCACAGAACAAGTA | CCGAAGTTCCCACAGAATGT | 60.0 | Rajaram et al., 2013 |
| 109 | IPES0109 | ATTGCATTGGCCTTCTTCAG | GTCCAGTGGTCGTTGGGTAT | 59.7 | Rajaram et al., 2013 |
| 110 | IPES0114 | CGTTGTGTTGAATAATGTCGTACC | CAATAACCAAACGACGGACA | 59.4 | Rajaram et al., 2013 |
| 111 | IPES0117 | TTATTATTCGGTCATCACAGCG | TCCAAAACACAATTCCACCC | 60.6 | Rajaram et al., 2013 |
| 112 | IPES0118 | AAGGTGCAGAAGTTCACGCT | TTTTACAATCACGGCACGAC | 59.6 | Rajaram et al., 2013 |
| 113 | IPES0123 | CAAAATTTGGAACCACTGGG | TATGCTTCTGCTGTTCGGTG | 60.0 | Rajaram et al., 2013 |
| 114 | IPES0126 | CCAGCAGGGAAGTCTTTCAC | AAAGGCGCTTGCTGATTTT | 60.0 | Rajaram et al., 2013 |
| 115 | IPES0127 | TGTACAAATGATACTTGATATCCCAAA | TGCAGAATTACACTGCCCTG | 59.9 | Rajaram et al., 2013 |
| 116 | IPES0129 | GACCATGATTCGATTCTGCAT | CCTTTGTCCATCTCGTTTGA | 58.7 | Rajaram et al., 2013 |
| 117 | IPES0139 | GTGTATGGTATGCGTGTCCG | GCATTGTGATCGAATAAACTACTG | 57.9 | Rajaram et al., 2013 |
| 118 | IPES0141 | GCACACTGTATGTCTAGCTGGTG | GTCCAGTGGTCGTTGGGTAT | 59.7 | Rajaram et al., 2013 |
| 119 | IPES0142 | GATTTGTTAATGTTGGTGTTGAGC | GCATGCTGCTGAACTATGGA | 60.0 | Rajaram et al., 2013 |
| 120 | IPES0144 | AGATCCCATCTCCCTGTCCT | TCCTGTGATTGAACAGCAGC | 60.0 | Rajaram et al., 2013 |
| 121 | IPES0145 | TCTTGGGATCCGATGATGA | ACAAAGCCACAGCACAACAG | 59.9 | Rajaram et al., 2013 |
| 122 | IPES0146 | CATCAGAATACGGACGCCTT | CATCAGCTTTGGAGTCAGCA | 60.1 | Rajaram et al., 2013 |
| 123 | IPES0147 | GAGGAGCACAAAGAAGCACC | GACTGAAAAATTGGGAGGCA | 60.1 | Rajaram et al., 2013 |
| 124 | IPES0151 | CACCATGCATCACAAGCTG | ATATCGCCCAAATCAACCAA | 60.2 | Rajaram et al., 2013 |
| 125 | IPES0152 | GATACGAAGGGAAGCACAGC | TGTGTGGTAAGCTGCTGGAG | 60.0 | Rajaram et al., 2013 |
| 126 | IPES0153 | CTCTTTGGTCAGTGCGTCAA | CATCGAACACAGGGCATCTA | 59.7 | Rajaram et al., 2013 |
| 127 | IPES0154 | CTCGTTCTCGAAGCTCTGCT | CGCTAGGTTGGCAACTTGAT | 60.3 | Rajaram et al., 2013 |
| 128 | IPES0156 | TTAATGATGAGGATGGCGTG | CGTCAGGCAAATCTTCAGTG | 59.4 | Rajaram et al., 2013 |
| 129 | IPES0157 | GCGTGAAAGCAGCAGCAG | AGGAGGGAAATGGAGAAGGA | 60.0 | Rajaram et al., 2013 |
| 130 | IPES0160 | ACGCAATCACAGAACACCAC | TGGGAATTAATGGAAGTCGAG | 59.0 | Rajaram et al., 2013 |
| **S.No.** | **Primer name** | **Forward primer_sequence** | **Reverse primer_sequence** | **Annealing temperature (°C)** | **Source** |
| 131 | IPES0161 | GGATCCATCCATCATCACCT | TCAGGGGAACCAATTAACCA | 60.2 | Rajaram et al., 2013 |
| 132 | IPES0162 | CGCATCAGGAAAATGGAATC | CGACCTAAACTGACCTGGGA | 60.1 | Rajaram et al., 2013 |
| 133 | IPES0163 | AAGATCAAGGCCAGCAACTG | GAGAGTGCACCTGTGCAAAA | 60.0 | Rajaram et al., 2013 |
| 134 | IPES0166 | CCCGCTGATAGATGACGAAT | CAGAAAGGCCTCACTTTTCG | 60.1 | Rajaram et al., 2013 |
| 135 | IPES0167 | CATCAGGGTTGCTAGCCTTC | CTTTTCGAGAAGGCACTCGT | 59.6 | Rajaram et al., 2013 |
| 136 | IPES0174 | TCTGGGAAGGAGGAGGATTT | TGCTGCTGCTCTCTGACTGT | 60.1 | Rajaram et al., 2013 |
| 137 | IPES0175 | GGGGGTTTTTCAATCAACCT | GAAGAACTGCTGCTGTGGTG | 59.6 | Rajaram et al., 2013 |
| 138 | IPES0176 | TGGTGCAAGAATGACCATGT | CGCAGGATTACAAACATCCA | 59.5 | Rajaram et al., 2013 |
| 139 | IPES0179 | TGTACATGTCAGGATCGCGT | CTATCCTAGCCCGGTGTTCA | 60.1 | Rajaram et al., 2013 |
| 140 | IPES0180 | AACACAAAAAGGCTTCCCCT | CAGATGATAGATCCGCGATG | 59.2 | Rajaram et al., 2013 |
| 141 | IPES0181 | CTCCTCAAACACATCCAGCA | AAGGTGCTGCTCTTGTAGCC | 59.6 | Rajaram et al., 2013 |
| 142 | IPES0185 | TCTGCTTGTGTTTTACCCCC | CGTTGGTACCCATGATTTTCA | 60.6 | Rajaram et al., 2013 |
| 143 | IPES0186 | AGCATATGGCATCCTTTTCG | TTTCAGGCTTGGATTCAATGT | 59.6 | Rajaram et al., 2013 |
| 144 | IPES0189 | AGCAAGCAAGCTCTACCTCG | TTGATCAATCACCCCCAAAT | 60.0 | Rajaram et al., 2013 |
| 145 | IPES0191 | GAAGAACCTCCAGCTTTCCC | TTCTTTCCTTCAGCCTCTGC | 59.7 | Rajaram et al., 2013 |
| 146 | IPES0192 | AATTCCGTACTGTCGCCAAC | AACTTGAACGACACAATTCAAAG | 58.4 | Rajaram et al., 2013 |
| 147 | IPES0195 | GGGAATGATTGATGGGAGTG | GACTAGAGATGCCGGGCTTT | 60.7 | Rajaram et al., 2013 |
| 148 | IPES0197 | GTGTTCTTCCGAATCCGTGT | CGCTTTGCATTGAACACAGT | 59.9 | Rajaram et al., 2013 |
| 149 | IPES0198 | GGGGAGCTCTCTCTGAACTG | GAACCGCTTCTTCATCCATC | 59.6 | Rajaram et al., 2013 |
| 150 | IPES0200 | GCGCTTTCAGAGTCCTGAGT | CAAGTCGTCACGGCCTTATT | 60.1 | Rajaram et al., 2013 |
| 151 | IPES0203 | CCCTCGAAGAGATCGAAGTG | CTGAAACAACAGCCTGCAAA | 60.0 | Rajaram et al., 2013 |
| 152 | IPES0205 | CGGAACTGCTGGAGTGAAAT | ATCAACGCTCCACACACAAC | 59.6 | Rajaram et al., 2013 |
| 153 | IPES0206 | TGGGCATTGATGCTGAAATA | CAGCAATTGTTTGGCTTTGA | 59.8 | Rajaram et al., 2013 |
| 154 | IPES0207 | TTGTGGGTCCTGTGTAACCA | TACCCTTCAACGCCATTTTC | 59.9 | Rajaram et al., 2013 |
| 155 | IPES0208 | CGAAGGAGGAGTACGACGAG | TCCACAAGGTGACCTCACTG | 59.7 | Rajaram et al., 2013 |
| 156 | IPES0210 | ATTCCTGTGATGCCGAAGAC | GCACCATGACCACAAAAATG | 59.8 | Rajaram et al., 2013 |

| **S.No.** | **Primer name** | **Forward primer_sequence** | **Reverse primer_sequence** | **Annealing temperature (°C)** | **Source** |
| --- | --- | --- | --- | --- | --- |
| 157 | IPES0213 | GTCCGGTTTGTCTCTCCTTG | TGGATCTCCCATGTCGTGTA | 59.9 | Rajaram et al., 2013 |
| 158 | IPES0214 | CTGATGGTGATGCAATGGAC | CTTCTTCCCTTCAGTGGCTG | 60.0 | Rajaram et al., 2013 |
| 159 | IPES0216 | AAAGGCAGCAGATCCCTACA | TCTTTCGTTGCATCTGTTCG | 60.0 | Rajaram et al., 2013 |
| 160 | IPES0217 | TCAGTTCCGTCCACTGTGAG | CCATAATTTCGCGGCTTG | 60.2 | Rajaram et al., 2013 |
| 161 | IPES0218 | CCTGGGAACACAAAACCAGA | CCAGGTCCATGTCCTTGACT | 60.0 | Rajaram et al., 2013 |
| 162 | IPES0219 | GGGGAAGAGATAGGGTTGGT | AGCTGGGCAATAGCGAGAT | 60.0 | Rajaram et al., 2013 |
| 163 | IPES0220 | GTGGTCGATGGACTGCTTTT | CGAGAGATTCACACCAAGCA | 60.0 | Rajaram et al., 2013 |
| 164 | IPES0221 | TTTTCCCTCTTCTTGGCTCTT | CGATCTTCTGGCTCAACTCC | 60.0 | Rajaram et al., 2013 |
| 165 | IPES0223 | ATTTCCTTGGGATTGTGCAG | ATTTCCTTTCCCCAAACGAC | 60.2 | Rajaram et al., 2013 |
| 166 | IPES0224 | GCAATTAGCGGTTCTTCTCG | TGTAGGCCTCCATAACCTGG | 59.9 | Rajaram et al., 2013 |
| 167 | IPES0225 | CAAACCTCAAGCTAGGCGAC | CATGCATACACCAGTGCCAT | 60.4 | Rajaram et al., 2013 |
| 168 | IPES0226 | CACCAAACAGCATCAAGCAG | AGGAGGGTAAACACACGCAC | 60.0 | Rajaram et al., 2013 |
| 169 | IPES0227 | GCTGAGGTGGAGAAGTTTGC | GGGGGTGGTTATGAGCCTAT | 60.0 | Rajaram et al., 2013 |
| 170 | IPES0229 | ATTAGCCGACGGAGGTGAC | AGTAGATTAATTAAGAAGTGCATGTCC | 57.7 | Rajaram et al., 2013 |
| 171 | IPES0230 | GAAACTGTCTTGGGCCAGTC | GGCCGGAAGGAAAAGAATA | 59.1 | Rajaram et al., 2013 |
| 172 | IPES0233 | TCCATCATCCATGTGAGACC | TCAAAGATGAATGTATTACACAAACT | 56.3 | Rajaram et al., 2013 |
| 173 | IPES0236 | GGCCAGCTCGCCTAGAT | AGATCCACCGCCTAATGAAA | 59.5 | Rajaram et al., 2013 |
| 174 | PGIRD12 | ACTCGTTCGGATGCACTTCT | CGGGGAAGAGACAGGCTACT | 54.0 | Mariac et al., 2006 |
| 175 | PGIRD13 | CAGCAGCGAGAAGTTTAGCA | GCGTAGACGGCGTAGATGAT | 60.0 | Mariac et al., 2006 |
| 176 | PGIRD19 | TGAGGACCGAGAAGAAGCAT | CAACACCCAACAGAAACTGAA | 52.9 | Mariac et al., 2006 |
| 177 | PGIRD21 | GCTATTGCCACTGCTTCACA | CCACCATGCAACAGCAATAA | 53.8 | Mariac et al., 2006 |
| 178 | PGIRD25 | CGGAGCTCCTATCATTCCAA | GCAAGCCACAAGCCTATCTC | 58.0 | Mariac et al., 2006 |
| 179 | PGIRD43 | GTTCATGCAGCTTGGTTTCC | AGTGACCTGGGGTACAGACG | 53.9 | Mariac et al., 2006 |
| 180 | PGIRD44 | TCTCTCTCGGATCGCTGTG | GCTGGTTGGTAGAGGCTGAC | 53.4 | Mariac et al., 2006 |
| 181 | PGIRD46 | GAACAATTGCTTCTGTAATATTGCTT | GCCGACCAAGAACTTCATACA | 48.0 | Mariac et al., 2006 |
| 182 | PGIRD49 | AGCTCCTCGACGGAGAAAGT | GACGGTGTCGACGAAGATG | 52.0 | Mariac et al., 2006 |
| **S.No.** | **Primer name** | **Forward primer_sequence** | **Reverse primer_sequence** | **Annealing temperature (°C)** | **Source** |
| 183 | PGIRD5 | CAACCCAACCCATTATACTTATCTG | GCAACTCTTGCCTTTCTTGG | 54.3 | Mariac et al., 2006 |
| 184 | PGIRD50 | CTCTCGGTTTGACGGTTTGT | GGGGAAAACAAAGTTGCTCA | 50.0 | Mariac et al., 2006 |
| 185 | PGIRD54 | GCCTGGGATGTGTTTCTTCT | GCCTTTCATTTCCACCATGA | 53.6 | Mariac et al., 2006 |
| 186 | PGIRD56 | ATCACTCCTCGATCGGTCAC | ACCAGACACACGTGCCAGT | 53.2 | Mariac et al., 2006 |
| 187 | PGIRD57 | GGCCCCAAGTAACTTCCCTA | TCAAGCTAGGGCCAATGTCT | 54.0 | Mariac et al., 2006 |
| 188 | PGIRD7 | CGGAGACGCACTAGACTTGG | CCGGATGCTCACTTCCTTAT | 53.8 | Mariac et al., 2006 |
| 189 | PSMP2001 | CATGAAGCCAATTAGGTCTC | ACCATCTGACTTGTTCTTATCC | 48.7 | Qi et al., 2004 |
| 190 | PSMP2006 | GACTTATAGTCACTGGGAAAGCTC | GCTTTAATAACTTTGTGCGTATT | 51.0 | Qi et al., 2004 |
| 191 | PSMP2008 | GATCATGTTGTCATGAATCACC | ACACTACACCTACATACGCTCC | 50.6 | Qi et al., 2004 |
| 192 | PSMP2018 | CGCAAGACATTTTAGTATCACC | ACAGTCATCCTCAGTCGTCC | 50.8 | Qi et al., 2004 |
| 193 | PSMP2019 | TGTGCCACAGCTTGTTCCTC | CAAGCAGCCAGTTCCTCATC | 55.1 | Qi et al., 2004 |
| 194 | PSMP2027 | AGCAATCCGATAACAAGGAC | AGCTTTGGAAAAGGTGATCC | 51.4 | Qi et al., 2004 |
| 195 | PSMP2033 | CTATACCATTGAATTGAAAGGTC | CAATCTTTAGCTTTTTCAAGAGAC | 49.9 | Qi et al., 2004 |
| 196 | PSMP2040 | CATTACACGTTTCTTCAAACGC | TCTTCGGCCTAATAGCTCTAAC | 52.8 | Qi et al., 2004 |
| 197 | PSMP2043 | TCATATTCTCCTGTCTAAAACGTC | ACAAATCGTACAAGTTCCACTC | 50.6 | Qi et al., 2004 |
| 198 | PSMP2050 | TCAAACGGCATCAGACAACAAC | GGATCTCTTAGTGTGGTGGAGAGC | 57.1 | Qi et al., 2004 |
| 199 | PSMP2056 | ACCTGTAGCTTCAAAATTCAAAAA | AATTCAGTGTGATTTCGATGTTGC | 55.1 | Qi et al., 2004 |
| 200 | PSMP2059 | GGGGAGATGAGAAAACACAATCAC | TCGAGAGAGGAACCTGATCCTAA | 56.6 | Qi et al., 2004 |
| 201 | PSMP2063 | GAGCACATGAAATAGGAAGCAG | AAGGTAGTTATAGTTAGCTTGATC | 49.2 | Qi et al., 2004 |
| 202 | PSMP2064 | ACCGAATTAAAGTCATGGATCG | TTGATTCTTCTGACACAAATGAG | 52.8 | Qi et al., 2004 |
| 203 | PSMP2066 | ATATTAGAGCATTGCATCGC | GCATAGCAGCATACAGCAGCAA | 53.7 | Qi et al., 2004 |
| 204 | PSMP2069 | CCCATCTGAAATCTGGCTGAGAA | CCGTGTTCGTACATGGTTTTGC | 58.9 | Qi et al., 2004 |
| 205 | PSMP2070 | ACAGAAAAAGAGAGGCACAGGAGA | GCCACTCGATGGAAATGTGAAA | 54.4 | Qi et al., 2004 |
| 206 | PSMP2072 | GAAATCTACACAAGGGTCTCCA | GTACGGAGCAATGACATCTGAA | 53.0 | Qi et al., 2004 |
| 207 | PSMP2074 | AGGACTGTAGGAGTGTGGACAA | CCAGACCTACCAGTGAATGAGA | 52.7 | Qi et al., 2004 |
| 208 | PSMP2076 | GGAATAGTATATTGGCAAAATGTG | ATACTACACACTGTAAGCATTGTC | 49.5 | Qi et al., 2004 |

| **S.No.** | **Primer name** | **Forward primer_sequence** | **Reverse primer_sequence** | **Annealing temperature (°C)** | **Source** |
| --- | --- | --- | --- | --- | --- |
| 209 | PSMP2078 | CATGCCCATGACAGTATCTTAAT | ACTGTTCGGTTCCAAAATACTT | 51.8 | Qi et al., 2004 |
| 210 | PSMP2081 | CTGTGCTGTCATTGTTACCA | TCAGATCACCTATTACTTTCCCT | 49.7 | Qi et al., 2004 |
| 211 | PSMP2084 | AATCTAGTGATCTAGTGTGCTTCC | GGTTAGTTTGTTTGAGGCAAATGC | 54.1 | Qi et al., 2004 |
| 212 | PSMP2086 | CGCTTGTTTTCCGGGCTTGCTGTT | CCTTCTCAGATCCTGTGCTTTCTT | 58.3 | Qi et al., 2004 |
| 213 | PSMP2086.1 | CGCTTGTTTTCCGGGCTTGCTGTT | CGCTTGTTTTCCTTTCTTGCTGTT | 62.2 | Qi et al., 2004 |
| 214 | PSMP2088 | AAGAAGCCACCAGCACAAAA | TGCATGAAAGTAGAGGATGGTAAA | 54.5 | Qi et al*.* 2004 |
| 215 | PSMP2089 | TTCGCCGCGGCTACATACTT | TGTGCATGTTGCTGGTCATT | 51.8 | Qi et al*.* 2004 |
| 216 | PSMP2090 | AGCAGCCCAGTAATACCTCAGCTC | AGCCCTAGCGCACAACACAAACTC | 60.7 | Qi et al., 2004 |
| 217 | PSMP2201 | CCCGACGTTATGCGTTAAGTT | TCCATCCATCCATTAATCCACA | 55.8 | Qi et al*.* 2001 |
| 218 | PSMP2202 | CTGCCTGTTGAGAATAAATGAG | GTTCCGAATATAGAGCCCAAG | 51.5 | Qi et al., 2001 |
| 219 | PSMP2203 | GAACTTGATGAGTGCCACTAGC | TTGTGTAGGGAGCAACCTTGAT | 54.0 | Qi et al., 2001 |
| 220 | PSMP2205 | AGGTGCTCACGAGCTGTAAGAG | AGCAAGACACTATTTTACCATC | 51.2 | Qi et al., 2001 |
| 221 | PSMP2206 | AGAAGAAGAGGGGGTAAGAAGGAG | AGCAACATCCGTAGAGGTAGAAG | 55.0 | Qi et al., 2001 |
| 222 | PSMP2209 | TTGGACGATTTGGAAGCATAG | GAGGAAAAGAGCCATACAGAGAC | 53.6 | Qi et al., 2001 |
| 223 | PSMP2211 | CTGCATGACGTGTGACCAATACC | AACAAATCAGCACCAGCCTCC | 58.2 | Allouis et al*.* 2001 |
| 224 | PSMP2213 | CCCAAAAGAACCACACCCAC | GTTGATGCTACTGCTCGTTTG | 54.3 | Qi et al., 2001 |
| 225 | PSMP2214 | CGCACAGTACGTGTGAGTGAAG | GATTGAGCAGCAAAAACCAGC | 55.9 | Qi et al., 2001 |
| 226 | PSMP2215 | CCACGTCATTAGAGTAATCCGAG | ACTCAAATCCCAATCTTGAATC | 52.8 | Allouis et al*.* 2001 |
| 227 | PSMP2223 | CATGCTTCTTCTTCTTTTGTAACC | CAGCTCTTTGATCTCACTACAC | 50.6 | Allouis et al*.* 2001 |
| 228 | PSMP2224 | GGCGAAATTGGAATTCAGATTG | CGTAATCGTAGCGTCTCGTCTAA | 56.2 | Qi et al., 2001 |
| 229 | PSMP2225 | CCGTACTGATGATACTGATGGTT | TGGGAGGTAAGCTCAGTAGTGT | 52.1 | Allouis et al*.* 2001 |
| 230 | PSMP2227 | ACACCAAACACCAACCATAAAG | TCGTCAGCAATCACTAATGACC | 48.0 | Allouis et al*.* 2001 |
| 231 | PSMP2229 | CCACTACCATCGTCTTCCTCCATTC | GTCCGTTCCGTTAGTTGTTGCC | 60.0 | Allouis et al*.* 2001 |
| 232 | PSMP2231 | TTGCCTGAAGACGTGCAATCGTCC | CTTAATGCGTCTAGAGAGTTAAGTTG | 51.0 | Allouis et al*.* 2001 |
| 233 | PSMP2232 | TGTTGTTGGGAGAGGGTATGAG | CTCTCGCCATTCTTCAAGTTCA | 50.0 | Allouis et al*.* 2001 |
| 234 | PSMP2233 | TGTTTTCTCCTCTTAGGCTTCGTTC | ACCTTCTCCGCCACTAAACAACT | 57.6 | Allouis et al*.* 2001 |

| **S.No.** | **Primer name** | **Forward primer_sequence** | **Reverse primer_sequence** | **Annealing temperature (°C)** | **Source** |
| --- | --- | --- | --- | --- | --- |
| 235 | PSMP2235 | GCTTTTCTGCTTCTCCGTAGAC | CCCAACAATAGCCACCAATAAAGA | 56.0 | Allouis et al*.* 2001 |
| 236 | PSMP2236 | ATAAGTGGGACCCACATGCAGCAC | CGAAAGACTAGCAAAATTGCGCCTTC | 54.0 | Allouis et al*.* 2001 |
| 237 | PSMP2237 | TGGCCTTGGCCTTTCCACGCTT | CAATCAGTCCGTAGTCCACACCCCA | 56.0 | Allouis et al*.* 2001 |
| 238 | PSMP2240 | AGCCCAAAAGAAGTGGTCTAAC | CAACCACTAAAGTCTTACTGAACC | 52.0 | Allouis et al*.* 2001 |
| 239 | PSMP2248 | TCTGTTTGTTTGGGTCAGGTCCTTC | CGAATACGTATGGAGAACTGCGCATC | 58.0 | Allouis et al*.* 2001 |
| 240 | PSMP2249 | CAGTCTCTAACAAACAAACACGGC | GACAGCAACCAACTCCAAACTCCA | 58.9 | Allouis et al*.* 2001 |
| 241 | PSMP2255 | CATCTAAACACAACCAATCTTGAAC | TGGCACTCTTAAATTGACGCAT | 54.8 | Allouis et al*.* 2001 |
| 242 | PSMP2261 | AATGAAAATCCATCCCATTTCGCC | CGAGGACGAGGAGGGCGATT | 54.0 | Allouis et al*.* 2001 |
| 243 | PSMP2263 | AAAGTGAATACGATACAGGAGCTGAG | CATTTCAGCCGTTAAGTGAGACAA | 50.0 | Allouis et al*.* 2001 |
| 244 | PSMP2266 | CAAGGATGGCTGAAGGGCTATG | TTTCCAGCCCACACCAGTAATC | 58.0 | Allouis et al*.* 2001 |
| 245 | PSMP2267 | GGAAGGCGTAGGGATCAATCTCAC | ATCCACCCGACGAAGGAAACGA | 60.0 | Allouis et al*.* 2001 |
| 246 | PSMP2270 | AACCAGAGAAGTACATGGCCCG | CGACGAACAAATTAAGGCTCTC | 56.7 | Allouis et al*.* 2001 |
| 247 | PSMP2271 | CCTTATATTGGACCGACTGCTGAC | CTCCCCCATACACGAGCGAGAA | 54.0 | Allouis et al*.* 2001 |
| 248 | PSMP2273 | AACCCCACCAGTAAGTTGTGCTGC | GATGACGACAAGACCTTCTCTCC | 56.0 | Allouis et al*.* 2001 |
| 249 | PSMP2274 | CACCTAGACTCTACACAATGCAAC | AATATCAAGTGATCCACCTCCCAA | 54.8 | Allouis et al*.* 2001 |
| 250 | PSMP3017 | CACCAAACAGCATCAAGCAG | AGGTAGCCGAGGAAGGTGAG | 54.2 | Qi et al*.* 2001 |
| 251 | SiNRAMP-6 | GGTTCGGAAAATGGAGTTCA | TAGTCCCCGTGATTGACACA | 59.0 | This study |
| 252 | SiNRAMP-7 | GACAAGCAGTTGGGGTTGTT | CTTGCTCCTTCGAAACCAAG | 60.0 | This study |
| 253 | SiNAS-2 | GCAACTACCTCGACCTGAGC | GTCCACGACGGGGTACAG | 62.0 | This study |
| 254 | SiRO-1 | CGGTCTTCCTCTTCATGCTC | CCTCTGTTCTGCTCCTGGAC | 63.0 | This study |
| 255 | SiZIP-3 | CCTCGTGCTCACATCCTTCT | GAACGAGAGCATGAGCGAGT | 62.0 | This study |
| 256 | SiZIP-5 | CTCTGGTGTGGGTGTTTCCT | TTCAGTCAGTGCACAAAACAAA | 56.5 | This study |
| 257 | SiZIP-8 | TTCATGGTCACTGCCTACCA | CATGCTATGAGCCCCATTTT | 59.0 | This study |

| **S.No.** | **Primer name** | **Forward primer_sequence** | **Reverse primer_sequence** | **Annealing temperature (°C)** | **Source** |
| --- | --- | --- | --- | --- | --- |
| 258 | SiZIP-9 | TTTCATGGCTCCTGCTCTTT | CTCTCCTCGTCTCCAGGTTG | 61.0 | This study |
| 259 | SiZIP-10 | ATCATCGCCATCTTCTCCAT | GTGATGATGGCCTGGAGTCT | 60.0 | This study |
| 260 | SiYSL-8 | TTCTTCCTGGGGTCCTTCTT | CAGAGGAACTCCTCGACCTG | 62.0 | This study |
| 261 | SiYSL-18 | TCATCAACAGCTTCCACTGC | CCGACGTAAGTTCCGTTCAT | 60.0 | This study |
| 262 | SbNAC-5 | TATGGTGGGGAGAAGGAGTG | ACTCGTGCATGATCCAGTTG | 61.0 | This study |
| 263 | SbNRAMP-1 | AGGGACAGCTTTTGCTTTCA | CATGCTGCCATAACGAACAC | 59.0 | This study |
| 264 | SbNRAMP-5 | TGATCTCGATGCTGGTGTTC | AGCAGAGTGCAGGAACAGGT | 61.0 | This study |
| 265 | SbVIT-1 | AAGAGCGAGGCTGACCACTA | CCGAGCTCGAACTTCATCAT | 61.0 | This study |
| 266 | ZmZIP4 | TTCAACTCAGCCTCAGCAGGAATTTTG | ATCTTGTTTGAGAAAATGGAGAACTCTTGGAC | 59.0 | Mondal et al., 2015 |
| 267 | ZmZIP9 | TGACTACTGGATGGTCATAGCGTGCG | CTTCTGGAGCCGAGTTACAGTAAGGAGTG | 62.5 | Mondal et al., 2015 |
