# Supplementary material for: Deciphering Genomic Regions for High Grain Iron and Zinc Content Using Association Mapping in Pearl Millet
Source: Front Plant Sci. 2017 May 1;8:412. doi: 10.3389/fpls.2017.00412 (PMC5410614; doi:10.3389/fpls.2017.00412)
Supplement: Table S3 — Summary statistics of 114 SSR markers used in the study. [file Table3.docx]

**TABLE S 3│Summary statistics of 114 SSR markers used in the study**

| **S.No** | **Marker** | **MAF** | **PIC** | **Het** | **Nei**** | **na*** | **ne*** |
| --- | --- | --- | --- | --- | --- | --- | --- |
| 1 | *Xicmp* 3002 | 0.68 | 0.36 | 0.00 | 0.45 | 3.00 | 1.81 |
| 2 | *Xicmp* 3037 | 0.80 | 0.28 | 0.02 | 0.32 | 3.00 | 1.48 |
| 3 | *Xicmp* 3048 | 0.71 | 0.33 | 0.01 | 0.41 | 2.00 | 1.70 |
| 4 | *Xicmp* 3050 | 0.81 | 0.27 | 0.01 | 0.31 | 3.00 | 1.45 |
| 5 | *Xicmp* 3056 | 0.94 | 0.11 | 0.00 | 0.12 | 2.00 | 1.13 |
| 6 | *Xicmp* 3066 | 0.49 | 0.50 | 0.11 | 0.58 | 3.00 | 2.41 |
| 7 | *Xicmp* 3077 | 0.81 | 0.27 | 0.01 | 0.31 | 3.00 | 1.45 |
| 8 | *Xicmp* 3080 | 0.62 | 0.40 | 0.02 | 0.49 | 3.00 | 1.97 |
| 9 | *Xicmp* 3086 | 0.51 | 0.37 | 0.00 | 0.50 | 2.00 | 2.00 |
| 10 | *Xicmp* 3092 | 0.62 | 0.36 | 0.02 | 0.47 | 2.00 | 1.89 |
| 11 | *Xicmp* 4006 | 0.66 | 0.35 | 0.04 | 0.45 | 2.00 | 1.82 |
| 12 | *Xipes* 0007 | 0.73 | 0.32 | 0.00 | 0.39 | 2.00 | 1.65 |
| 13 | *Xipes* 0009 | 0.78 | 0.29 | 0.00 | 0.35 | 2.00 | 1.53 |
| 14 | *Xipes* 0011 | 0.65 | 0.45 | 0.02 | 0.51 | 3.00 | 2.05 |
| 15 | *Xipes* 0015 | 0.64 | 0.37 | 0.00 | 0.47 | 3.00 | 1.90 |
| 16 | *Xipes* 0019 | 0.66 | 0.38 | 0.00 | 0.47 | 3.00 | 1.88 |
| 17 | *Xipes* 0045 | 0.75 | 0.35 | 0.00 | 0.40 | 3.00 | 1.66 |
| 18 | *Xipes* 0052 | 0.84 | 0.26 | 0.00 | 0.28 | 3.00 | 1.40 |
| 19 | *Xipes* 0066 | 0.90 | 0.16 | 0.00 | 0.18 | 2.00 | 1.22 |
| 20 | *Xipes* 0071 | 0.94 | 0.11 | 0.00 | 0.12 | 3.00 | 1.13 |
| 21 | *Xipes* 0076 | 0.95 | 0.10 | 0.00 | 0.10 | 3.00 | 1.11 |
| 22 | *Xipes* 0079 | 0.98 | 0.03 | 0.00 | 0.03 | 2.00 | 1.03 |
| 23 | *Xipes* 0082 | 0.82 | 0.29 | 0.00 | 0.32 | 3.00 | 1.46 |
| 24 | *Xipes* 0089 | 0.76 | 0.34 | 0.00 | 0.38 | 3.00 | 1.62 |
| 25 | *Xipes* 0093 | 0.56 | 0.40 | 0.00 | 0.51 | 3.00 | 2.05 |
| 26 | *Xipes* 0096 | 0.81 | 0.26 | 0.01 | 0.31 | 2.00 | 1.44 |
| 27 | *Xipes* 0097 | 0.66 | 0.37 | 0.08 | 0.46 | 3.00 | 1.86 |
| 28 | *Xipes* 0101 | 0.71 | 0.33 | 0.11 | 0.41 | 2.00 | 1.71 |
| 29 | *Xipes* 0102 | 0.90 | 0.16 | 0.08 | 0.17 | 2.00 | 1.21 |
| 30 | *Xipes* 0105 | 0.53 | 0.38 | 0.10 | 0.50 | 3.00 | 2.02 |
| 31 | *Xipes* 0114 | 0.58 | 0.40 | 0.06 | 0.51 | 3.00 | 2.03 |
| 32 | *Xipes* 0117 | 0.55 | 0.37 | 0.10 | 0.50 | 2.00 | 1.98 |
| 33 | *Xipes* 0126 | 0.64 | 0.48 | 0.06 | 0.53 | 4.00 | 2.13 |
| 34 | *Xipes* 0129 | 0.61 | 0.37 | 0.06 | 0.48 | 3.00 | 1.93 |
| 35 | *Xipes* 0142 | 0.63 | 0.36 | 0.07 | 0.47 | 2.00 | 1.88 |
| 36 | *Xipes* 0144 | 0.65 | 0.37 | 0.08 | 0.46 | 3.00 | 1.86 |
| 37 | *Xipes* 0145 | 0.73 | 0.33 | 0.05 | 0.40 | 3.00 | 1.68 |
| 38 | *Xipes* 0146 | 0.52 | 0.37 | 0.01 | 0.50 | 2.00 | 2.00 |
| 39 | *Xipes* 0147 | 0.90 | 0.16 | 0.01 | 0.17 | 2.00 | 1.21 |
| 40 | *Xipes* 0152 | 0.75 | 0.32 | 0.01 | 0.38 | 3.00 | 1.62 |
| 41 | *Xipes* 0153 | 0.92 | 0.13 | 0.02 | 0.14 | 2.00 | 1.17 |
| 42 | *Xipes* 0154 | 0.75 | 0.30 | 0.00 | 0.37 | 2.00 | 1.59 |
| 43 | *Xipes* 0156 | 0.55 | 0.37 | 0.00 | 0.50 | 2.00 | 1.98 |
| 44 | *Xipes* 0157 | 0.74 | 0.31 | 0.02 | 0.39 | 2.00 | 1.63 |
| 45 | *Xipes* 0160 | 0.63 | 0.38 | 0.01 | 0.48 | 3.00 | 1.92 |
| 46 | *Xipes* 0161 | 0.94 | 0.10 | 0.01 | 0.11 | 2.00 | 1.12 |
| 47 | *Xipes* 0162 | 0.88 | 0.19 | 0.01 | 0.21 | 3.00 | 1.27 |
| 48 | *Xipes* 0163 | 0.50 | 0.56 | 0.02 | 0.63 | 3.00 | 2.68 |
| 49 | *Xipes* 0166 | 0.77 | 0.29 | 0.00 | 0.36 | 2.00 | 1.55 |
| 50 | *Xipes* 0167 | 0.92 | 0.14 | 0.01 | 0.15 | 2.00 | 1.17 |
| 51 | *Xipes* 0174 | 0.82 | 0.25 | 0.04 | 0.30 | 2.00 | 1.42 |
| 52 | *Xipes* 0175 | 0.93 | 0.12 | 0.00 | 0.13 | 2.00 | 1.15 |
| 53 | *Xipes* 0179 | 0.88 | 0.19 | 0.03 | 0.22 | 2.00 | 1.28 |
| **S.No** | **Marker** | **MAF** | **PIC** | **Het** | **Nei**** | **na*** | **ne*** |
| 54 | *Xipes* 0180 | 0.84 | 0.26 | 0.02 | 0.28 | 3.00 | 1.39 |
| 55 | *Xipes* 0181 | 0.51 | 0.39 | 0.02 | 0.51 | 3.00 | 2.03 |
| 56 | *Xipes* 0185 | 0.75 | 0.32 | 0.00 | 0.38 | 3.00 | 1.61 |
| 57 | *Xipes* 0186 | 0.89 | 0.17 | 0.00 | 0.19 | 2.00 | 1.24 |
| 58 | *Xipes* 0189 | 0.84 | 0.23 | 0.00 | 0.27 | 2.00 | 1.37 |
| 59 | *Xipes* 0191 | 0.72 | 0.32 | 0.02 | 0.40 | 2.00 | 1.68 |
| 60 | *Xipes* 0192 | 0.89 | 0.18 | 0.04 | 0.20 | 2.00 | 1.25 |
| 61 | *Xipes* 0195 | 0.96 | 0.07 | 0.00 | 0.07 | 2.00 | 1.08 |
| 62 | *Xipes* 0197 | 0.74 | 0.31 | 0.00 | 0.39 | 2.00 | 1.63 |
| 63 | *Xipes* 0198 | 0.58 | 0.39 | 0.00 | 0.50 | 3.00 | 1.99 |
| 64 | *Xipes* 0200 | 0.46 | 0.57 | 0.07 | 0.64 | 4.00 | 2.81 |
| 65 | *Xipes* 0203 | 0.80 | 0.29 | 0.01 | 0.33 | 3.00 | 1.49 |
| 66 | *Xipes* 0205 | 0.88 | 0.20 | 0.02 | 0.22 | 3.00 | 1.28 |
| 67 | *Xipes* 0206 | 0.79 | 0.28 | 0.01 | 0.33 | 2.00 | 1.50 |
| 68 | *Xipes* 0207 | 0.97 | 0.06 | 0.01 | 0.07 | 2.00 | 1.07 |
| 69 | *Xipes* 0208 | 0.62 | 0.36 | 0.09 | 0.47 | 2.00 | 1.90 |
| 70 | *Xipes* 0210 | 0.90 | 0.16 | 0.00 | 0.18 | 2.00 | 1.22 |
| 71 | *Xipes* 0213 | 0.97 | 0.06 | 0.00 | 0.06 | 2.00 | 1.06 |
| 72 | *Xipes* 0217 | 0.68 | 0.42 | 0.00 | 0.47 | 3.00 | 1.90 |
| 73 | *Xipes* 0218 | 0.80 | 0.31 | 0.00 | 0.34 | 3.00 | 1.51 |
| 74 | *Xipes* 0223 | 0.85 | 0.22 | 0.00 | 0.25 | 2.00 | 1.33 |
| 75 | *Xipes* 0224 | 0.71 | 0.34 | 0.01 | 0.42 | 3.00 | 1.72 |
| 76 | *Xipes* 0225 | 0.85 | 0.22 | 0.05 | 0.25 | 2.00 | 1.33 |
| 77 | *Xipes* 0226 | 0.86 | 0.22 | 0.00 | 0.24 | 3.00 | 1.32 |
| 78 | *Xipes* 0229 | 0.90 | 0.16 | 0.02 | 0.18 | 2.00 | 1.22 |
| 79 | *Xipes* 0236 | 0.84 | 0.23 | 0.01 | 0.27 | 2.00 | 1.36 |
| 80 | *Xpgird* 13 | 0.84 | 0.26 | 0.02 | 0.28 | 3.00 | 1.39 |
| 81 | *Xpgird* 25 | 0.88 | 0.21 | 0.00 | 0.22 | 3.00 | 1.29 |
| 82 | *Xpgird* 46 | 0.86 | 0.21 | 0.00 | 0.24 | 2.00 | 1.31 |
| 83 | *Xpgird* 49 | 0.92 | 0.14 | 0.00 | 0.15 | 2.00 | 1.18 |
| 84 | *Xpgird* 50 | 0.78 | 0.33 | 0.08 | 0.36 | 4.00 | 1.56 |
| 85 | *Xctm* 59 | 0.89 | 0.19 | 0.00 | 0.20 | 3.00 | 1.25 |
| 86 | *Xpsmp* 2018 | 0.82 | 0.25 | 0.02 | 0.29 | 2.00 | 1.41 |
| 87 | *Xpsmp* 2027 | 0.89 | 0.18 | 0.02 | 0.20 | 2.00 | 1.25 |
| 88 | *Xpsmp* 2033 | 0.93 | 0.12 | 0.00 | 0.13 | 2.00 | 1.15 |
| 89 | *Xpsmp* 2040 | 0.85 | 0.23 | 0.00 | 0.26 | 2.00 | 1.35 |
| 90 | *Xpsmp* 2043 | 0.82 | 0.26 | 0.01 | 0.30 | 3.00 | 1.43 |
| 91 | *Xpsmp* 2056 | 0.63 | 0.47 | 0.02 | 0.53 | 3.00 | 2.14 |
| 92 | *Xpsmp* 2066 | 0.32 | 0.68 | 0.01 | 0.73 | 4.00 | 3.75 |
| 93 | *Xpsmp* 2074 | 0.64 | 0.38 | 0.00 | 0.48 | 3.00 | 1.91 |
| 94 | *Xpsmp* 2076 | 0.82 | 0.26 | 0.00 | 0.30 | 3.00 | 1.44 |
| 95 | *Xpsmp* 2081 | 0.42 | 0.60 | 0.02 | 0.67 | 5.00 | 2.99 |
| 96 | *Xpsmp* 2086 | 0.47 | 0.57 | 0.12 | 0.26 | 3.00 | 1.36 |
| 97 | *Xpsmp* 2086.1 | 0.51 | 0.23 | 0.24 | 0.23 | 2.00 | 1.3 |
| 98 | *Xpsmp* 2090 | 0.84 | 0.24 | 0.00 | 0.27 | 3.00 | 1.38 |
| 99 | *Xpsmp* 2202 | 0.84 | 0.23 | 0.01 | 0.27 | 2.00 | 1.36 |
| 100 | *Xpsmp* 2205 | 0.98 | 0.04 | 0.00 | 0.05 | 3.00 | 1.05 |
| 101 | *Xpsmp* 2206 | 0.73 | 0.32 | 0.00 | 0.39 | 2.00 | 1.65 |
| 102 | *Xpsmp* 2209 | 0.59 | 0.39 | 0.00 | 0.50 | 3.00 | 1.98 |
| 103 | *Xpsmp* 2224 | 0.85 | 0.22 | 0.01 | 0.15 | 3.00 | 1.18 |
| 104 | *Xpsmp* 2227 | 0.94 | 0.11 | 0.02 | 0.12 | 2.00 | 1.13 |
| 105 | *Xpsmp* 2231 | 0.62 | 0.36 | 0.00 | 0.47 | 2.00 | 1.90 |
| 106 | *Xpsmp* 2232 | 0.75 | 0.31 | 0.00 | 0.38 | 2.00 | 1.61 |
| 107 | *Xpsmp* 2236 | 0.67 | 0.35 | 0.02 | 0.45 | 3.00 | 1.80 |
| **S.No** | **Marker** | **MAF** | **PIC** | **Het** | **Nei**** | **na*** | **ne*** |
| 108 | *Xpsmp* 2237 | 0.56 | 0.38 | 0.00 | 0.50 | 3.00 | 2.00 |
| 109 | *Xpsmp* 2261 | 0.92 | 0.14 | 0.02 | 0.25 | 2.00 | 1.33 |
| 110 | *Xpsmp* 2263 | 0.35 | 0.59 | 0.02 | 0.67 | 3.00 | 2.99 |
| 111 | *Xpsmp* 2266 | 0.72 | 0.35 | 0.01 | 0.42 | 3.00 | 1.71 |
| 112 | *Xpsmp* 2267 | 0.80 | 0.28 | 0.07 | 0.32 | 3.00 | 1.47 |
| 113 | *Xpsmp* 2271 | 0.77 | 0.30 | 0.08 | 0.36 | 3.00 | 1.56 |
| 114 | *Xpsmp* 2273 | 0.70 | 0.42 | 0.07 | 0.46 | 3.00 | 1.85 |
|  | **Min** | **0.32** | **0.03** | **0.00** | **0.03** | **2.00** | **1.03** |
|  | **Max** | **0.98** | **0.68** | **0.12** | **0.73** | **5.00** | **3.75** |
|  | **Mean** | **0.75** | **0.3** | **0.02** | **0.34** | **2.6** | **1.63** |

MAF = Major allele frequency

PIC = Polymorphism Information Content

Het = Observed heterozygosity

* na = Observed number of alleles

* ne = Effective number of alleles

** Nei's (1973) expected heterozygosity
