# Supplementary material for: Deciphering Genomic Regions for High Grain Iron and Zinc Content Using Association Mapping in Pearl Millet
Source: Front Plant Sci. 2017 May 1;8:412. doi: 10.3389/fpls.2017.00412 (PMC5410614; doi:10.3389/fpls.2017.00412)
Supplement: Table S4A — One way ANOVA for grain iron content (ppm) for six environments. [file Table4.docx]

**TABLE S 4A │One way ANOVA for grain iron content (ppm) for six environments**

| **Source of variation** | **df** | **Del_14** | **Del_15** | **Jod_14** | **Jod_15** | **DW_14** | **DW_15** |
| --- | --- | --- | --- | --- | --- | --- | --- |
| Replication | 2 | 47.99 | 10.52 | 67.16 | 26.47 | 24.03 | 20.59 |
| Block within Rep. | 36 | 36.25 | 6.14 | 28.05 | 1.55 | 30.75 | 2.55 |
| Genotype | 129 | 892.78** | 769.71** | 1174.11** | 749.48** | 1395.88** | 839.87** |
| Error |  | 48.74 | 35.44 | 32.67 | 22.51 | 31.01 | 37.50 |
| CV |  | 12.12 | 9.86 | 10.25 | 8.20 | 9.13 | 9.96 |

*** Significant at p<.05 **Significant at p<0.01**

Where, Del_14_Fe, Del_15_Fe, Jod_14_Fe, Jod_15_Fe, DW_14_Fe and DW_15_Fe are iron content at Delhi during 2014, Delhi during 2015, Jodhpur during 2014, Jodhpur during 2015, Dharwad during 2014 and Dharwad during 2015 respectively.

**TABLE S 4B │One way ANOVA for grain zinc content (ppm) for six environments**

| **Source of variation** | **df** | **Del_14** | **Del_15** | **Jod_14** | **Jod_15** | **DW_14** | **DW_15** |
| --- | --- | --- | --- | --- | --- | --- | --- |
| Replication | 2 | 40.99 | 8.06 | 24.49 | 13.61 | 21.49 | 9.19 |
| Block within Rep. | 36 | 23.14 | 17.08 | 17.39 | 4.55 | 10.58 | 3.24 |
| Genotype | 129 | 389.94** | 478.76** | 453.46** | 410.82** | 330.57** | 493.82** |
| Error |  | 22.11 | 23.96 | 20.75 | 25.23 | 15.82 | 19.16 |
| CV |  | 10.08 | 9.89 | 10.65 | 11.07 | 10.90 | 12.24 |

| *** Significant at p<.05 **Significant at p<0.01** |
| --- |

Where, Del_14_Zn, Del_15_Zn, Jod_14_Zn, Jod_15_Zn, DW_14_Zn and DW_15_Zn are zinc content at Delhi during 2014, Delhi during 2015, Jodhpur during 2014, Jodhpur during 2015, Dharwad during 2014 and Dharwad during 2015respectively.

**TABLE S4C │Pooled ANOVA of years 2014 and 2015 for grain iron and zinc content for different locations**

| **Source of variation** | **df** | **Mean sum of squares** | | | | | |
| --- | --- | --- | --- | --- | --- | --- | --- |
|  |  | **Del-mean** | | **DW-mean** | | **Jod-mean** | |
|  |  | **Fe** | **Zn** | **Fe** | **Zn** | **Fe** | **Zn** |
| Replication within Year | 4 | 11.63 | 39.66 | 13.31 | 12.34 | 34.4 | 39.5 |
| Block within Rep. within Year | 72 | 30.09 | 19.87 | 25.65 | 9.91 | 27.3 | 15.5 |
| Year | 1 | 1516.91** | 1596.77** | 51.81 | 102.03 | 850.9** | 1336.0** |
| Genotype | 129 | 1363.02** | 743.36** | 1657.22 | 722.78 | 1503.3** | 718.0** |
| Genotype x Year | 129 | 278.05** | 129.29** | 578.52 | 101.60 | 420.3** | 146.3** |
| Error | 444 | 37.23 | 22.56 | 34.25 | 17.49 | 27.6 | 23.0 |
| CV |  | 10.33 | 9.89 | 9.56 | 11.58 | 9.3 | 10.9 |

| *** Significant at p<.05 **Significant at p<0.01** |
| --- |

Where, Del-mean, DW-mean and Jod-mean are Delhi mean, Dharwad mean and Jodhpur mean respectively.

**TABLE S4D │Pooled ANOVA across three locations for grain iron and zinccontent for the years 2014 and 2015.**

| Source of variation | df | Mean sum of squares | | | |
| --- | --- | --- | --- | --- | --- |
|  |  | **Y2014-mean** | | **Y2015-mean** | |
|  |  | Fe | Zn | Fe | Zn |
| Replication within Location | 6 | 34.64 | 69.06** | 4.89 | 5.29 |
| Block within Rep. within Loc. | 108 | 35.41** | 16.93 | 19.92 | 13.26 |
| Location | 2 | 2716.21 | 10219.71** | 1379.86** | 19362.63** |
| Genotype | 129 | 2075.64** | 692.85** | 1427.96** | 771.6526** |
| Genotype x Location | 258 | 688.38** | 222.25** | 460.02** | 306.1473** |
| Error | 666 | 35.89 | 19.33 | 30.16 | 22.70 |
| CV |  | 10.31 | 10.48 | 9.17 | 10.95 |

| *** Significant at p<.05 **Significant at p<0.01** |
| --- |

**TABLE S4E │Pooled ANOVA across 6 environments (two years and three locations) for grain iron and zinc content**

| Source of variation | df | Mean sum of squares | |
| --- | --- | --- | --- |
|  |  | Fe | Zn |
| Rep. within Env. | 12 | 19.76 | 37.17* |
| Block within Rep. within Env. | 216 | 27.67 | 15.09 |
| Environment | 5 | 2016.61** | 12126.95** |
| Genotype | 129 | 2860.07** | 1261.94** |
| Genotype x Environment | 645 | 588.07** | 259.87** |
| Error | 1332 | 33.02 | 21.01 |
| CV |  | 9.74 | 10.73 |

| *** Significant at p<.05 **Significant at p<0.01** |
| --- |
