# Supplementary material for: Deciphering Genomic Regions for High Grain Iron and Zinc Content Using Association Mapping in Pearl Millet
Source: Front Plant Sci. 2017 May 1;8:412. doi: 10.3389/fpls.2017.00412 (PMC5410614; doi:10.3389/fpls.2017.00412)
Supplement: Table S5A — Mean values of grain iron content across six environments and their six pooled environments (ppm). [file Table5.docx]

**TABLE S 5A │ Mean values of grain iron content across six environments and their six pooled environments (ppm)**

| **S.No.** | **Genotype** | **Del-14** | **Del-15** | **Jod-14** | **Jod-15** | **DW-14** | **DW-15** | **Y14-M** | **Y15-M** | **Del-M** | **Jod-M** | **DW-M** | **GM** |
| --- | --- | --- | --- | --- | --- | --- | --- | --- | --- | --- | --- | --- | --- |
| 1 | PPMI 1102 | 115.53 | 117.27 | 105.30 | 95.24 | 114.10 | 123.78 | 111.60 | 112.10 | 116.40 | 100.27 | 118.94 | 111.90 |
| 2 | 5540B | 27.29 | 31.25 | 26.12 | 33.17 | 33.47 | 42.36 | 29.00 | 35.59 | 29.27 | 29.65 | 37.92 | 32.30 |
| 3 | PPMI 214 | 77.59 | 77.31 | 74.75 | 71.12 | 78.50 | 52.76 | 76.90 | 67.06 | 77.45 | 72.94 | 65.63 | 72.00 |
| 4 | IPC 1657 | 34.27 | 51.64 | 42.32 | 40.73 | 51.59 | 40.93 | 42.70 | 44.43 | 42.96 | 41.53 | 46.26 | 43.60 |
| 5 | PPMI 708 | 113.70 | 109.21 | 121.63 | 75.38 | 108.37 | 88.88 | 114.60 | 91.16 | 111.46 | 98.51 | 98.62 | 102.90 |
| 6 | PPMI 1087 | 34.75 | 57.46 | 38.76 | 39.64 | 44.14 | 45.40 | 39.20 | 47.50 | 46.11 | 39.20 | 44.77 | 43.40 |
| 7 | PPMWGI 99 | 87.64 | 87.66 | 82.35 | 72.34 | 49.53 | 47.78 | 73.20 | 69.26 | 87.65 | 77.35 | 48.65 | 71.20 |
| 8 | J2467 | 38.24 | 52.36 | 34.82 | 40.23 | 45.07 | 55.81 | 39.40 | 49.47 | 45.30 | 37.52 | 50.44 | 44.40 |
| 9 | PPMI 1225 | 102.70 | 87.26 | 76.23 | 81.34 | 76.17 | 41.36 | 85.00 | 69.99 | 94.98 | 78.79 | 58.76 | 77.50 |
| 10 | J2405 | 39.95 | 60.75 | 32.95 | 38.61 | 31.22 | 50.85 | 34.70 | 50.07 | 50.35 | 35.78 | 41.04 | 42.40 |
| 11 | PPMFeZMP 199 | 125.01 | 99.28 | 111.95 | 107.29 | 93.49 | 98.60 | 110.10 | 101.72 | 112.14 | 109.62 | 96.05 | 105.90 |
| 12 | PPMFeZMP 65 | 54.53 | 57.41 | 33.26 | 48.16 | 41.80 | 38.91 | 43.20 | 48.16 | 55.97 | 40.71 | 40.36 | 45.70 |
| 13 | ICTP 8203 Fe | 71.07 | 69.32 | 75.54 | 70.70 | 87.40 | 85.84 | 78.00 | 75.29 | 70.19 | 73.12 | 86.62 | 76.60 |
| 14 | PPMI 1222 | 38.78 | 55.65 | 60.83 | 57.25 | 55.81 | 64.72 | 51.80 | 59.21 | 47.22 | 59.04 | 60.26 | 55.50 |
| 15 | PPMI 683 | 106.59 | 103.67 | 84.84 | 81.56 | 85.89 | 82.19 | 92.40 | 89.14 | 105.13 | 83.20 | 84.04 | 90.80 |
| 16 | PPMI 627 | 36.28 | 50.14 | 31.72 | 42.39 | 57.57 | 52.84 | 41.90 | 48.46 | 43.21 | 37.06 | 55.21 | 45.20 |
| 17 | ICMR06222 | 59.30 | 68.28 | 48.98 | 61.75 | 59.61 | 48.14 | 56.00 | 59.39 | 63.79 | 55.36 | 53.87 | 57.70 |
| 18 | PPMI 660 | 52.43 | 57.76 | 50.21 | 52.37 | 56.56 | 60.14 | 53.10 | 56.76 | 55.09 | 51.29 | 58.35 | 54.90 |
| 19 | PIB 228 | 45.47 | 60.39 | 107.13 | 72.34 | 88.45 | 78.89 | 80.40 | 70.54 | 52.93 | 89.74 | 83.67 | 75.40 |
| 20 | PPMWGI 146 | 43.37 | 49.67 | 44.06 | 52.39 | 89.19 | 58.63 | 58.90 | 53.56 | 46.52 | 48.23 | 73.91 | 56.20 |
| 21 | PPMI 295 | 53.39 | 50.39 | 85.02 | 69.87 | 66.42 | 45.78 | 68.30 | 55.35 | 51.89 | 77.45 | 56.10 | 61.80 |
| 22 | PPMI 1220 | 58.74 | 60.75 | 103.09 | 61.19 | 66.15 | 38.95 | 76.00 | 53.63 | 59.75 | 82.14 | 52.55 | 64.80 |
| 23 | PPMFeZMP 37 | 64.02 | 58.94 | 69.65 | 64.85 | 97.20 | 76.15 | 77.00 | 66.65 | 61.48 | 67.25 | 86.68 | 71.80 |
| 24 | J108 | 51.46 | 43.41 | 38.44 | 43.12 | 37.52 | 44.66 | 42.50 | 43.73 | 47.44 | 40.78 | 41.09 | 43.10 |
| 25 | PPMI 85 | 40.89 | 44.89 | 36.88 | 37.61 | 45.76 | 46.28 | 41.20 | 42.93 | 42.89 | 37.25 | 46.02 | 42.10 |
| 26 | PPMI 301 | 61.23 | 67.83 | 69.78 | 63.92 | 62.53 | 65.86 | 64.50 | 65.87 | 64.53 | 66.85 | 64.20 | 65.20 |
| 27 | ICMB 98222 | 90.23 | 76.21 | 57.81 | 70.70 | 88.48 | 90.81 | 78.80 | 79.24 | 83.22 | 64.25 | 89.65 | 79.00 |
| 28 | D 23 | 43.42 | 51.21 | 49.27 | 50.23 | 51.83 | 56.15 | 48.20 | 52.53 | 47.32 | 49.75 | 53.99 | 50.40 |
| **S.No.** | **Genotype** | **Del-14** | **Del-15** | **Jod-14** | **Jod-15** | **DW-14** | **DW-15** | **Y14-M** | **Y15-M** | **Del-M** | **Jod-M** | **DW-M** | **GM** |
| 29 | PPMI 1103 | 62.27 | 58.22 | 47.88 | 52.31 | 56.34 | 78.63 | 55.50 | 63.05 | 60.25 | 50.10 | 67.49 | 59.30 |
| 30 | PPMI 1104 | 82.86 | 66.56 | 54.03 | 59.86 | 125.03 | 90.76 | 87.30 | 72.39 | 74.71 | 56.94 | 107.90 | 79.80 |
| 31 | PPMI 1105 | 57.59 | 60.74 | 32.06 | 61.34 | 41.50 | 87.87 | 43.70 | 69.98 | 59.16 | 46.70 | 64.69 | 56.80 |
| 32 | PPMI 1107 | 74.75 | 81.97 | 45.67 | 77.26 | 49.21 | 96.78 | 56.50 | 85.34 | 78.36 | 61.47 | 73.00 | 70.90 |
| 33 | PPMI 1108 | 91.21 | 70.83 | 71.43 | 84.11 | 124.94 | 119.73 | 95.90 | 91.56 | 81.02 | 77.77 | 122.34 | 93.70 |
| 34 | PPMI 1112 | 44.90 | 70.81 | 68.36 | 69.37 | 42.89 | 78.89 | 52.10 | 73.02 | 57.85 | 68.87 | 60.89 | 62.50 |
| 35 | 841B | 49.30 | 65.30 | 35.85 | 78.21 | 40.19 | 70.74 | 41.80 | 71.42 | 57.30 | 57.03 | 55.47 | 56.60 |
| 36 | 5141B | 49.85 | 68.80 | 38.11 | 67.39 | 41.18 | 66.36 | 43.00 | 67.52 | 59.33 | 52.75 | 53.77 | 55.30 |
| 37 | 5054B | 53.93 | 74.10 | 63.37 | 71.35 | 41.25 | 68.85 | 52.80 | 71.43 | 64.02 | 67.36 | 55.05 | 62.10 |
| 38 | 6030B | 60.39 | 84.80 | 44.87 | 45.28 | 46.39 | 55.13 | 50.60 | 61.74 | 72.59 | 45.08 | 50.76 | 56.10 |
| 39 | 351B | 67.04 | 77.56 | 62.41 | 111.40 | 39.09 | 66.12 | 56.20 | 85.03 | 72.30 | 86.91 | 52.61 | 70.60 |
| 40 | 379B | 62.60 | 52.23 | 85.27 | 51.34 | 48.89 | 68.87 | 65.60 | 57.48 | 57.42 | 68.31 | 58.88 | 61.50 |
| 41 | 411B | 51.02 | 62.35 | 79.48 | 48.63 | 32.37 | 48.84 | 54.30 | 53.27 | 56.69 | 64.06 | 40.61 | 53.80 |
| 42 | 576B | 48.60 | 56.46 | 46.24 | 55.21 | 39.78 | 50.89 | 44.90 | 54.19 | 52.53 | 50.72 | 45.34 | 49.50 |
| 43 | PPMI 59 | 50.63 | 42.20 | 29.23 | 53.85 | 76.95 | 42.74 | 52.30 | 46.26 | 46.42 | 41.54 | 59.85 | 49.30 |
| 44 | PPMI 265 | 76.87 | 68.24 | 73.27 | 45.28 | 56.41 | 56.35 | 68.80 | 56.62 | 72.55 | 59.27 | 56.38 | 62.70 |
| 45 | PPMI 269 | 44.41 | 57.67 | 35.43 | 52.17 | 59.81 | 63.88 | 46.60 | 57.91 | 51.04 | 43.80 | 61.85 | 52.20 |
| 46 | PPMI 275 | 55.92 | 80.15 | 59.73 | 53.39 | 38.05 | 48.89 | 51.20 | 60.81 | 68.04 | 56.56 | 43.47 | 56.00 |
| 47 | PPMI 496 | 57.49 | 52.61 | 59.23 | 47.73 | 97.20 | 78.85 | 71.30 | 59.73 | 55.05 | 53.48 | 88.02 | 65.50 |
| 48 | PPMI 694 | 60.37 | 70.34 | 46.06 | 70.23 | 38.93 | 58.18 | 48.50 | 66.25 | 65.36 | 58.15 | 48.56 | 57.40 |
| 49 | PPMI 719 | 41.24 | 79.75 | 39.92 | 57.74 | 38.78 | 47.48 | 40.00 | 61.66 | 60.49 | 48.83 | 43.13 | 50.80 |
| 50 | PPMI 744 | 46.46 | 83.92 | 33.44 | 40.23 | 75.46 | 38.96 | 51.80 | 54.37 | 65.19 | 36.83 | 57.21 | 53.10 |
| 51 | PPMI 759 | 38.27 | 65.25 | 68.54 | 41.41 | 64.01 | 45.90 | 56.90 | 50.85 | 51.76 | 54.97 | 54.95 | 53.90 |
| 52 | EGPN 423 | 51.87 | 67.45 | 70.26 | 37.62 | 57.66 | 48.80 | 59.90 | 51.29 | 59.66 | 53.94 | 53.23 | 55.60 |
| 53 | PPMWGI 152 | 44.37 | 70.13 | 78.51 | 73.62 | 54.48 | 42.67 | 59.10 | 62.14 | 57.25 | 76.06 | 48.57 | 60.60 |
| 54 | PPMWGI 100 | 51.73 | 55.63 | 73.98 | 47.39 | 53.76 | 70.87 | 59.80 | 57.96 | 53.68 | 60.69 | 62.31 | 58.90 |
| 55 | PPMWGI 108 | 57.85 | 50.14 | 61.27 | 78.34 | 62.65 | 66.77 | 60.60 | 65.08 | 53.99 | 69.80 | 64.71 | 62.80 |
| 56 | KSMWGI 14 | 51.66 | 50.48 | 67.61 | 87.56 | 120.50 | 70.76 | 79.90 | 69.60 | 51.07 | 77.59 | 95.63 | 74.80 |
| 57 | PPMI 1224 | 67.17 | 47.56 | 85.08 | 78.46 | 41.94 | 41.14 | 64.70 | 55.72 | 57.37 | 81.77 | 41.54 | 60.20 |
| **S.No.** | **Genotype** | **Del-14** | **Del-15** | **Jod-14** | **Jod-15** | **DW-14** | **DW-15** | **Y14-M** | **Y15-M** | **Del-M** | **Jod-M** | **DW-M** | **GM** |
| 58 | PPMI 1231 | 55.79 | 78.62 | 35.07 | 66.12 | 120.95 | 65.24 | 70.60 | 69.99 | 67.21 | 50.59 | 93.09 | 70.30 |
| 59 | PPMI 1233 | 63.98 | 74.45 | 70.20 | 91.13 | 31.95 | 48.87 | 55.40 | 71.48 | 69.22 | 80.67 | 40.41 | 63.40 |
| 60 | PPMI 1263 | 48.37 | 70.31 | 30.55 | 57.34 | 75.64 | 62.86 | 51.50 | 63.50 | 59.34 | 43.95 | 69.25 | 57.50 |
| 61 | ICMR07111 | 42.49 | 44.59 | 63.52 | 48.37 | 76.62 | 59.24 | 60.90 | 50.73 | 43.54 | 55.95 | 67.93 | 55.80 |
| 62 | ICMR07999 | 54.99 | 40.31 | 100.12 | 51.91 | 51.98 | 57.86 | 69.00 | 50.03 | 47.65 | 76.02 | 54.92 | 59.50 |
| 63 | PPMI 1084 | 84.60 | 90.10 | 65.42 | 75.28 | 97.96 | 47.86 | 82.70 | 71.08 | 87.35 | 70.35 | 72.91 | 76.90 |
| 64 | PPMI 1086 | 64.19 | 77.85 | 69.81 | 49.19 | 60.90 | 41.80 | 65.00 | 56.28 | 71.02 | 59.50 | 51.35 | 60.60 |
| 65 | PPMI 1089 | 71.13 | 68.26 | 35.80 | 41.71 | 64.76 | 58.91 | 57.20 | 56.29 | 69.70 | 38.76 | 61.84 | 56.80 |
| 66 | PPMI 1090 | 40.51 | 32.69 | 58.73 | 24.64 | 124.13 | 48.89 | 74.50 | 35.41 | 36.60 | 41.68 | 86.51 | 54.90 |
| 67 | PPMI 1092 | 61.09 | 70.39 | 52.53 | 38.74 | 34.75 | 55.14 | 49.50 | 54.76 | 65.74 | 45.63 | 44.95 | 52.10 |
| 68 | PPMI 1155 | 46.00 | 37.90 | 32.24 | 30.61 | 66.62 | 39.39 | 48.30 | 35.97 | 41.95 | 31.42 | 53.01 | 42.10 |
| 69 | PPMI 1161 | 39.40 | 60.75 | 42.68 | 49.16 | 53.29 | 52.78 | 45.10 | 54.23 | 50.08 | 45.92 | 53.04 | 49.70 |
| 70 | PPMI 1165 | 45.57 | 47.21 | 35.02 | 48.72 | 57.61 | 82.88 | 46.10 | 59.60 | 46.39 | 41.87 | 70.25 | 52.80 |
| 71 | PIB 686 | 50.39 | 56.63 | 32.99 | 55.14 | 43.24 | 56.23 | 42.20 | 56.00 | 53.51 | 44.06 | 49.74 | 49.10 |
| 72 | HBL 11 | 30.92 | 50.17 | 28.20 | 40.35 | 42.97 | 46.76 | 34.00 | 45.76 | 40.55 | 34.27 | 44.87 | 39.90 |
| 73 | H77/833-2-202 | 58.24 | 67.58 | 30.31 | 61.43 | 51.87 | 40.66 | 46.80 | 56.56 | 62.91 | 45.87 | 46.27 | 51.70 |
| 74 | H77/833-2 | 40.00 | 47.64 | 68.99 | 58.46 | 52.95 | 55.88 | 54.00 | 53.99 | 43.82 | 63.73 | 54.42 | 54.00 |
| 75 | J2454 | 48.23 | 75.32 | 39.94 | 53.62 | 73.27 | 63.24 | 53.80 | 64.06 | 61.77 | 46.78 | 68.26 | 58.90 |
| 76 | J2496 | 53.51 | 57.62 | 31.78 | 48.72 | 59.99 | 45.95 | 48.40 | 50.76 | 55.57 | 40.25 | 52.97 | 49.60 |
| 77 | IPC 1480 | 58.94 | 56.54 | 23.27 | 41.87 | 45.48 | 42.91 | 42.60 | 47.11 | 57.74 | 32.57 | 44.20 | 44.80 |
| 78 | PPMFeZMP 72 | 53.70 | 40.34 | 38.59 | 51.16 | 66.42 | 56.36 | 52.90 | 49.29 | 47.02 | 44.87 | 61.39 | 51.10 |
| 79 | PPMI 1218 | 54.61 | 47.36 | 51.46 | 41.16 | 50.86 | 48.86 | 52.30 | 45.79 | 50.98 | 46.31 | 49.86 | 49.10 |
| 80 | PPMI 1267 | 81.59 | 71.42 | 70.09 | 67.19 | 63.91 | 85.10 | 71.90 | 74.57 | 76.51 | 68.64 | 74.51 | 73.20 |
| 81 | PPMI 1268 | 36.99 | 57.61 | 31.77 | 56.28 | 40.16 | 47.86 | 36.30 | 53.92 | 47.30 | 44.03 | 44.01 | 45.10 |
| 82 | PPMI 1269 | 47.35 | 61.70 | 28.32 | 71.36 | 66.71 | 68.89 | 47.50 | 67.32 | 54.52 | 49.84 | 67.80 | 57.40 |
| 83 | PPMFeZMP 34 | 37.33 | 55.48 | 64.93 | 70.85 | 43.64 | 55.78 | 48.60 | 60.70 | 46.41 | 67.89 | 49.71 | 54.70 |
| 84 | PPMFeZMP 125 | 43.92 | 60.31 | 55.51 | 66.25 | 101.25 | 73.73 | 66.90 | 66.76 | 52.12 | 60.88 | 87.49 | 66.80 |
| 85 | PPMFeZMP 126 | 75.00 | 95.24 | 82.92 | 50.42 | 48.72 | 41.11 | 68.90 | 62.26 | 85.12 | 66.67 | 44.92 | 65.60 |
| 86 | PPMFeZMP 153 | 51.16 | 55.88 | 90.98 | 58.17 | 49.59 | 54.19 | 63.90 | 56.08 | 53.52 | 74.57 | 51.89 | 60.00 |
| **S.No.** | **Genotype** | **Del-14** | **Del-15** | **Jod-14** | **Jod-15** | **DW-14** | **DW-15** | **Y14-M** | **Y15-M** | **Del-M** | **Jod-M** | **DW-M** | **GM** |
| 87 | HTP 94/54 | 43.18 | 38.70 | 41.94 | 41.37 | 42.76 | 49.76 | 42.60 | 43.28 | 40.94 | 41.66 | 46.26 | 43.00 |
| 88 | PPMI 1270 | 48.51 | 56.69 | 64.15 | 36.82 | 59.74 | 50.86 | 57.50 | 48.12 | 52.60 | 50.49 | 55.30 | 52.80 |
| 89 | PPMI 1271 | 41.47 | 37.87 | 64.04 | 87.23 | 41.42 | 34.36 | 49.00 | 53.15 | 39.67 | 75.64 | 37.89 | 51.10 |
| 90 | PPMI 1272 | 48.30 | 36.90 | 64.43 | 59.19 | 44.85 | 35.30 | 52.50 | 43.80 | 42.60 | 61.81 | 40.08 | 48.20 |
| 91 | PPMFeZMP 30 | 36.13 | 29.99 | 45.89 | 36.24 | 34.09 | 37.95 | 38.70 | 34.73 | 33.06 | 41.07 | 36.02 | 36.70 |
| 92 | PPMFeZMP 35 | 51.53 | 52.90 | 40.33 | 70.43 | 55.67 | 59.97 | 49.20 | 61.10 | 52.22 | 55.38 | 57.82 | 55.10 |
| 93 | PPMFeZMP 47 | 56.41 | 55.37 | 37.92 | 48.76 | 89.28 | 69.99 | 61.20 | 58.04 | 55.89 | 43.34 | 79.64 | 59.60 |
| 94 | PPMFeZMP 87 | 54.24 | 46.23 | 39.13 | 38.32 | 90.41 | 75.98 | 61.30 | 53.51 | 50.24 | 38.73 | 83.20 | 57.40 |
| 95 | PPMI 1273 | 52.75 | 40.31 | 30.92 | 66.24 | 42.90 | 57.88 | 42.20 | 54.81 | 46.53 | 48.58 | 50.39 | 48.50 |
| 96 | PPMI 1274 | 55.32 | 54.48 | 64.68 | 77.39 | 59.48 | 84.82 | 59.80 | 72.23 | 54.90 | 71.04 | 72.15 | 66.00 |
| 97 | PPMFeZMP 143 | 63.29 | 64.29 | 45.14 | 63.13 | 42.38 | 68.84 | 50.30 | 65.42 | 63.79 | 54.14 | 55.61 | 57.80 |
| 98 | G73-107 | 49.76 | 38.29 | 36.86 | 67.23 | 41.95 | 55.11 | 42.90 | 53.54 | 44.03 | 52.05 | 48.53 | 48.20 |
| 99 | PPMI 1067 | 78.65 | 60.14 | 67.16 | 69.39 | 63.13 | 70.75 | 69.60 | 66.76 | 69.40 | 68.28 | 66.94 | 68.20 |
| 100 | PPMI 1276 | 65.29 | 70.34 | 68.44 | 84.19 | 63.87 | 59.69 | 65.90 | 71.41 | 67.81 | 76.31 | 61.78 | 68.60 |
| 101 | PPMDMGMP 27 | 76.54 | 72.56 | 75.13 | 80.46 | 45.41 | 90.11 | 65.70 | 81.04 | 74.55 | 77.79 | 67.76 | 73.40 |
| 102 | PPMDMGMP 60 | 52.98 | 59.90 | 52.52 | 77.14 | 46.89 | 68.87 | 50.80 | 68.64 | 56.44 | 64.83 | 57.88 | 59.70 |
| 103 | PPMDMGMP 87 | 55.71 | 38.70 | 40.84 | 37.46 | 51.64 | 65.64 | 49.40 | 47.27 | 47.21 | 39.15 | 58.64 | 48.30 |
| 104 | PPMDMGMP 99 | 82.19 | 56.40 | 81.76 | 58.13 | 139.12 | 59.71 | 101.00 | 58.08 | 69.30 | 69.95 | 99.42 | 79.60 |
| 105 | PPMI 1277 | 48.56 | 50.37 | 35.20 | 48.71 | 38.15 | 43.71 | 40.60 | 47.60 | 49.47 | 41.95 | 40.93 | 44.10 |
| 106 | PPMI 1278 | 50.11 | 65.30 | 71.16 | 41.64 | 40.64 | 54.82 | 54.00 | 53.92 | 57.70 | 56.40 | 47.73 | 53.90 |
| 107 | PPMI 1011 | 61.70 | 68.80 | 43.15 | 58.37 | 59.40 | 62.92 | 54.80 | 63.36 | 65.25 | 50.76 | 61.16 | 59.10 |
| 108 | ICMB 04222 | 64.23 | 74.10 | 50.52 | 47.80 | 70.98 | 52.76 | 61.90 | 58.22 | 69.17 | 49.16 | 61.87 | 60.10 |
| 109 | PPMI 1279 | 54.69 | 49.30 | 70.38 | 46.84 | 66.30 | 79.29 | 63.80 | 58.48 | 52.00 | 58.61 | 72.79 | 61.10 |
| 110 | PPMDMGMP 236 | 79.66 | 51.10 | 60.35 | 53.36 | 71.44 | 61.45 | 70.50 | 55.30 | 65.38 | 56.86 | 66.45 | 62.90 |
| 111 | PPMDMDMP 86 | 39.08 | 56.40 | 49.26 | 47.48 | 63.23 | 58.84 | 50.50 | 54.24 | 47.74 | 48.37 | 61.04 | 52.40 |
| 112 | PPMI 1116 | 71.40 | 49.30 | 75.48 | 72.49 | 73.00 | 106.70 | 73.30 | 76.16 | 60.35 | 73.99 | 89.85 | 74.70 |
| 113 | PPMI 1280 | 63.42 | 42.82 | 54.61 | 51.19 | 44.51 | 55.30 | 54.20 | 49.77 | 53.12 | 52.90 | 49.91 | 52.00 |
| 114 | PPMI 1281 | 64.37 | 39.59 | 33.34 | 58.71 | 31.31 | 61.00 | 43.00 | 53.10 | 51.98 | 46.03 | 46.16 | 48.10 |
| 115 | ICMB 92777 | 41.22 | 35.10 | 49.87 | 47.27 | 42.68 | 92.87 | 44.60 | 58.41 | 38.16 | 48.57 | 67.78 | 51.50 |
| **S.No.** | **Genotype** | **Del-14** | **Del-15** | **Jod-14** | **Jod-15** | **DW-14** | **DW-15** | **Y14-M** | **Y15-M** | **Del-M** | **Jod-M** | **DW-M** | **GM** |
| 116 | PPMI 1282 | 69.50 | 51.87 | 60.64 | 41.61 | 49.94 | 54.32 | 60.00 | 49.27 | 60.69 | 51.13 | 52.13 | 54.60 |
| 117 | PPMI 1283 | 53.04 | 70.13 | 32.50 | 80.39 | 42.34 | 90.86 | 42.60 | 80.46 | 61.59 | 56.44 | 66.60 | 61.50 |
| 118 | PPMI 1284 | 60.85 | 52.63 | 58.44 | 79.63 | 64.97 | 70.85 | 61.40 | 67.70 | 56.74 | 69.04 | 67.91 | 64.60 |
| 119 | PPMI 1101 | 84.86 | 89.24 | 50.73 | 67.46 | 70.78 | 82.85 | 68.80 | 79.85 | 87.05 | 59.10 | 76.82 | 74.30 |
| 120 | PPMI 1286 | 79.38 | 35.85 | 44.63 | 43.25 | 71.15 | 83.86 | 65.10 | 54.32 | 57.62 | 43.94 | 77.51 | 59.70 |
| 121 | PPMI 823 | 42.33 | 41.18 | 37.53 | 33.15 | 62.50 | 53.89 | 47.50 | 42.74 | 41.75 | 35.34 | 58.20 | 45.10 |
| 122 | PPMI 1275 | 45.29 | 39.73 | 88.29 | 63.54 | 41.86 | 49.66 | 58.50 | 50.98 | 42.51 | 75.92 | 45.76 | 54.70 |
| 123 | PPMDMGMP 8 | 47.60 | 40.89 | 31.39 | 29.71 | 64.32 | 62.74 | 47.80 | 44.45 | 44.25 | 30.55 | 63.53 | 46.10 |
| 124 | PPMDMGMP 148 | 53.01 | 54.95 | 76.06 | 63.29 | 49.88 | 52.74 | 59.60 | 56.99 | 53.98 | 69.67 | 51.31 | 58.30 |
| 125 | PPMDMGMP 186 | 71.44 | 80.31 | 72.64 | 58.43 | 45.38 | 55.86 | 63.20 | 64.87 | 75.88 | 65.54 | 50.62 | 64.00 |
| 126 | PPMDMDMP 93 | 55.02 | 48.27 | 40.18 | 36.21 | 75.10 | 68.48 | 56.80 | 50.99 | 51.64 | 38.20 | 71.79 | 53.90 |
| 127 | PPMDMDMP 107 | 57.41 | 59.13 | 47.83 | 43.37 | 45.55 | 53.41 | 50.30 | 51.97 | 58.27 | 45.60 | 49.48 | 51.10 |
| 128 | PPMI 1285 | 65.90 | 72.38 | 46.38 | 41.38 | 57.34 | 66.32 | 56.50 | 60.03 | 69.14 | 43.88 | 61.83 | 58.30 |
| 129 | PPMI 1287 | 48.56 | 67.27 | 49.10 | 45.37 | 55.88 | 75.91 | 51.20 | 62.85 | 57.92 | 47.24 | 65.90 | 57.00 |
| 130 | PPMFeZMP 22 | 32.54 | 60.75 | 41.39 | 57.17 | 50.43 | 47.85 | 41.50 | 55.26 | 46.64 | 49.28 | 49.14 | 48.40 |
|  | Minimum | 27.29 | 29.99 | 23.27 | 24.64 | 31.22 | 34.36 | 29.00 | 34.73 | 29.27 | 29.65 | 36.02 | 32.30 |
|  | Maximum | 125.01 | 117.27 | 121.63 | 111.40 | 139.12 | 123.78 | 114.60 | 112.10 | 116.40 | 109.62 | 122.34 | 111.90 |
|  | Mean | 57.52 | 60.44 | 55.73 | 57.81 | 60.71 | 61.49 | 57.99 | 59.92 | 58.98 | 56.77 | 61.10 | 58.95 |
|  | SEm | 1.54 | 1.43 | 1.80 | 1.43 | 1.99 | 1.51 | 1.38 | 1.13 | 1.34 | 1.38 | 1.48 | 1.13 |
|  | SD | 17.51 | 16.32 | 20.55 | 16.36 | 22.63 | 17.24 | 15.72 | 12.85 | 15.32 | 15.78 | 16.91 | 12.93 |

Where, Del-14, Del-15, Jod-14, Jod-15, DW-14, DW-15, Y14-M, Y15-M, Del-M, Jod-M, DW-M and GM are Delhi during 2014, Delhi during 2015, Jodhpur during 2014, Jodhpur during 2015, Dharwad during 2014, Dharwad during 2015, Year 2014 mean, Year 2015 mean, Delhi mean, Jodhpur mean, Dharwad mean and Grand mean.

**TABLE 5 B │ Mean values of grain zinc content across six environments and their six pooled environments (ppm)**

| **S.No.** | **Genotype** | **Del-14** | **Del-15** | **Jod-14** | **Jod-15** | **DW-14** | **DW-15** | **Y14-M** | **Y15-M** | **Del-M** | **Jod-M** | **DW-M** | **GM** |
| --- | --- | --- | --- | --- | --- | --- | --- | --- | --- | --- | --- | --- | --- |
| 1 | PPMI 1102 | 59.10 | 74.38 | 75.20 | 84.36 | 61.80 | 63.82 | 65.40 | 74.19 | 66.72 | 79.78 | 62.81 | 69.77 |
| 2 | 5540B | 27.90 | 29.10 | 26.60 | 29.84 | 24.60 | 21.62 | 26.40 | 26.85 | 28.50 | 28.24 | 23.11 | 26.62 |
| 3 | PPMI 214 | 65.40 | 59.67 | 44.80 | 58.59 | 26.50 | 20.42 | 45.60 | 46.23 | 62.55 | 51.69 | 23.46 | 45.90 |
| 4 | IPC 1657 | 30.20 | 45.20 | 32.20 | 26.44 | 27.10 | 19.12 | 29.80 | 30.25 | 37.72 | 29.32 | 23.11 | 30.05 |
| 5 | PPMI 708 | 83.80 | 78.38 | 79.60 | 67.49 | 68.20 | 64.62 | 77.20 | 70.16 | 81.08 | 73.55 | 66.41 | 73.68 |
| 6 | PPMI 1087 | 33.30 | 45.20 | 58.80 | 49.37 | 26.50 | 28.42 | 39.50 | 41.00 | 39.25 | 54.06 | 27.46 | 40.26 |
| 7 | PPMWGI 99 | 48.80 | 59.80 | 34.90 | 42.34 | 30.30 | 30.52 | 38.00 | 44.22 | 54.28 | 38.63 | 30.41 | 41.11 |
| 8 | J2467 | 36.20 | 40.29 | 29.90 | 39.23 | 32.40 | 31.52 | 32.90 | 37.01 | 38.26 | 34.59 | 31.96 | 34.93 |
| 9 | PPMI 1225 | 55.00 | 67.34 | 53.90 | 63.89 | 32.90 | 26.32 | 47.30 | 52.52 | 61.17 | 58.90 | 29.61 | 49.90 |
| 10 | J2405 | 43.90 | 50.40 | 31.30 | 34.34 | 31.30 | 33.62 | 35.50 | 39.45 | 47.17 | 32.82 | 32.46 | 37.48 |
| 11 | PPMFeZMP 199 | 75.90 | 72.23 | 48.80 | 59.62 | 69.90 | 69.25 | 64.90 | 67.03 | 74.07 | 54.20 | 69.58 | 65.95 |
| 12 | PPMFeZMP 65 | 46.80 | 49.40 | 45.40 | 43.19 | 23.20 | 26.32 | 38.50 | 39.64 | 48.09 | 44.31 | 24.76 | 39.05 |
| 13 | ICTP 8203 Fe | 53.40 | 58.38 | 59.10 | 57.29 | 39.00 | 35.72 | 50.50 | 50.46 | 55.90 | 58.19 | 37.36 | 50.48 |
| 14 | PPMI 1222 | 35.20 | 50.40 | 39.50 | 40.19 | 35.00 | 28.42 | 36.60 | 39.67 | 42.82 | 39.84 | 31.71 | 38.13 |
| 15 | PPMI 683 | 63.40 | 71.15 | 58.60 | 63.69 | 55.90 | 59.73 | 59.30 | 64.86 | 67.28 | 61.13 | 57.82 | 62.07 |
| 16 | PPMI 627 | 42.30 | 37.60 | 43.70 | 40.26 | 26.20 | 21.12 | 37.40 | 32.99 | 39.97 | 41.99 | 23.66 | 35.21 |
| 17 | ICMR06222 | 63.20 | 60.50 | 46.60 | 52.34 | 23.70 | 21.62 | 44.50 | 44.82 | 61.83 | 49.45 | 22.66 | 44.65 |
| 18 | PPMI 660 | 39.90 | 37.61 | 32.40 | 39.27 | 29.50 | 38.58 | 33.90 | 38.49 | 38.74 | 35.84 | 34.04 | 36.21 |
| 19 | PIB 228 | 48.50 | 54.60 | 81.10 | 72.39 | 27.70 | 30.52 | 52.50 | 52.50 | 51.56 | 76.76 | 29.11 | 52.48 |
| 20 | PPMWGI 146 | 33.20 | 36.43 | 31.00 | 38.26 | 34.30 | 24.22 | 32.80 | 32.97 | 34.79 | 34.63 | 29.26 | 32.89 |
| 21 | PPMI 295 | 46.10 | 39.00 | 86.00 | 77.39 | 25.80 | 20.12 | 52.60 | 45.50 | 42.55 | 81.71 | 22.96 | 49.08 |
| 22 | PPMI 1220 | 34.60 | 42.34 | 28.90 | 42.36 | 32.90 | 22.02 | 32.10 | 35.57 | 38.48 | 35.61 | 27.46 | 33.85 |
| 23 | PPMFeZMP 37 | 51.10 | 50.40 | 43.10 | 40.21 | 47.00 | 44.02 | 47.10 | 44.88 | 50.75 | 41.67 | 45.51 | 45.98 |
| 24 | J108 | 46.60 | 29.60 | 36.50 | 35.63 | 21.70 | 20.32 | 34.90 | 28.52 | 38.09 | 36.05 | 21.01 | 31.72 |
| 25 | PPMI 85 | 46.70 | 37.82 | 37.30 | 40.22 | 28.50 | 23.22 | 37.50 | 33.75 | 42.24 | 38.75 | 25.86 | 35.62 |
| **S.No.** | **Genotype** | **Del-14** | **Del-15** | **Jod-14** | **Jod-15** | **DW-14** | **DW-15** | **Y14-M** | **Y15-M** | **Del-M** | **Jod-M** | **DW-M** | **GM** |
| 26 | PPMI 301 | 55.60 | 55.95 | 60.40 | 59.63 | 35.30 | 38.82 | 50.40 | 51.47 | 55.76 | 60.01 | 37.06 | 50.95 |
| 27 | ICMB 98222 | 57.90 | 64.92 | 38.10 | 43.89 | 48.23 | 55.52 | 48.10 | 54.78 | 61.42 | 41.01 | 51.88 | 51.43 |
| 28 | D 23 | 41.40 | 44.59 | 35.80 | 35.64 | 36.00 | 35.72 | 37.70 | 38.65 | 43.00 | 35.72 | 35.86 | 38.19 |
| 29 | PPMI 1103 | 48.20 | 48.32 | 37.50 | 52.53 | 47.40 | 62.82 | 44.40 | 54.56 | 48.25 | 45.03 | 55.11 | 49.46 |
| 30 | PPMI 1104 | 59.90 | 48.10 | 40.50 | 48.29 | 73.27 | 68.02 | 57.90 | 54.80 | 54.01 | 44.38 | 70.65 | 56.35 |
| 31 | PPMI 1105 | 45.20 | 52.21 | 40.60 | 49.23 | 64.39 | 74.22 | 50.10 | 58.55 | 48.68 | 44.94 | 69.31 | 54.31 |
| 32 | PPMI 1107 | 60.90 | 70.32 | 44.60 | 60.28 | 52.37 | 61.72 | 52.60 | 64.11 | 65.60 | 52.44 | 57.05 | 58.36 |
| 33 | PPMI 1108 | 64.60 | 54.89 | 53.40 | 65.37 | 57.29 | 46.12 | 58.40 | 55.46 | 59.74 | 59.39 | 51.71 | 56.95 |
| 34 | PPMI 1112 | 42.10 | 52.73 | 70.60 | 64.99 | 38.64 | 53.42 | 50.40 | 57.05 | 47.40 | 67.81 | 46.03 | 53.75 |
| 35 | 841B | 52.10 | 50.40 | 42.50 | 57.23 | 44.19 | 52.32 | 46.30 | 53.32 | 51.27 | 49.85 | 48.26 | 49.79 |
| 36 | 5141B | 43.50 | 50.93 | 37.90 | 47.63 | 35.10 | 44.02 | 38.80 | 47.53 | 47.22 | 42.74 | 39.56 | 43.17 |
| 37 | 5054B | 49.00 | 48.30 | 24.40 | 34.65 | 35.80 | 33.62 | 36.40 | 38.86 | 48.67 | 29.54 | 34.71 | 37.64 |
| 38 | 6030B | 38.70 | 48.40 | 31.60 | 29.84 | 40.40 | 40.92 | 36.90 | 39.72 | 43.56 | 30.73 | 40.66 | 38.32 |
| 39 | 351B | 45.70 | 54.72 | 44.80 | 55.28 | 28.30 | 30.52 | 39.60 | 46.84 | 50.21 | 50.06 | 29.41 | 43.23 |
| 40 | 379B | 51.40 | 42.02 | 50.90 | 40.99 | 32.90 | 36.72 | 45.10 | 39.91 | 46.72 | 45.96 | 34.81 | 42.50 |
| 41 | 411B | 37.70 | 42.94 | 33.10 | 36.89 | 25.60 | 26.32 | 32.10 | 35.38 | 40.32 | 35.00 | 25.96 | 33.76 |
| 42 | 576B | 37.00 | 52.50 | 34.20 | 44.19 | 34.80 | 30.52 | 35.30 | 42.40 | 44.73 | 39.21 | 32.66 | 38.87 |
| 43 | PPMI 59 | 39.40 | 47.30 | 36.20 | 42.09 | 37.64 | 22.22 | 37.70 | 37.20 | 43.34 | 39.13 | 29.93 | 37.46 |
| 44 | PPMI 265 | 86.20 | 72.50 | 36.10 | 36.89 | 34.30 | 28.42 | 52.20 | 45.94 | 79.34 | 36.47 | 31.36 | 49.06 |
| 45 | PPMI 269 | 35.30 | 43.73 | 32.80 | 45.19 | 38.30 | 30.52 | 35.50 | 39.81 | 39.52 | 39.02 | 34.41 | 37.65 |
| 46 | PPMI 275 | 50.40 | 58.64 | 52.20 | 44.15 | 41.70 | 36.72 | 48.10 | 46.50 | 54.51 | 48.16 | 39.21 | 47.29 |
| 47 | PPMI 496 | 41.40 | 49.40 | 30.40 | 42.14 | 32.10 | 23.22 | 34.60 | 38.25 | 45.38 | 36.25 | 27.66 | 36.43 |
| 48 | PPMI 694 | 54.70 | 60.80 | 32.70 | 49.37 | 28.10 | 40.92 | 38.50 | 50.36 | 57.73 | 41.05 | 34.51 | 44.43 |
| 49 | PPMI 719 | 42.50 | 53.21 | 35.00 | 51.49 | 32.50 | 21.62 | 36.70 | 42.11 | 47.85 | 43.26 | 27.06 | 39.39 |
| 50 | PPMI 744 | 48.40 | 57.61 | 29.20 | 38.26 | 32.60 | 19.42 | 36.70 | 38.43 | 53.01 | 33.74 | 26.01 | 37.59 |
| 51 | PPMI 759 | 35.70 | 48.30 | 44.40 | 30.69 | 27.00 | 20.32 | 35.70 | 33.10 | 42.01 | 37.56 | 23.66 | 34.41 |
| 52 | EGPN 423 | 42.20 | 57.70 | 36.20 | 24.39 | 30.70 | 24.22 | 36.40 | 35.44 | 49.95 | 30.27 | 27.46 | 35.89 |
| 53 | PPMWGI 152 | 43.50 | 59.71 | 40.10 | 49.39 | 36.80 | 21.12 | 40.20 | 43.41 | 51.63 | 44.75 | 28.96 | 41.78 |
| 54 | PPMWGI 100 | 40.70 | 46.30 | 34.20 | 32.84 | 36.15 | 47.12 | 37.00 | 42.09 | 43.51 | 33.53 | 41.64 | 39.56 |
| **S.No.** | **Genotype** | **Del-14** | **Del-15** | **Jod-14** | **Jod-15** | **DW-14** | **DW-15** | **Y14-M** | **Y15-M** | **Del-M** | **Jod-M** | **DW-M** | **GM** |
| 55 | PPMWGI 108 | 57.00 | 49.83 | 34.50 | 22.29 | 32.20 | 23.42 | 41.20 | 31.85 | 53.43 | 28.39 | 27.81 | 36.54 |
| 56 | KSMWGI 14 | 42.80 | 39.00 | 56.70 | 51.49 | 34.30 | 31.52 | 44.60 | 40.67 | 40.91 | 54.10 | 32.91 | 42.64 |
| 57 | PPMI 1224 | 47.20 | 40.00 | 62.10 | 49.39 | 34.59 | 21.42 | 48.00 | 36.94 | 43.60 | 55.75 | 28.01 | 42.45 |
| 58 | PPMI 1231 | 33.20 | 39.76 | 27.70 | 37.26 | 33.70 | 38.82 | 31.50 | 38.61 | 36.50 | 32.47 | 36.26 | 35.08 |
| 59 | PPMI 1233 | 62.90 | 69.20 | 41.60 | 59.37 | 24.90 | 20.12 | 43.10 | 49.56 | 66.04 | 50.51 | 22.51 | 46.35 |
| 60 | PPMI 1263 | 44.90 | 61.40 | 25.70 | 38.64 | 47.34 | 31.52 | 39.30 | 43.85 | 53.15 | 32.17 | 39.43 | 41.58 |
| 61 | ICMR07111 | 41.00 | 39.00 | 39.80 | 32.69 | 25.80 | 31.52 | 35.60 | 34.40 | 40.00 | 36.27 | 28.66 | 34.98 |
| 62 | ICMR07999 | 38.70 | 27.50 | 73.70 | 68.94 | 33.50 | 28.42 | 48.60 | 41.62 | 33.10 | 71.31 | 30.96 | 45.12 |
| 63 | PPMI 1084 | 64.80 | 61.90 | 38.70 | 56.23 | 32.60 | 37.82 | 45.40 | 51.98 | 63.37 | 47.45 | 35.21 | 48.68 |
| 64 | PPMI 1086 | 35.70 | 49.37 | 58.90 | 38.99 | 38.40 | 27.42 | 44.30 | 38.59 | 42.54 | 48.92 | 32.91 | 41.46 |
| 65 | PPMI 1089 | 61.90 | 53.50 | 39.90 | 34.79 | 37.61 | 46.12 | 46.50 | 44.80 | 57.72 | 37.34 | 41.87 | 45.64 |
| 66 | PPMI 1090 | 34.30 | 25.40 | 48.60 | 40.19 | 32.90 | 33.62 | 38.60 | 33.07 | 29.84 | 44.41 | 33.26 | 35.83 |
| 67 | PPMI 1092 | 29.60 | 39.00 | 50.80 | 26.59 | 36.60 | 30.52 | 39.00 | 32.04 | 34.31 | 38.72 | 33.56 | 35.53 |
| 68 | PPMI 1155 | 46.10 | 31.90 | 28.40 | 27.59 | 25.50 | 20.42 | 33.30 | 26.64 | 39.00 | 27.97 | 22.96 | 29.98 |
| 69 | PPMI 1161 | 39.70 | 55.60 | 40.50 | 49.09 | 41.90 | 30.52 | 40.70 | 45.07 | 47.63 | 44.78 | 36.21 | 42.87 |
| 70 | PPMI 1165 | 48.60 | 43.10 | 40.80 | 49.09 | 31.60 | 43.02 | 40.30 | 45.07 | 45.86 | 44.94 | 37.31 | 42.70 |
| 71 | PIB 686 | 48.20 | 49.40 | 30.20 | 48.09 | 28.90 | 33.62 | 35.80 | 43.70 | 48.81 | 39.13 | 31.26 | 39.73 |
| 72 | HBL 11 | 34.30 | 37.90 | 20.30 | 34.39 | 25.30 | 24.22 | 26.70 | 32.17 | 36.12 | 27.34 | 24.76 | 29.41 |
| 73 | H77/833-2-202 | 54.00 | 57.40 | 32.40 | 47.64 | 40.70 | 29.42 | 42.40 | 44.82 | 55.69 | 40.01 | 35.06 | 43.59 |
| 74 | H77/833-2 | 40.80 | 41.10 | 42.00 | 42.19 | 32.60 | 30.52 | 38.50 | 37.94 | 40.94 | 42.11 | 31.56 | 38.20 |
| 75 | J2454 | 41.60 | 60.80 | 37.20 | 47.09 | 23.70 | 37.82 | 34.20 | 48.57 | 51.19 | 42.15 | 30.76 | 41.37 |
| 76 | J2496 | 40.60 | 41.10 | 32.20 | 38.29 | 34.90 | 21.12 | 35.90 | 33.50 | 40.84 | 35.26 | 28.01 | 34.70 |
| 77 | IPC 1480 | 30.10 | 40.00 | 33.20 | 34.39 | 27.90 | 20.02 | 30.40 | 31.47 | 35.03 | 33.78 | 23.96 | 30.92 |
| 78 | PPMFeZMP 72 | 40.80 | 26.50 | 30.10 | 39.29 | 30.40 | 36.72 | 33.80 | 34.17 | 33.64 | 34.71 | 33.56 | 33.97 |
| 79 | PPMI 1218 | 45.40 | 29.60 | 54.70 | 59.35 | 32.10 | 19.42 | 44.10 | 36.12 | 37.48 | 57.05 | 25.76 | 40.09 |
| 80 | PPMI 1267 | 57.60 | 49.40 | 53.00 | 52.99 | 35.68 | 27.42 | 48.70 | 43.27 | 53.48 | 52.97 | 31.55 | 46.00 |
| 81 | PPMI 1268 | 34.10 | 47.30 | 28.00 | 47.09 | 37.50 | 29.42 | 33.20 | 41.27 | 40.69 | 37.55 | 33.46 | 37.23 |
| 82 | PPMI 1269 | 44.40 | 54.60 | 44.30 | 56.89 | 35.00 | 40.92 | 41.20 | 50.80 | 49.50 | 50.60 | 37.96 | 46.02 |
| 83 | PPMFeZMP 34 | 37.20 | 50.40 | 55.00 | 56.89 | 31.20 | 39.92 | 41.10 | 49.07 | 43.82 | 55.92 | 35.56 | 45.10 |
| **S.No.** | **Genotype** | **Del-14** | **Del-15** | **Jod-14** | **Jod-15** | **DW-14** | **DW-15** | **Y14-M** | **Y15-M** | **Del-M** | **Jod-M** | **DW-M** | **GM** |
| 84 | PPMFeZMP 125 | 45.70 | 55.40 | 62.10 | 55.89 | 33.40 | 21.42 | 47.10 | 44.24 | 50.53 | 59.01 | 27.41 | 45.65 |
| 85 | PPMFeZMP 126 | 50.70 | 67.91 | 74.50 | 69.24 | 39.60 | 25.32 | 54.90 | 54.16 | 59.29 | 71.89 | 32.46 | 54.55 |
| 86 | PPMFeZMP 153 | 37.00 | 50.40 | 66.60 | 51.99 | 31.90 | 38.82 | 45.20 | 47.07 | 43.71 | 59.31 | 35.36 | 46.13 |
| 87 | HTP 94/54 | 39.30 | 30.71 | 33.20 | 29.49 | 32.40 | 30.52 | 34.90 | 30.24 | 35.00 | 31.32 | 31.46 | 32.59 |
| 88 | PPMI 1270 | 43.60 | 52.50 | 52.80 | 38.29 | 27.70 | 22.22 | 41.30 | 37.67 | 48.04 | 45.52 | 24.96 | 39.51 |
| 89 | PPMI 1271 | 33.90 | 32.70 | 45.70 | 58.42 | 24.90 | 20.12 | 34.80 | 37.08 | 33.28 | 52.08 | 22.51 | 35.96 |
| 90 | PPMI 1272 | 40.20 | 36.90 | 49.40 | 49.09 | 26.40 | 21.12 | 38.70 | 35.70 | 38.54 | 49.23 | 23.76 | 37.18 |
| 91 | PPMFeZMP 30 | 37.00 | 23.40 | 57.80 | 44.39 | 26.50 | 19.52 | 40.40 | 29.10 | 30.21 | 51.09 | 23.01 | 34.77 |
| 92 | PPMFeZMP 35 | 58.70 | 73.15 | 47.60 | 58.79 | 32.20 | 20.62 | 46.20 | 50.85 | 65.94 | 53.21 | 26.41 | 48.52 |
| 93 | PPMFeZMP 47 | 38.00 | 54.60 | 29.00 | 44.19 | 31.90 | 45.12 | 33.00 | 47.97 | 46.30 | 36.57 | 38.51 | 40.46 |
| 94 | PPMFeZMP 87 | 42.60 | 41.10 | 24.60 | 32.49 | 39.70 | 44.02 | 35.70 | 39.20 | 41.87 | 28.55 | 41.86 | 37.43 |
| 95 | PPMI 1273 | 48.10 | 57.36 | 33.70 | 49.99 | 24.00 | 47.12 | 35.30 | 51.49 | 52.75 | 41.84 | 35.56 | 43.38 |
| 96 | PPMI 1274 | 44.60 | 51.49 | 38.30 | 56.14 | 33.50 | 62.82 | 38.80 | 56.82 | 48.07 | 47.23 | 48.16 | 47.82 |
| 97 | PPMFeZMP 143 | 42.50 | 60.93 | 43.50 | 49.09 | 28.00 | 40.92 | 38.00 | 50.31 | 51.71 | 46.29 | 34.46 | 44.15 |
| 98 | G73-107 | 35.60 | 32.70 | 32.60 | 50.99 | 31.40 | 35.72 | 33.20 | 39.80 | 34.14 | 41.80 | 33.56 | 36.50 |
| 99 | PPMI 1067 | 39.10 | 50.92 | 58.40 | 58.79 | 61.30 | 58.62 | 52.90 | 56.11 | 45.01 | 58.57 | 59.96 | 54.51 |
| 100 | PPMI 1276 | 54.70 | 59.80 | 42.60 | 52.37 | 34.67 | 47.12 | 44.00 | 53.10 | 57.24 | 47.48 | 40.90 | 48.54 |
| 101 | PPMDMGMP 27 | 37.10 | 54.96 | 41.60 | 54.61 | 42.19 | 51.32 | 40.30 | 53.63 | 46.01 | 48.09 | 46.76 | 46.95 |
| 102 | PPMDMGMP 60 | 69.90 | 74.16 | 55.70 | 66.59 | 63.50 | 50.32 | 63.00 | 63.69 | 72.03 | 61.15 | 56.91 | 63.36 |
| 103 | PPMDMGMP 87 | 41.50 | 48.30 | 32.60 | 35.76 | 37.61 | 45.12 | 37.30 | 43.06 | 44.92 | 34.20 | 41.37 | 40.16 |
| 104 | PPMDMGMP 99 | 46.60 | 59.80 | 52.00 | 47.09 | 44.80 | 48.22 | 47.80 | 51.70 | 53.22 | 49.56 | 46.51 | 49.76 |
| 105 | PPMI 1277 | 48.00 | 45.20 | 29.00 | 40.29 | 31.20 | 36.72 | 36.10 | 40.74 | 46.62 | 34.66 | 33.96 | 38.41 |
| 106 | PPMI 1278 | 53.00 | 61.34 | 40.90 | 29.49 | 39.60 | 36.72 | 44.50 | 42.52 | 57.15 | 35.18 | 38.16 | 43.50 |
| 107 | PPMI 1011 | 56.00 | 50.40 | 38.30 | 45.36 | 34.30 | 21.12 | 42.90 | 38.96 | 53.22 | 41.83 | 27.71 | 40.92 |
| 108 | ICMB 04222 | 58.60 | 60.80 | 41.80 | 27.59 | 52.20 | 40.92 | 50.90 | 43.10 | 59.70 | 34.69 | 46.56 | 46.98 |
| 109 | PPMI 1279 | 47.70 | 41.10 | 43.50 | 26.59 | 36.00 | 44.02 | 42.40 | 37.24 | 44.39 | 35.04 | 40.01 | 39.81 |
| 110 | PPMDMGMP 236 | 51.90 | 35.90 | 41.70 | 31.49 | 51.70 | 39.92 | 48.40 | 35.77 | 43.88 | 36.57 | 45.81 | 42.08 |
| 111 | PPMDMDMP 86 | 34.70 | 42.10 | 35.70 | 30.29 | 32.60 | 37.82 | 34.30 | 36.74 | 38.41 | 33.00 | 35.21 | 35.54 |
| 112 | PPMI 1116 | 71.10 | 62.90 | 64.30 | 58.22 | 66.10 | 69.02 | 67.10 | 63.38 | 67.00 | 61.24 | 67.56 | 65.26 |
| **S.No.** | **Genotype** | **Del-14** | **Del-15** | **Jod-14** | **Jod-15** | **DW-14** | **DW-15** | **Y14-M** | **Y15-M** | **Del-M** | **Jod-M** | **DW-M** | **GM** |
| 113 | PPMI 1280 | 47.30 | 40.39 | 43.80 | 36.39 | 27.30 | 34.72 | 39.50 | 37.17 | 43.85 | 40.09 | 31.01 | 38.32 |
| 114 | PPMI 1281 | 32.70 | 31.70 | 31.40 | 48.09 | 39.61 | 41.92 | 34.60 | 40.57 | 32.18 | 39.76 | 40.77 | 37.57 |
| 115 | ICMB 92777 | 35.10 | 32.71 | 38.80 | 36.39 | 38.30 | 39.92 | 37.40 | 36.34 | 33.90 | 37.59 | 39.11 | 36.87 |
| 116 | PPMI 1282 | 58.00 | 46.90 | 52.30 | 49.87 | 36.10 | 39.92 | 48.80 | 45.56 | 52.47 | 51.09 | 38.01 | 47.19 |
| 117 | PPMI 1283 | 40.00 | 54.61 | 25.80 | 38.63 | 58.94 | 60.72 | 41.60 | 51.32 | 47.33 | 32.20 | 59.83 | 46.45 |
| 118 | PPMI 1284 | 43.90 | 39.00 | 33.80 | 49.12 | 32.20 | 46.12 | 36.60 | 44.75 | 41.44 | 41.45 | 39.16 | 40.68 |
| 119 | PPMI 1101 | 68.70 | 75.99 | 42.40 | 43.99 | 63.80 | 54.42 | 58.30 | 58.13 | 72.34 | 43.21 | 59.11 | 58.22 |
| 120 | PPMI 1286 | 35.90 | 28.20 | 42.80 | 31.68 | 44.23 | 51.32 | 41.00 | 37.07 | 32.05 | 37.23 | 47.78 | 39.02 |
| 121 | PPMI 823 | 34.50 | 30.65 | 24.30 | 25.39 | 22.90 | 38.18 | 27.20 | 31.41 | 32.57 | 24.85 | 30.54 | 29.32 |
| 122 | PPMI 1275 | 40.70 | 28.50 | 50.60 | 49.71 | 32.60 | 24.38 | 41.30 | 34.20 | 34.60 | 50.13 | 28.49 | 37.74 |
| 123 | PPMDMGMP 8 | 34.20 | 28.36 | 24.80 | 32.13 | 28.40 | 24.33 | 29.20 | 28.27 | 31.29 | 28.48 | 26.37 | 28.71 |
| 124 | PPMDMGMP 148 | 38.60 | 39.70 | 33.80 | 37.34 | 35.30 | 32.12 | 35.90 | 36.39 | 39.13 | 35.56 | 33.71 | 36.13 |
| 125 | PPMDMGMP 186 | 80.70 | 67.20 | 52.50 | 42.16 | 27.00 | 23.42 | 53.40 | 44.26 | 73.97 | 47.32 | 25.21 | 48.83 |
| 126 | PPMDMDMP 93 | 37.70 | 37.35 | 29.00 | 29.70 | 24.10 | 26.15 | 30.30 | 31.07 | 37.52 | 29.36 | 25.13 | 30.67 |
| 127 | PPMDMDMP 107 | 37.50 | 35.70 | 43.90 | 42.22 | 35.50 | 29.81 | 39.00 | 35.91 | 36.62 | 43.06 | 32.66 | 37.45 |
| 128 | PPMI 1285 | 83.70 | 76.95 | 49.40 | 28.15 | 38.60 | 29.36 | 57.20 | 44.82 | 80.32 | 38.76 | 33.98 | 51.02 |
| 129 | PPMI 1287 | 45.60 | 61.31 | 49.50 | 35.39 | 43.29 | 55.52 | 46.10 | 50.74 | 53.46 | 42.46 | 49.41 | 48.44 |
| 130 | PPMFeZMP 22 | 37.40 | 27.59 | 32.00 | 33.23 | 33.40 | 32.82 | 34.30 | 31.21 | 32.50 | 32.61 | 33.11 | 32.74 |
|  | Minimum | 27.90 | 23.40 | 20.30 | 22.29 | 21.70 | 19.12 | 26.40 | 26.64 | 28.50 | 24.85 | 21.01 | 26.62 |
|  | Maximum | 86.20 | 78.38 | 86.00 | 84.36 | 73.27 | 74.22 | 77.20 | 74.19 | 81.08 | 81.71 | 70.65 | 73.68 |
|  | Mean | 46.61 | 49.06 | 42.75 | 45.16 | 36.40 | 35.74 | 41.92 | 43.32 | 47.83 | 43.96 | 36.07 | 42.62 |
|  | SEm | 1.04 | 1.11 | 1.14 | 1.05 | 0.95 | 1.16 | 0.78 | 0.83 | 1.00 | 1.01 | 0.99 | 0.76 |
|  | SD | 11.81 | 12.75 | 13.00 | 12.04 | 10.86 | 13.29 | 8.84 | 9.44 | 11.39 | 11.48 | 11.33 | 8.62 |

Where, Del-14, Del-15, Jod-14, Jod-15, DW-14, DW-15, Y14-M, Y15-M, Del-M, Jod-M, DW-M and GM are Delhi during 2014, Delhi during 2015, Jodhpur during 2014, Jodhpur during 2015, Dharwad during 2014, Dharwad during 2015, Year 2014 mean, Year 2015 mean, Delhi mean, Jodhpur mean, Dharwad mean and Grand mean. SEM, SD are standard error mean and standard deviation
