# Supplementary material for: Deciphering Genomic Regions for High Grain Iron and Zinc Content Using Association Mapping in Pearl Millet
Source: Front Plant Sci. 2017 May 1;8:412. doi: 10.3389/fpls.2017.00412 (PMC5410614; doi:10.3389/fpls.2017.00412)
Supplement: Table S6 — Correlation between grain iron and zinc content and population structure using multiple regression analysis. [file Table6.DOCX]

**TABLE S 6│ Correlation between grain iron and zinc content and population structure using multiple regression analysis.**

| **Environment** | **Fe** | | **Zn** | |
| --- | --- | --- | --- | --- |
|  | **R^2^** | **p-value** | **R^2^** | **p-value** |
| Del-14 | 0.0025 | 0.852 | 0.001 | 0.97 |
| Jod-14 | 0.0297 | 0.148 | 0.016 | 0.348 |
| DW-14 | 0.009 | 0.572 | 0.059 | 0.022 |
| Del-15 | 0.01 | 0.4 | 0.003 | 0.821 |
| Jod-15 | 0.001 | 0.963 | 0.024 | 0.217214 |
| DW-15 | 0.057 | 0.025 | 0.139 | 7.66E-05 |
| Y14-M | 0.015 | 0.378 | 0.001 | 0.933 |
| Y15-M | 0.004 | 0.763 | 0.009 | 0.577 |
| Del-M | 0.004 | 0.753 | 0.002 | 0.857 |
| Jod-M | 0.012 | 0.462 | 0.023 | 0.222 |
| DW-M | 0.004 | 0.781 | 0.111 | 0.000574 |
| GM | 0.004 | 0.779 | 0.005 | 0.749 |
