# Supplementary material for: Deciphering Genomic Regions for High Grain Iron and Zinc Content Using Association Mapping in Pearl Millet
Source: Front Plant Sci. 2017 May 1;8:412. doi: 10.3389/fpls.2017.00412 (PMC5410614; doi:10.3389/fpls.2017.00412)
Supplement: Table S7A — Marker wise associations observed across all datasets for grain iron content in MLM at p < 0.0037. [file Table7.docx]

**TABLE S 7A │Marker wise associations observed across all datasets for grain iron content in MLM at p < 0.00375**

| **LG** | **Marker** | **Trait** | **marker_p** | **Marker R^2^** |
| --- | --- | --- | --- | --- |
| 3 | *Xipes* 0180 | GM | 9.89 x 10^-4^ | 0.131 |
| 3 | *Xipes* 0180 | Del-14 | 0.0017 | 0.124 |
|  | *Xipes* 0180 | Del-M | 0.0036 | 0.102 |
| 3 | *Xipes* 0180 | Y14-M | 0.0024 | 0.118 |
| 5 | *Xpsmp* 2261 | Del-15 | 6.00 x 10^-4^ | 0.122 |
| 5 | *Xpsmp* 2261 | Del-M | 0.0034 | 0.092 |
| 5 | *Xpsmp* 2261 | GM | 1.7 x 10^-4^ | 0.144 |
| 5 | *Xpsmp* 2261 | Jod-14 | 8.1 x 10^-5^ | 0.157 |
| 5 | *Xpsmp* 2261 | Jod-15 | 6.44 x 10^-4^ | 0.121 |
| 5 | *Xpsmp* 2261 | Jod-M | 2.22 x 10^-5^ | 0.181 |
| 5 | *Xpsmp* 2261 | Y14-M | 3.28 x 10^-4^ | 0.133 |
| 5 | *Xpsmp* 2261 | Y15-M | 2.17 x 10^-4^ | 0.140 |
| 7 | *Xicmp*3092 | DW-14 | 0.0036 | 0.091 |
| 7 | *Xipes* 0096 | Del-M | 0.0012 | 0.110 |
| 7 | *Xipes* 0096 | GM | 1.36 x 10^-4^ | 0.148 |
| 7 | *Xipes* 0096 | Jod-14 | 0.0024 | 0.098 |
| 7 | *Xipes* 0096 | Y14-M | 7.42 x 10^-4^ | 0.118 |
| 7 | *Xipes* 0096 | Y15-M | 0.0017 | 0.104 |
| - | *Xpsmp* 2209 | DW-15 | 0.0034 | 0.070 |
| - | *Xsinramp* 6 | Del-14 | 0.0036 | 0.101 |
| - | *Xsinramp* 6 | Del-M | 0.0033 | 0.098 |

**TABLE S 7B │ Marker wise associations observed across all datasets for grain zinc content in MLM at p < 0.00375**

| **LG** | **Marker** | **Trait** | **Marker_p** | **Marker R^2^** |
| --- | --- | --- | --- | --- |
| 3 | *Xipes* 0180 | Jod-15 | 0.0032 | 0.095 |
| 4 | *Xpsmp* 2086 | DW-14 | 0.0015 | 0.108 |
| 5 | *Xpsmp* 2261 | Jod-M | 5.35 x 10^-5^ | 0.165 |
| 5 | *Xpsmp* 2261 | Jod-15 | 6.14 x 10^-5^ | 0.162 |
| 5 | *Xpsmp* 2261 | Jod-14 | 3.61 x 10^-4^ | 0.131 |
| 5 | *Xpsmp* 2261 | Y 14-M | 2.11 x 10^-4^ | 0.140 |
| 5 | *Xpsmp* 2261 | GM | 3.00 x 10^-4^ | 0.134 |
| 5 | *Xpsmp* 2261 | Del-14 | 0.0021 | 0.106 |
| 5 | *Xpsmp* 2261 | Del-M | 0.0031 | 0.099 |
| 5 | *Xpsmp* 2261 | Y 15-M | 0.0014 | 0.107 |
| 6 | *Xipes* 0224 | Jod-14 | 0.0017 | 0.105 |
| 6 | *Xpsmp* 2213 | Jod-M | 2.09 x 10^-4^ | 0.141 |
| 7 | *Xipes* 0096 | Y 14-M | 6.43 x 10^-4^ | 0.121 |
| 7 | *Xipes* 0096 | GM | 9.41 x 10^-4^ | 0.114 |
| 7 | *Xipes* 0096 | Dw-M | 0.0030 | 0.094 |
| 7 | *Xipes* 0096 | DW-14 | 4.56 x 10^-4^ | 0.127 |
| 7 | *Xipes* 0096 | Y 15-M | 0.0017 | 0.104 |
| - | *Xicmp* 4006 | DW-15 | 0.0017 | 0.104 |
| - | *Xicmp* 3004 | Jod-14 | 0.0023 | 0.103 |
| - | *Xicmp* 3016 | Del-15 | 0.0032 | 0.082 |
| - | *Xsinramp* 6 | DW-14 | 0.0020 | 0.107 |
| - | *Xsinramp* 6 | Dw-M | 6.17 x 10^-4^ | 0.130 |
| - | *Xsinramp* 6 | DW-15 | 0.0012 | 0.119 |
