# Supplementary material for: Deciphering Genomic Regions for High Grain Iron and Zinc Content Using Association Mapping in Pearl Millet
Source: Front Plant Sci. 2017 May 1;8:412. doi: 10.3389/fpls.2017.00412 (PMC5410614; doi:10.3389/fpls.2017.00412)
Supplement: Table S8 — Linkage group wise number of significant markers associated with grain iron and zinc content. [file Table8.docx]

**TABLE S 8│ Linkage group wise number of significant markers associated with grain iron and zinc content in MLM**

|  | **Fe** | | **Zn** | | **Fe and Zn together** | |
| --- | --- | --- | --- | --- | --- | --- |
| **LG** | **No. of markers** | **Marker name** | **No. of markers** | **Marker name** | **No. of markers** | **Marker name** |
| 3 | 1 | *Xipes* 0180 | 1 | *Xipes* 0180 | 1 | *Xipes* 0180 |
| 4 | nil | ns | 1 | *Xpsmp* 2086 | 1 | *Xpsmp* 2086 |
| 5 | 1 | *Xpsmp* 2261 | 1 | *Xpsmp* 2261 | 1 | *Xpsmp* 2261 |
| 6 | nil | ns | 2 | *Xipes* 0224 *Xpsmp* 2213 | 2 | *Xipes* 0224 *Xpsmp* 2213 |
| 7 | 2 | *Xicmp*  3092  *Xipes* 0096 | 1 | *Xipes* 0096 | 2 | *Xicmp*  3092  *Xipes* 0096 |
| Markers not mapped | 2 | *Xpsmp* 2209  *Xsinramp* 6 | 4 | *Xicmp* 3004  *Xicmp* 3016  *Xicmp* 4006  *Xsinramp* 6 | 5 | *Xicmp* 3004  *Xicmp* 3016  *Xicmp* 4006  *Xpsmp* 2209  *Xsinramp* 6 |
| **TOTAL** | **6** |  | **10** |  | **12** |  |
