# Supplementary material for: Deciphering Genomic Regions for High Grain Iron and Zinc Content Using Association Mapping in Pearl Millet
Source: Front Plant Sci. 2017 May 1;8:412. doi: 10.3389/fpls.2017.00412 (PMC5410614; doi:10.3389/fpls.2017.00412)
Supplement: Table S9A — Markers associated significantly (p = 0.05) with grain iron content at sub-population level. [file Table9.docx]

**TABLE S 9A│Markers associated significantly (p = 0.05) with grain iron content at sub-population level.** (Associations were tested through one way ANOVA of significant MTAs obtained in MLM for grain iron content).

| **Environment** | **Sub-population** | **Associated markers** |
| --- | --- | --- |
| Del-14 | A | *Xipes* 0180, *Xsinramp* 6 |
|  | B | *Xipes* 0096, *Xpsmp* 2261 |
|  | C | *Xipes* 0180, *Xpsmp* 2261, *Xsinramp* 6 |
| Del 15 | A | *Xipes* 0180, *Xpsmp* 2261 |
|  | B | *Xipes* 0096, *Xpsmp* 2261, *Xsinramp* 6 |
|  | C | *Xicmp*3092, *Xpsmp* 2261, *Xsinramp* 6 |
| Del M | A | *Xsinramp* 6 |
|  | B | *Xipes* 0096, *Xpsmp* 2261, *Xsinramp* 6 |
|  | C | *Xipes* 0180, *Xpsmp* 2261, *Xsinramp* 6 |
| DW-14 | A | *Xicmp*3092 |
|  | B | *Xipes* 0096, *Xpsmp* 2261 |
|  | C | *Xicmp*3092 |
| DW-15 | A | *Xsinramp* 6 |
|  | B | *Xpsmp* 2261, *Xipes* 0096 |
|  | C | *Xpsmp* 2209 |
| DW-M | A | *Xsinramp* 6 |
|  | B | *Xpsmp* 2261, *Xipes* 0096 |
|  | C | *Xipes* 0096 |
| Jod-14 | A | *ns |
|  | B | *Xipes* 0096, *Xpsmp* 2261 |
|  | C | *Xicmp*3092 |
| Jod-15 | A | ns |
|  | B | *Xipes* 0096, *Xpsmp* 2261 |
|  | C | *Xipes* 0180 |
| Jod-M | A | ns |
|  | B | *Xipes* 0096, *Xpsmp* 2261 |
|  | C | *Xicmp*3092, *Xpsmp* 2261 |
| Y14-M | A | *Xicmp*3092 |
|  | B | *Xipes* 0096, *Xpsmp* 2261 |
|  | C | *Xpsmp* 2261, *Xipes* 0180 |
| Y15-M | A | *Xsinramp* 6, *Xipes* 0180 |
|  | B | *Xipes* 0096, *Xpsmp* 2261 |
|  | C | *Xsinramp* 6, *Xpsmp* 2261, *Xipes* 0180 |
| GM | A | *Xipes* 0180, *Xsinramp* 6 |
|  | B | *Xipes* 0096, *Xpsmp* 2261 |
|  | C | *Xipes* 0180, *Xpsmp* 2261 |

*ns means non-significant. Sub-populations are as per Structure analysis.

**TABLE S 9B│Markers associated significantly (p = 0.05) with grain zinc content at sub-population level.** (Associations were tested through one way ANOVA of significant MTAs obtained in MLM for grain zinc content).

| **Environment** | **Sub-population** | **Associated markers** |
| --- | --- | --- |
| Del-14 | A | *Xipes* 0180, *Xsinramp* 6 |
|  | B | *Xipes* 0096, *Xpsmp* 2261 |
|  | C | *Xicmp*3004, *Xipes* 0180 |
| Del 15 | A | *Xicmp*3016, *Xsinramp* 6 |
|  | B | *Xpsmp* 2261, *Xsinramp* 6 |
|  | C | ns |
| Del M | A | *Xicmp*3016, *Xipes* 0180, *Xsinramp* 6 |
|  | B | *Xipes* 0096, *Xpsmp* 2261 |
|  | C | *Xicmp* 3004 |
| DW-14 | A | *Xicmp* 4006, *Xsinramp* 6 |
|  | B | *Xipes* 0096, *Xpsmp* 2261 |
|  | C | *Xicmp* 3004, *Xpsmp* 2261, *Xsinramp* 6 |
| DW-15 | A | *Xicmp* 4006 |
|  | B | *Xipes* 0096, *Xpsmp* 2213 |
|  | C | *Xicmp* 3004, *Xpsmp* 2261 |
| DW-M | A | *Xicmp* 4006, *Xsinramp* 6 |
|  | B | *Xipes* 0096, *Xpsmp* 2261 |
|  | C | *Xicmp* 3004, *Xpsmp* 2261, *Xsinramp* 6 |
| Jod-14 | A | *Xicmp* 3004 |
|  | B | *Xpsmp* 2261, *Xipes* 0224 |
|  | C | *Xicmp* 4006, *Xpsmp* 2213 |
| Jod-15 | A | *Xipes* 0180 |
|  | B | *Xipes* 0096, *Xpsmp* 2261 |
|  | C | ns* |
| Jod-M | A | ns |
|  | B | *Xipes* 0096, *Xpsmp* 2261 |
|  | C | ns |
| Y14-M | A | *Xicmp* 3016, *Xsinramp* 6 |
|  | B | *Xipes* 0096, *Xpsmp* 2261 |
|  | C | *Xicmp* 3004, *Xpsmp* 2261 |
| Y15-M | A | *Xsinramp* 6 |
|  | B | *Xipes* 0096, *Xpsmp* 2261 |
|  | C | *Xpsmp* 2261 |
| GM | A | *Xicmp* 3016, *Xsinramp* 6 |
|  | B | *Xipes* 0096, *Xpsmp* 2261 |
|  | C | *Xicmp* 3004, *Xpsmp* 2261 |

*ns means non-significant. Sub-populations are as per Structure analysis.
