# Supplementary material for: Deciphering Genomic Regions for High Grain Iron and Zinc Content Using Association Mapping in Pearl Millet
Source: Front Plant Sci. 2017 May 1;8:412. doi: 10.3389/fpls.2017.00412 (PMC5410614; doi:10.3389/fpls.2017.00412)
Supplement: Table S10A — Phenotypic effect of favorable alleles for grain iron content and top three genotypes carrying them. [file Table10.docx]

**TABLE S 10A │Phenotypic effect of favorable alleles for grain iron content and top three genotypes carrying them.**

| **Favorable allele** | **Environment** | **No. of genotypes carrying the allele** | **PE of the allele** | **Representative genotypes** |
| --- | --- | --- | --- | --- |
| *Xicmp* 3092-220 | DW-14 | 49 | 4.99 | PPMI 1104, PPMI 1090, PPMI 1231 |
| *Xipes* 0096-180 | Y14-M | 30 | 5.45 | PPMI 1102, PPMFeZMP 199, PPMI 1277 |
|  | Del-M |  | 5.12 | PPMI 1102, PPMFeZMP 199, PPMI 683 |
|  | GM |  | 4.79 | PPMI 1102, PPMFeZMP 199, PPMI 683 |
|  | Y15-M |  | 4.13 | PPMI 1102, PPMFeZMP 199, PPMI 683 |
|  | Jod-14 |  | 4.09 | PPMFeZMP 199, PPMI 1102, PPMI 1220 |
| *Xipes* 0180-320 | Del-M | 14 | 12.72 | PPMI 1102, PPMI 1225, PPMI 1108 |
|  | Del-14 |  | 11.12 | PPMI 1102, PPMI 1108, PPMI 1101 |
|  | GM |  | 8.89 | PPMI 1102, PPMI 1108, PPMI 1104 |
|  | Y14-M |  | 8.52 | PPMI 1102, PPMI 1108, PPMI 1104 |
| *Xpsmp* 2209-350 | DW-15 | 54 | 4.05 | PPMI 1102, PPMI 1108, PPMI 1116 |
| *Xpsmp* 2261-180 | Jod-14 | 18 | 19.47 | PPMI 708, PPMFeZMP 199, PIB 228 |
|  | Jod-M |  | 15.67 | PPMFeZMP 199, PPMI 1102, PPMI 708 |
|  | Y14-M |  | 15.46 | PPMI 708, PPMI 1102, PPMFeZMP 199 |
|  | Del-M |  | 14.8 | PPMI 1102, PPMFeZMP 199, PPMI 708 |
|  | GM |  | 12.99 | PPMI 1102, PPMFeZMP 199, PPMI 708 |
|  | Del15 |  | 12.85 | PPMI 1102, PPMI 708, PPMI 683 |
|  | Jod-15 |  | 11.87 | PPMFeZMP 199, PPMI 1102, PPMI 683 |
|  | Y15-M |  | 10.52 | PPMI 1102, PPMFeZMP 199, PPMI 708 |
| *Xsinramp* 6-770 | Del-14 | 28 | 10.97 | PPMFeZMP 199, PPMI 1102, PPMI 708 |
|  | Del-M |  | 9.49 | PPMI 1102, PPMFeZMP 199, PPMI 708 |

PE of allele is Phenotypic effect of an allele. Del-14, Del-15, Jod-14, Jod-15, DW-14, DW-15, Y14-M, Y15-M, Del-M, Jod-M, DW-M and GM are Delhi during 2014, Delhi during 2015, Jodhpur during 2014, Jodhpur during 2015, Dharwad during 2014, Dharwad during 2015, Year 2014 mean, Year 2015 mean, Delhi mean, Jodhpur mean, Dharwad mean and Grand mean respectively.

**TABLE S 10B│Phenotypic effect of favorable alleles for grain zinc content and top three genotypes carrying them.**

| **Favorable allele** | **Environment** | **No. of genotypes carrying the allele** | **PE of the allele** | **Representative genotypes** |
| --- | --- | --- | --- | --- |
| *Xicmp* 3004-210 | Jod-14 | 12 | 5.05 | PPMI 295, PIB 228, PPMI 1112 |
| *Xicmp* 3016-700 | Del-15 | 11 | 1.48 | PPMI 708, PPMI 1101, PPMI 1102 |
| *Xicmp* 4006-280 | DW-15 | 46 | 9.89 | PPMI 1104, PPMI 1105, PPMI 1116 |
| *Xipes* 0096-180 | DW-M | 30 | 4.03 | PPMFeZMP 199, PPMI 1102, PPMI 1067 |
|  | DW-14 |  | 3.25 | PPMFeZMP 199, PPMDMGPM 27, PPMI 1102 |
|  | Y15-M |  | 2.99 | PPMI 1102, PPMFeZMP 199, PPMI 683 |
|  | GM |  | 1.8 | PPMI 1102, PPMFeZMP 199, PPMDMGPM 27 |
|  | Y14-M |  | 0.62 | PPMI 1102, PPMFeZMP 199, PPMDMGPM 27 |
| *Xipes* 0180-320 | Jod-15 | 14 | 7.07 | PPMI 1102, PPMI 1108, PPMI 1225 |
| *Xipes* 0224-190 | Jod-14 | 37 | 1.39 | PPMI 295, PPMI 1102, PIB 228 |
| *Xpsmp* 2086-130 | DW-14 | 113 | 0.23 | PPMI 1104, PPMFeZMP 199, PPMI 708 |
| *Xpsmp* 2213-210 | Jod-M | 11 | 2.76 | PPMFeZMP 126, PPMFeZMP 153, PPMFeZMP 125 |
| *Xpsmp* 2261-180 | Jod-15 | 18 | 11.20 | PPMI 1102, PPMI 295, PIB 228 |
|  | Jod-M |  | 10.85 | PPMI 295, PPMI 1102, PIB 228 |
|  | Jod-14 |  | 10.46 | PPMI 295, PIB 228, PPMI 708 |
|  | Y14-M |  | 8.64 | PPMI 708, PPMI 1102, PPMFeZMP 199 |
|  | Del-14 |  | 7.95 | PPMI 708, PPMFeZMP 199, PPMI 214 |
|  | Y15-M |  | 7.9 | PPMI 1102, PPMI 708, PPMFeZMP 199 |
|  | Del-M |  | 6.78 | PPMI 708, PPMFeZMP 199, PPMI 683 |
|  | GM |  | 6.52 | PPMI 708, PPMI 1102, PPMFeZMP 199 |
| *Xsinramp* 6-770 | DW-14 | 28 | 5.44 | PPMI 1104, PPMFeZMP 199, PPMI 708 |
|  | DW-M |  | 4.89 | PPMI 1104, PPMFeZMP 199, PPMI 1105 |
|  | DW-15 |  | 4.34 | PPMI 1105, PPMFeZMP 199, PPMI 1116 |

PE of allele is Phenotypic effect of an allele. Del-14, Del-15, Jod-14, Jod-15, DW-14, DW-15, Y14-M, Y15-M, Del-M, Jod-M, DW-M and GM are Delhi during 2014, Delhi during 2015, Jodhpur during 2014, Jodhpur during 2015, Dharwad during 2014, Dharwad during 2015, Year 2014 mean, Year 2015 mean, Delhi mean, Jodhpur mean, Dharwad mean and Grand mean respectively.
