# Supplementary material for: Deciphering Genomic Regions for High Grain Iron and Zinc Content Using Association Mapping in Pearl Millet
Source: Front Plant Sci. 2017 May 1;8:412. doi: 10.3389/fpls.2017.00412 (PMC5410614; doi:10.3389/fpls.2017.00412)
Supplement: Table S11A — Top most genotypes carrying favorable alleles for grain iron content. [file Table11.docx]

**TABLE S 11A │Top most genotypes carrying favorable alleles for grain iron content**

| **Genotype** | **Sub pop** | **No. of alleles** | **Alleles** | **phenotypic effect of an allele for Fe** |
| --- | --- | --- | --- | --- |
| PPMI 1102 | B | 5 | *Xipes* 0096-180 | 5.03 |
|  |  |  | *Xipes* 0180-320 | 10.31 |
|  |  |  | *Xpsmp* 2209-350 | 4.05 |
|  |  |  | *Xpsmp* 2261-180 | 14.49 |
|  |  |  | *Xsinramp* 6-770 | 8.74 |
| PPMFeZMP 199 | B | 3 | *Xipes* 0096-180 | 5.03 |
|  |  |  | *Xpsmp* 2261-180 | 14.49 |
|  |  |  | *Xsinramp* 6-770 | 8.74 |
| PPMI 1104 | A | 2 | *Xicmp* 3092-220 | 4.99 |
|  |  |  | *Xipes* 0180-320 | 10.31 |
| PPMI 1108 | C | 2 | *Xipes* 0180-320 | 10.31 |
|  |  |  | *Xpsmp* 2209-350 | 4.05 |
| PPMI 683 | Admix | 2 | *Xipes* 0096-180 | 5.03 |
|  |  |  | *Xpsmp* 2261-180 | 14.49 |
| PPMI 708 | B | 2 | *Xpsmp* 2261-180 | 14.49 |
|  |  |  | *Xsinramp* 6-770 | 8.74 |
| PPMI 1116 | A | 1 | *Xicmp* 2209-350 | 4.05 |
| PPMI 1231 | Admix | 1 | *Xicmp* 3092-220 | 4.99 |
| PPMI 265 | Admix | 1 | *Xctm* 60-235 | 6.12 |
| PPMI 1285 | A | 1 | *Xctm* 60-235 | 6.12 |
| PPMDMGPM 186 | C | 1 | *Xctm* 60-235 | 6.12 |
| PPMI 1277 | A | 1 | *Xipes* 0096-180 | 5.03 |
| PPMI 1101 | A | 1 | *Xipes* 0180-320 | 10.31 |
| PIB 228 | B | 1 | *Xpsmp* 2261-180 | 14.49 |
| PPMI 1220 | B | 1 | *Xipes* 0096-180 | 5.03 |
| PPMI 1090 | Admix | 1 | *Xicmp* 3092-220 | 4.99 |
| PPMI 1225 | B | 1 | *Xipes* 0180-320 | 10.31 |

**TABLE S 11B │Top most genotypes carrying favorable alleles for grain zinc content**

| **Genotype** | **Sub pop** | **No. of alleles** | **Alleles** | **phenotypic effect of an allele for Zn** |
| --- | --- | --- | --- | --- |
| PPMI 1102 | B | 5 | *Xicmp*  3016-700 | 1.48 |
|  |  |  | *Xipes* 0096-180 | 2.54 |
|  |  |  | *Xipes* 0180-320 | 7.07 |
|  |  |  | *Xpsmp* 2261-180 | 8.79 |
|  |  |  | *Xipes* 0224-190 | 1.39 |
| PPMFeZMP 199 | B | 4 | *Xipes* 0096-180 | 2.54 |
|  |  |  | *Xpsmp* 2261-180 | 8.79 |
|  |  |  | *Xpsmp* 2086-130 | 0.23 |
|  |  |  | *Xsinramp* 6-770 | 4.89 |
| PPMI 708 | B | 4 | *Xicmp*  3016-700 | 1.48 |
|  |  |  | *Xpsmp* 2261-180 | 8.79 |
|  |  |  | *Xpsmp* 2086-130 | 0.23 |
|  |  |  | *Xsinramp* 6-770 | 4.89 |
| PIB 228 | B | 3 | *Xicmp* 3004-210 | 4.81 |
|  |  |  | *Xipes* 0224-190 | 1.39 |
|  |  |  | *Xpsmp* 2261-180 | 8.79 |
| PPMI 295 | B | 3 | *Xicmp* 3004-210 | 4.81 |
|  |  |  | *Xipes* 0224-190 | 1.10 |
|  |  |  | *Xpsmp* 2261-180 | 8.79 |
| PPMI 1104 | A | 3 | *Xicmp* 4006-280 | 6.54 |
|  |  |  | *Xpsmp* 2086-130 | 0.23 |
|  |  |  | *Xsinramp* 6-770 | 4.89 |
| PPMI 1105 | Admix | 2 | *Xicmp* 4006-280 | 6.54 |
|  |  |  | *Xsinramp* 6-770 | 4.89 |
| PPMI 1116 | A | 2 | *Xicmp* 4006-280 | 6.54 |
|  |  |  | *Xsinramp* 6-770 | 4.89 |
| PPMI 683 | Admix | 2 | *Xipes* 0096-180 | 2.54 |
|  |  |  | *Xpsmp* 2261-180 | 8.79 |
| PPMFeZMP 126 | C | 1 | *Xpsmp*-2213-210 | 2.76 |
| PPMDMGPM 27 | A | 1 | *Xipes* 0096-180 | 2.54 |

contd..

| **Genotype** | **Sub pop** | **No. of alleles** | **Alleles** | **phenotypic effect of an allele for Zn** |
| --- | --- | --- | --- | --- |
| PPMI 214 | B | 1 | *Xpsmp* 2261-180 | 8.79 |
| PPMI 1108 | C | 1 | *Xipes* 0180-320 | 7.07 |
| PPMFeZMP 125 | C | 1 | *Xpsmp*-2213-210 | 2.76 |
| PPMFeZMP 153 | C | 1 | *Xpsmp*-2213-210 | 2.76 |
| PPMI 1067 | A | 1 | *Xipes* 0096-180 | 2.54 |
| PPMI 1101 | A | 1 | *Xicmp*  3016-700 | 1.48 |
| PPMI 1112 | A | 1 | *Xicmp* 3004-210 | 4.81 |
| PPMI 1225 | B | 1 | *Xipes* 0180-320 | 7.07 |
