# Supplementary figures and images for: Deciphering Genomic Regions for High Grain Iron and Zinc Content Using Association Mapping in Pearl Millet
Source: Front Plant Sci. 2017 May 1;8:412. doi: 10.3389/fpls.2017.00412 (PMC5410614; doi:10.3389/fpls.2017.00412)

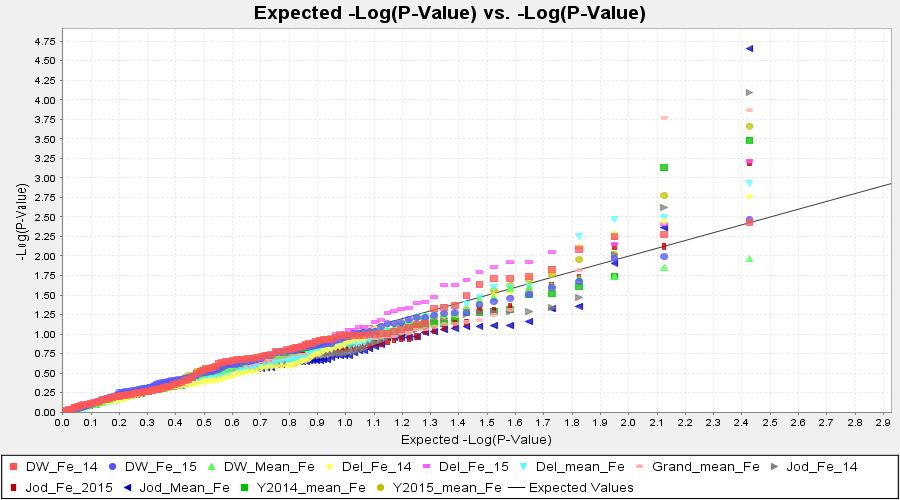

Supplement: Figure S1 — Quantile-Quantile (QQ) plots for grain iron content in MLM showing distribution of marker-trait association. [file Image1.JPEG]

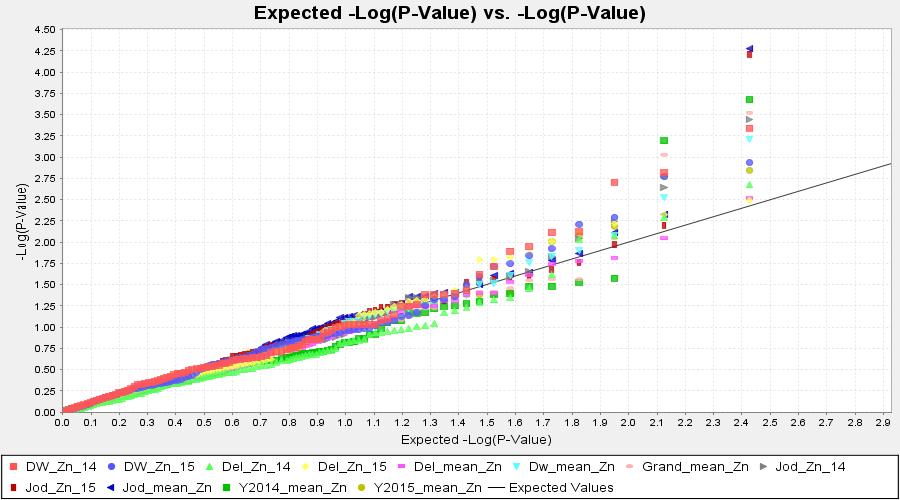

Supplement: Figure S2 — Quantile-Quantile (QQ) plots for grain zinc content in MLM showing distribution of marker-trait association. [file Image2.JPEG]
